# Quantum-Enhanced Edge Intelligence Leveraging Large Language Models for Immersive Space–Aerial–Ground Communications: Survey, Challenges, and Open Issues

**DOI:** 10.3390/s26041181

**Published:** 2026-02-11

**Authors:** Abhishek Gupta, Ajmery Sultana

**Affiliations:** Faculty of Computer Science and Technology, Algoma University, Brampton, ON L6V 1A3, Canada; ajmery.sultana@algomau.ca

**Keywords:** unmanned aerial vehicles, vehicular communications, 6G, CubeSat, LLM, IMT-2030, space–aerial–ground integrated networks (SAGIN), latency minimization, quantum communication

## Abstract

**Highlights:**

**What are the main findings?**

- Quantum-enhanced LLMs improve adaptive, high-throughput, and context-aware decision-making across UAV, CubeSat, and terrestrial nodes in SAGIN, enhancing energy efficiency, reliability, and edge learning in 6G networks.
- The integration of UAVs, CubeSats, and terrestrial infrastructures with LLM-driven quantum edge intelligence overcomes classical challenges in bandwidth allocation, dynamic routing, and interoperability, enabling secure, privacy-preserving, and self-optimizing 6G communication systems.

**What is the implication of the main finding?**

- The integration of quantum-enhanced LLMs into SAGIN enables efficient, reliable, and adaptive communication systems, facilitating ultra-low latency and high-throughput 6G services across UAV, CubeSat, and terrestrial networks.
- By overcoming classical limitations in bandwidth allocation, dynamic routing, and interoperability, quantum-empowered LLMs support secure, privacy-preserving, and self-optimizing intelligent transportation amalgamated with next-generation communication systems.

**Abstract:**

The integration of unmanned aerial vehicles (UAVs), autonomous vehicles, and advanced satellite systems in sixth-generation (6G) networks is poised to redefine next-generation communications as well as next-generation intelligent transportation systems. This paper examines the convergence of UAVs, CubeSats, and terrestrial infrastructures that comprise the framework of Space–Aerial–Ground Integrated Networks (SAGINs) as vital enablers of the International Mobile Telecommunications (IMT)-2030 standards. This paper examines the role of UAVs in providing flexible and quickly deployable airborne connectivity. It also discusses how CubeSats enhance global coverage through low-latency relaying and resilient backhaul links from low Earth orbit (LEO). Additionally, the paper highlights how terrestrial systems contribute high-capacity, densely concentrated communication layers that support various end-user applications. By examining their interoperability and coordinated resource allocation, the paper underscores that the seamless interaction of SAGIN nodes is essential for achieving the ultra-reliable, intelligent, and pervasive communication capabilities envisioned by IMT-2030. As 6G aims for ultra-low latency, high reliability, and massive connectivity, UAVs and CubeSats emerge as key enablers for extending coverage and capacity, particularly in remote and dense urban regions. Furthermore, the role of large language models (LLMs) is explored for intelligent network management and real-time data optimization, while quantum communication is analyzed for ensuring security and minimizing latency. The integration of LLMs into quantum-enhanced edge intelligence for SAGINs represents an emerging research frontier for adaptive, high-throughput, and context-aware decision-making. By exploiting quantum-assisted parallelism and entanglement-based optimization, LLMs enhance the processing efficiency of multimodal data across space, aerial, and terrestrial nodes. This paper further investigates distributed quantum inference and multimodal sensor data fusion to enable resilient, self-optimizing communication systems comprising a high volume of data traffic, which is a critical bottleneck in the global connectivity transition. LLMs are envisioned as cognitive control centers capable of generating semantic representations for mission-critical communications that enhance energy efficiency, reliability, and adaptive learning at the edge. The findings of the survey reveal that quantum-enhanced LLMs overcome challenges pertaining to bandwidth allocation, dynamic routing, and interoperability in existing classical communication systems. Overall, quantum-empowered LLMs significantly assist intelligent, autonomous, and immersive communications in SAGIN, while enabling secure, privacy-preserving communication.

## 1. Introduction

The sixth-generation (6G) of wireless communication networks is expected to revolutionize connectivity by providing reliable, nearly instantaneous channel access and data transfer capabilities [1,2]. 6G will unify terrestrial, aerial, maritime, and space-based communications into a seamless system capable of supporting massive connectivity with ultra-low latency and high reliability [3,4]. UAV–CubeSat–vehicle communication systems will play a key role in this architecture, enabling real-time applications in intelligent transportation, remote sensing, and disaster management through space–terrestrial integration [5,6]. Aligned with the International Mobile Telecommunications (IMT)-2030 vision, major challenges for 6G networks include optimizing hardware and software, managing computational complexity, embedding multi-access edge computing (MEC) nodes, developing automated solutions, and establishing virtual radio access network (RAN) interfaces [7]. The efficient delivery and processing of transmitted data packets and inferring their content also requires understanding user context and intent [8].

Recent advances in machine learning (ML) and artificial intelligence (AI) have driven significant developments in applications such as self-driving vehicles, which generate vast amounts of sensor and camera data that demand substantial computational power for real-time decision-making [9,10,11]. Applications in holographic telepresence, immersive environments, Industry 4.0, large-scale robotics, augmented reality (AR), virtual reality (VR), and extended reality (XR) are expected to be central to 6G networks, requiring the real-time transmission and processing of high-dimensional data, including high-resolution video and audio streams [3]. However, meeting these demands over bandwidth-constrained wireless channels and resource-limited end-user devices presents significant challenges [12]. The high-precision, multimodal sensor data generated by 6G systems will produce vast volumes of network information, necessitating efficient data collection and analytics for effective monitoring. As data volumes and processing demands increase, energy efficiency becomes a critical design objective, particularly to sustain performance while minimizing resource consumption [13]. Distributed data processing at the edge and in fog computing nodes can reduce latency and core network traffic, as edge devices with advanced processors and storage collaborate in fog pools to enable more energy-efficient system-level operation through localized processing and reduced backhaul communication [14].

To address these challenges, large language models (LLMs) have been increasingly explored in communication networks for tasks such as intelligent network management, adaptive control, and content personalization [15]. While LLMs are inherently computationally intensive, their integration into communication systems is primarily motivated by their ability to optimize network operations and decision-making, which can indirectly contribute to improved energy efficiency at the system level. For example, LLM-enabled cognitive and autonomic functions can enhance fault handling, security, compliance, and resource orchestration, potentially reducing unnecessary signaling, redundant processing, and inefficient resource allocation [16]. LLMs are emerging as foundational enablers of intelligent systems in space–aerial–ground integrated networks (SAGINs), where their large-scale reasoning, adaptive learning, and multimodal understanding support immersive communications in distributed and heterogeneous environments [17]. Nevertheless, assessing the performance, robustness, adaptability, and energy overhead of LLMs, particularly when deployed in quantum-enhanced edge intelligence, remains an open research challenge. Moreover, according to the limitations outlined in the existing state-of-the-art works, existing benchmarks are often constrained by limited scope, static configurations, and insufficient representation of real-world network dynamics. This paper explores the applicability of LLMs in 6G wireless communications, highlighting key LLM-based methods and analyzing how their integration with quantum-enhanced edge architectures impacts immersive communications in connected and autonomous vehicles, while explicitly considering associated system-level trade-offs [18,19].

The emergence of 6G networks is also expected to generate vast amounts of sensor data that must be collected and analyzed with strict time-bound constraints. The timely inference of high-precision sensor data is crucial for managing mission-critical applications, requiring uninterrupted and complete coverage as well as continuous data collection. The generation of sensor data for every packet at each node results in data volumes that surpass available channel and computational resources, imposing substantial processing and bandwidth constraints [20]. While smart sensors are programmable and adaptable, enabling end-to-end monitoring across various segments including cloud and RAN, they still require integration with LLMs to provide relevant, context-aware data. This involves in-network preprocessing to aggregate multimodal sensor data, adjust data resolution, and support query-based extraction [21].

In future 6G wireless communication networks, LLMs are expected to orchestrate coordination across space, aerial, and terrestrial nodes, facilitate multimodal data fusion, and sustain network stability under dynamic conditions, establishing a reference framework for next-generation intelligent communication systems [22]. This integration enables agents to adapt to tasks, perform real-time semantic interpretation, and make intelligent decisions across distributed SAGIN infrastructure. State-of-the-art LLMs demonstrate consistent reasoning under variable signal conditions, latency constraints, and resource-limited edge nodes, while quantum-assisted optimization further enhances inference accuracy and energy efficiency [23]. The framework also supports continual learning, allowing models to autonomously adapt to evolving network contexts. These findings highlight quantum-enhanced LLMs as the cognitive core of future autonomous communication networks, advancing reliability, scalability, and intelligence [24]. Furthermore, combining quantum communication and ML with post-quantum cryptography, including protocols like BB84, significantly strengthens security against emerging cyber threats. This software-driven approach, integrated with AI and ML, ensures secure communication across UAV, CubeSat, and vehicle networks by leveraging quantum-safe mechanisms and blockchain-based trust frameworks [25]. Integrating quantum computing additionally enables large-scale parallelism and entanglement-based optimization, ensuring both efficiency and scalability.

### 1.1. Contributions

The objective of this survey article is to systematically review and synthesize recent research at the intersection of quantum computing, LLMs, and SAGINs in the context of 6G communications. This paper analyzes existing approaches that explore the use of quantum-enhanced LLMs for distributed network management, latency-aware operation, privacy preservation, and adaptive decision-making across heterogeneous infrastructures, including UAVs, CubeSats, and terrestrial nodes. The survey examines the current design paradigms, performance trade-offs, and limitations, and provides a structured discussion of how quantum-enabled edge intelligence may support semantic understanding, dynamic optimization, and self-organizing behavior in complex, data-intensive communication environments. Through the synergistic integration of LLMs and quantum communication paradigms, this paper provides a foundation for autonomous, resilient, and sustainable 6G network ecosystems. By consolidating state-of-the-art methods and identifying open challenges and research directions, this survey offers a comprehensive perspective on the potential role of quantum-enhanced LLMs in enabling autonomous, resilient, and sustainable 6G network ecosystems. An extensive review of LLMs is provided in [26]. The principal contributions of this survey are summarized as follows:

(a) We present a comprehensive survey of recent research on the integration of quantum-assisted LLMs into SAGINs, with a focus on their role in enabling adaptive, real-time network management and decision-making for 6G environments.
(b) We review and analyze existing studies on distributed LLM inference and quantum-enhanced intelligence across heterogeneous nodes, including UAVs, CubeSats, and terrestrial platforms, highlighting their implications for latency, reliability, scalability, and resource efficiency.
(c) We examine state-of-the-art approaches to quantum-assisted multimodal data fusion in SAGIN-enabled vehicular and aerial communication systems, emphasizing their impact on energy efficiency, bandwidth utilization, interoperability, and robustness in heterogeneous network scenarios.
(d) We survey LLM-driven edge intelligence models deployed at aerial and space nodes, such as UAVs and CubeSats, and analyze their capabilities in supporting context-aware learning, autonomous optimization, and self-organizing network behavior in dynamic and heterogeneous 6G environments.
(e) We consolidate and compare performance metrics, evaluation methodologies, and benchmarking frameworks used in the literature to assess quantum-enhanced LLM integration in 6G SAGIN architectures, identifying limitations of existing evaluations and open challenges for future research.
(f) Based on the surveyed literature, we identify key research gaps and outline future research directions, including quantum-secure LLM-enabled communication, sustainable and energy-efficient network design, and trust-aware semantic control mechanisms for mission-critical 6G applications.

Table 1 summarizes existing works and highlights gaps in the performance analysis of UAV, CubeSat, and LLM-based 6G SAGINs, along with the methodologies employed and our proposed approach to address these gaps. In Table 1, we identify specific limitations in existing studies, including restricted scalability in dynamic UAV networks, the insufficient handling of heterogeneous multimodal data across space, aerial, and terrestrial nodes, and the limited integration of intelligent edge processing with quantum-enhanced LLMs. We further investigate resource allocation and optimization strategies to enhance end-to-end system performance while maintaining energy efficiency and reliability. Table 1 highlights our contributions, clarifies the research gaps we address, and underscores the novelty and practical relevance of this survey. Additionally, we recognize that much of the data processed at UAV or terrestrial edge nodes originates from heterogeneous sensors embedded in vehicles and CubeSats, resulting in non-independent and identically distributed (non-i.i.d.) data. Our investigation of SAGIN performance under non-i.i.d. data distributions represents a novel contribution of this work, demonstrating how quantum-enhanced LLMs can enable resilient, self-optimizing, and intelligent 6G communication systems.

Unlike existing works, this survey employs a layered taxonomy that organizes the literature into four interconnected dimensions as follows:

1. Intelligence Layer: focuses on LLMs and multimodal foundation models for perception, reasoning, and decision-making at the network edge.
2. System and Computing Layer: covers edge–cloud collaboration, distributed intelligence, and digital twins for SAGINs.
3. Communication and Networking Layer: Addresses immersive SAGIN communications, non-terrestrial networks, and alignment with 6G IMT-2030 requirements.
4. Quantum-Enhanced Layer: Examines the role of quantum communications and quantum intelligence in enhancing security, coordination, and performance in edge intelligence systems.

This survey emphasizes system-level architectures and open research challenges at the intersection of LLM-driven edge intelligence and quantum-enhanced SAGIN communications, rather than providing exhaustive coverage of standalone quantum communication protocols or general LLM training methodologies. This survey does not aim to:

- Provide an exhaustive review of standalone quantum communication protocols or quantum hardware implementations;
- Survey general-purpose LLM architectures or training methodologies that are not related to or applied to communication systems;
- Address low-level physical-layer modeling in isolation from intelligent networking or edge intelligence;
- Benchmark specific commercial platforms or provide experimental performance evaluations.

Note, while several state-of-the-art surveys have explored SAGIN architectures, edge intelligence, and semantic communications independently, our manuscript provides a unique, integrated perspective by specifically focusing on the convergence of quantum-enhanced edge intelligence with LLMs for immersive SAGIN applications. Unlike prior works that treat LLMs, edge AI, or semantic communications in isolation, our survey highlights:

- The role of LLMs as distributed cognitive agents enabling context-aware learning, autonomous optimization, and adaptive network management in highly dynamic SAGIN environments.
- By integrating quantum computing and quantum communications with edge intelligence, this work builds upon existing surveys and offers new insights into latency, reliability, and secure multi-node coordination.
- A unified discussion of semantic-driven multimodal sensing, retrieval-augmented generation (RAG), and task-oriented decoding in the context of SAGINs, bridging communication, computation, and AI for real-time decision-making.
- A comprehensive mapping of research challenges, deployment gaps, performance evaluation, and feasible quantum–classical hybrid architectures that provide actionable guidance for future 6G and IMT-2030 network design.

By explicitly examining these intersections and presenting quantitative and architectural insights, this survey delineates a perspective that distinguishes it from prior works, providing both a conceptual framework and actionable guidelines for the deployment of quantum-enhanced LLMs in SAGIN environments.

### 1.2. Organization

**Figure 1:**
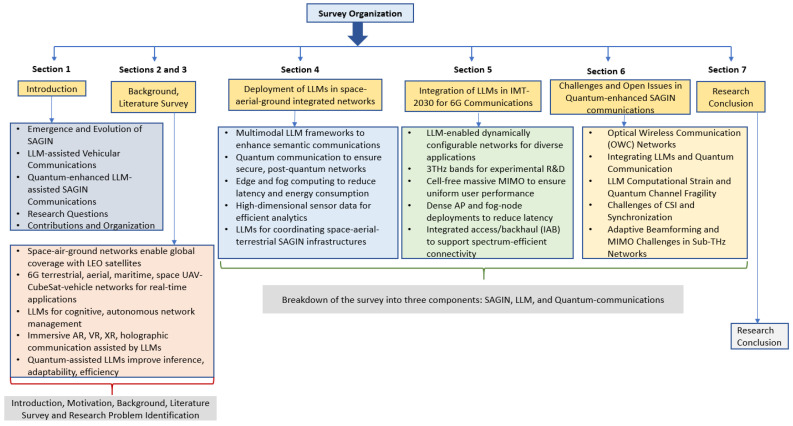
The organization of this survey.

The rest of the paper is organized as follows. Section 2 provides a discussion of existing approaches in SAGIN-assisted vehicular communications with an emphasis on low-latency applications. Section 3 introduces the effectiveness and applicability of LLMs in heterogeneous networks. Section 4 discusses the deployment of LLMs in SAGIN. Section 5 elaborates on the integration of LLMs into the IMT-2030 framework envisioned for 6G communications. Section 6 explores quantum-enhanced communication paradigms for next-generation communication systems, highlights critical open issues in SAGINs and proposes some open issues and avenues for future research. Finally, Section 7 concludes the paper. Figure 1 illustrates the organization of this survey.

## 2. Low-Latency Applications in Quantum-Enhanced Space–Aerial–Ground Networks

**Figure 2:**
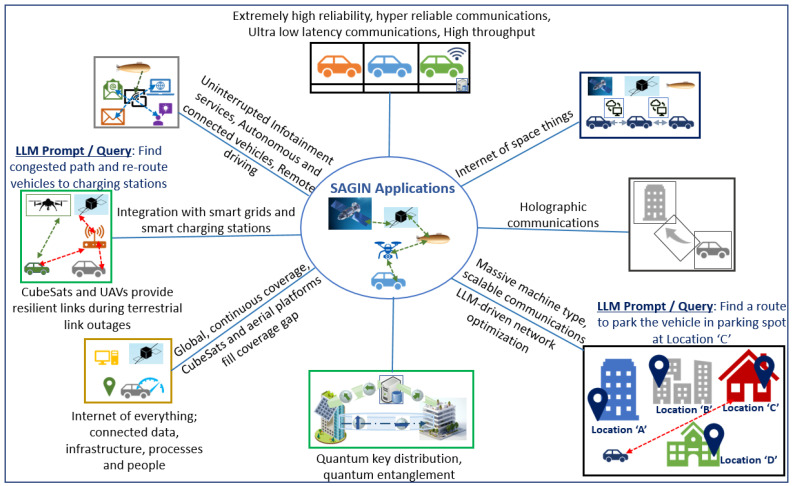
The figure illustrates a conceptual architecture for integrating LLMs and quantum communication in SAGIN for 6G vehicular communications. The figure depicts the interaction between terrestrial vehicles, UAVs, and CubeSats, along with edge and cloud components, and highlights the roles of LLM-based multimodal data processing. The figure provides a high-level view of how some of the emerging technologies may be jointly incorporated in dynamic SAGIN environments.

It is envisaged that 6G will need to be dynamically configurable in real-time to adapt to the diverse application requirements included in the Third generation partnership project (3GPP) Release 17. The LLM-integrated semantic communications framework utilizes a device–edge architecture for immersive 6G applications such as AR, VR, XR, holographic communications, and autonomous driving. LLMs excel in these applications that generate large-scale and high-dimensional data. For example, in smart cities, LLMs integrate digital information with the physical environment in real time. As users navigate, their movements are tracked to deliver relevant AR and VR data, such as three-dimensional (3D) building models or traffic updates, with content prioritized based on the user’s needs or goals [27]. User intent in this context helps prioritize the information that is most relevant to the user’s current task requirements or actions. For example, if a user is navigating through a city, their actions such as avoiding traffic or finding a specific location guides the LLM to prioritize specific data such as alternative routes or nearby points of interest [28]. Figure 2 illustrates the integration of LLMs across textual, speech, and video data processing, and highlights the role of quantum communication in reducing latency and power consumption while improving throughput. It highlights how communication efficiency and energy consumption can be optimized in dynamic SAGIN-assisted 6G vehicular networks while meeting the stringent performance requirements of novel and futuristic 6G applications.

The 6G air interface is expected to integrate AI-native radio functions, as outlined by the International Telecommunication Union (ITU), and is being actively pursued by the 3GPP [29]. In Release 18, the 3GPP studied AI and ML integration into the 5G new radio air interface, focusing on use cases such as channel state information (CSI) feedback and beam management. Release 19 aimed to expand these efforts, addressing prevailing issues and exploring AI- and ML-based mobility management [30]. 3GPP Release 17 established foundational principles for AI-enabled RAN intelligence in energy saving, load balancing, and mobility optimization, while recognizing that AI models are implementation-specific. Release 18 enhanced data collection and signaling support, while Release 19 introduced AI and ML support for quantum aware resource allocation and entanglement-assisted communication schemes [31]. Researchers are assessing quantum communication link performance under realistic conditions, focusing on fidelity degradation due to noise and AI-assisted scheduling. A wireless network digital twin platform is essential for accurately modeling link fidelity and quantum behaviors in realistic radio environments [32].

In holographic communications, LLMs enable the real-time transmission of high-fidelity 3D representations by inferring user intent from gestures, facial expressions, and voice patterns, thereby ensuring seamless and natural immersive interactions [33]. For example, in a virtual business meeting within a holographic conference room, participants are represented by high-fidelity 3D avatars. As a speaker gestures, the LLM infers intent by analyzing hand movements, facial expressions, and vocal patterns, and dynamically prioritizes key visual elements such as the face or hands to ensure optimal clarity. This enables more natural and effective interactions by improving the real-time interpretation of verbal and non-verbal cues [34]. Furthermore, LLMs support clustering vehicles with separate interfaces or utilizing over-the-air bidirectional signaling to minimize fronthaul reliance.

### 2.1. Emergence of Space–Aerial–Ground Integrated Communications

Space–aerial–ground integrated communications also aim to bridge the digital divide between urban and rural areas. While advanced access technologies primarily benefit densely populated regions, rural areas often experience limited gains [35]. The emphasis on higher peak data rates tends to favor users located near dense radio access infrastructures [36]. For example, massive multiple-input multiple-output (MIMO) can enhance cell–edge performance and theoretically serve up to 3000 homes within an 11 km radius; however, its deployment remains constrained by the economic viability of sparsely populated regions [37]. Moreover, achieving user fairness under limited resources may reduce overall data rates. This highlights the need for access methods that deliver uniform capacity over large areas while minimizing per-user costs, particularly in challenging rural environments [38].

### 2.2. Multimodal Large Language Models (MLLMs)

In SAGINs, LLMs perceive the environment, infer user intent, and extract key semantic features for tasks. Prompt engineering and in-context learning enable efficient adaptation to dynamic wireless environments and resource constraints. Recent research integrates LLMs into semantic communication systems, using segmentation models and adaptive compression to transform visual data into captioned images with importance weighting and error correction [39]. For instance, in autonomous vehicle navigation, LLMs combine visual and textual features via attention mechanisms to improve image reconstruction [40]. Despite these advancements, incorporating LLMs into semantic communication frameworks remains challenging, requiring redesigned encoders and decoders to fully exploit large multimodal models in 6G networks spanning terrestrial and satellite systems.

The multimodal large language model (MLLM) framework leverages pre-trained networks for context-aware, task-oriented wireless communication. In a device–edge collaborative architecture, MLLM-based semantic guidance modules analyze multimodal inputs, user intents, and channel conditions to generate attention maps that prioritize critical information for transmission. An importance-aware semantic encoder and resource-adaptive decoder optimize bandwidth allocation and produce high-quality content [41]. Case studies in visual question answering for AR/VR and diffusion-driven image generation demonstrate the framework’s effectiveness. By focusing on semantically relevant features rather than raw signals [42], MLLMs enhance multimodal understanding, reasoning, and data generation, addressing the limitations of generative AI techniques such as variational autoencoders and GANs in complex vehicular communication environments [21].

### 2.3. Storage Capacity Limitations of Mobile Vehicular Edge Servers

The increasing storage capacity of mobile vehicular edge servers makes coded caching beneficial, given the higher popularity of limited, location-dependent content. Designing efficient multi-cast and broadcast strategies remains a research challenge due to diverse channel conditions and performance complexities [43]. Edge computing transfers processing and data storage from central clouds to nodes close to data sources, enhancing performance and enabling ultra-low latency. The multi-access edge computing (MEC) initiative connects edge applications with cellular networks, providing access to base station data. Edge intelligence allows data analysis and action near its source, minimizing latency and costs while enhancing security [44]. This requires the local processing and filtering of information, enabling nodes to learn and share insights collectively, optimizing services. As 6G evolves, it is expected to shift AI intelligence from centralized systems to edge computing [45].

In many applications, coded caching enhances data rates by using cache memories distributed across a network, providing both global and local caching gains. Global caching gains depend on the total cache size of all vehicles, while local gains come from individual vehicle caching. By multi-casting codewords to groups of vehicles with each node containing relevant data for the group, the resulting data transmission over the broadcast link is reduced by a factor of (1+t), where $\left(t=\frac{KM}{N}\right)$ with (K) as the number of vehicles, (M) as the cache size per vehicle, and (N) as the file library size. This gain is achievable in multi-antenna communications, where multi-cast beamforming suppresses interference between overlapping coded caching codewords [46]. When vehicles interact with network-based applications demanding high multimedia traffic and QoS, combining caching with computation offloading to the network edge yields high-throughput, low-latency results [47].

Traditional AI methods are resource intensive, and the growing demand for real-time performance challenges current computational architectures. Deep neural network processing, relying on matrix multiplications, benefits from photonic computing [48] and in-memory computing [49], which reduces data-transfer bottlenecks by performing computations near memory. System-on-chip designs, such as adaptive computing acceleration platforms (ACAPs), combine CPUs, AI engines, and programmable logic with high-speed interconnects and on-chip memory, complementing photonic and in-memory approaches while enabling AI processing without off-chip transfers [45]. Edge computing spans hardware, infrastructure, and platform layers, each with unique challenges, and the European Edge Computing Consortium promotes adoption via reference architectures and best practices [50].

### 2.4. Cloud–Edge Integration for End-to-End Low-Latency Collaborative Intelligence

Distributed, low-latency, and reliable machine learning at the network edge is essential for mission-critical applications. Edge AI enables intelligence-driven orchestration across constrained platforms, requiring effective synergy among devices, edge nodes, and cloud infrastructure to extract insights while preserving performance, privacy, and security [51]. As computing evolves from cloud computing to cloud intelligence, the Internet of Things (IoT) transitions to the Internet of Intelligent Things (IoIT), enhancing reliability and efficiency [52].

Several models for cloud–edge integration enhance processing and data management. In Cloud–Edge Co-Inference with Cloud Training, inference is shared between edge and cloud while training occurs in the cloud. In-Edge Co-Inference with Cloud Training executes inference at the edge with partial or full data offloading, while training remains cloud based [53]. On-Device Inference with Cloud Training keeps inference on the device, with no offloading. Cloud–Edge Co-Training and Inference distributes both training and inference between cloud and edge. In-Edge Operation performs both processes at the edge, and Edge-Device Co-Training and Inference executes them entirely on local devices [53]. Together, these models offer strategies for optimizing cloud–edge integration [54].

### 2.5. Energy Consumption of LLMs on UAVs

Deploying LLMs on UAVs poses significant energy consumption challenges due to limited onboard power. Running moderately sized LLMs (1 to 2 billion parameters) can consume tens to hundreds of watts, influenced by inference frequency, model size, and optimization methods. To enable effective LLM deployment, techniques such as model compression, quantization, knowledge distillation, and hardware acceleration such as edge-computing-based AI chips are essential. Additionally, offloading tasks to edge servers or utilizing collaborative UAV swarms helps reduce onboard energy consumption while maintaining low-latency inference for real-time applications. Deploying LLMs at the network edge presents significant challenges due to the limited computational power, memory, and energy resources available on devices such as UAVs, CubeSats, and autonomous vehicles. LLMs typically require high-performance hardware and substantial computational energy, which is often not feasible for edge nodes.

To overcome these limitations, several strategies can be employed, including model compression, pruning, quantization, knowledge distillation, and the use of low-rank or sparse attention mechanisms [55]. Additionally, split inference, a technique where subsets of the model are partly run across both edge and cloud nodes, can help reduce latency and energy consumption. By implementing these strategies, it becomes possible to deploy LLMs effectively at the edge, enabling context-aware decision-making and adaptive intelligence while accommodating the practical constraints of resource-limited environments in SAGIN environments [56]. Table 2 summarizes estimating energy consumption of LLMs on UAV platforms. Integrating these energy considerations into SAGIN system design is crucial for sustainable and reliable UAV-assisted networks.

## 3. Effectiveness and Applicability of LLMs in Immersive SAGIN Environments

The effectiveness of LLMs in wireless networks depends on architecture, node cooperation, and the performance–cost trade-off. LLM-driven communication synchronizes data acquisition, multiple access, resource management, and signal encoding, supported by fast collaboration between cloud and edge computing [57]. At the edge, LLMs automate resource allocation, enable real-time learning and inference, and support location-based optimization to improve coverage and quality of service (QoS) while respecting privacy [58]. Cooperative intelligence and fog computing enhance task handover, computational capacity, and algorithm efficiency across heterogeneous platforms, though energy efficiency for in-vehicle and edge servers remains critical for sustainable vehicular communications [27,59].

Embedding computational hardware in existing infrastructure is essential for edge-deployed LLMs. Small language models (SLMs) face challenges in generalizing from limited data, making duplicate detection and anomaly detection crucial to prevent overfitting [60]. Vehicular sensors generate diverse multi-sensor data, which can be inconsistent across large networks, requiring LLMs with adaptive capabilities [61]. Federated learning (FL) reduces communication overhead and improves scalability by sharing processed data or trained models rather than raw data. Effective data preprocessing and edge-server clustering for similarity and anomaly detection enable reliable LLM performance across edge, cloud, and centralized infrastructures [62,63].

### 3.1. Synthetic Data Generation via GANs for LLM Pre-Training in Sparse-Data SAGIN Scenarios

**Figure 3:**
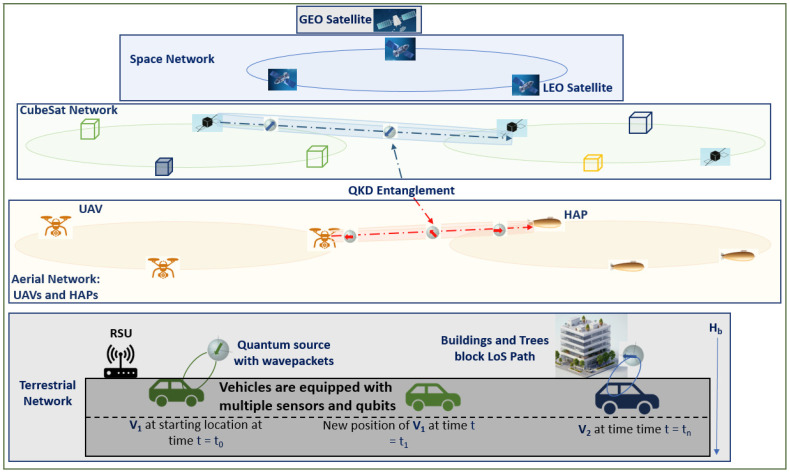
A general SAGIN architecture for 6G vehicular communications. Vehicle sensor data is typically processed at in-vehicle edge servers or offloaded to cloud servers. Recent works integrate sensor data processing with LLMs or propose using qubits instead of classical hardware to meet the stringent performance requirements of emerging 6G applications.

Synthetic data generation with generative adversarial networks (GANs) and incremental learning improves LLM accuracy, while heterogeneous data fusion at the edge leverages feature extraction, representation learning, and split learning [64]. However, the capabilities of LLMs are heavily dependent on the computational capacity of processing servers and the efficiency of underlying communication links. Edge application placement is critical for real-time adaptability, as vehicles interact with mobile cloud and edge platforms. Interoperability, distributed LLM deployments, and mobile SAGIN nodes introduce latency, power, and storage constraints [59,65]. Lightweight LLMs (SLMs), microservices, and virtual machines help balance pre-trained and online-learned models. Energy-efficient, low-complexity, and privacy-sensitive designs are essential for distributed LLMs [66]. Limited data availability requires exchanging raw data, model parameters, or inferences under communication uncertainties [67]. User and resource scheduling as well as data and model communication are therefore critical. Figure 3 illustrates a general SAGIN architecture, where vehicle sensor data is processed at in-vehicle edge servers, offloaded to cloud servers, or, in recent research, on qubits rather than classical hardware.

### 3.2. Understanding User Context and Analyzing Behavioral Patterns with LLMs

Understanding user context and node behavior in SAGIN is critical for adapting edge resources to dynamic demands. The mobility of terrestrial nodes, UAVs, and CubeSats challenges handover latency and virtual resource management, while multi-tenant environments raise privacy concerns [68]. Virtualization and container technologies enable flexible resource allocation, supporting reliable deployment for AR/VR/XR applications, which require quality metrics beyond throughput and latency [69]. LLM-based payload customization prioritizes critical data [70,71], enhances cross-layer control, and supports self-organizing, self-healing networks with analytics at the core and edge [72,73]. Deterministic networking, time-sensitive networking, and segment routing provide low-latency, ultra-reliable communications, while automated vRAN and MEC integration optimize resource allocation and network management [74,75].

### 3.3. Collaborative Analytics Across SAGINs Using Distributed LLM Agents

Current wireless networks rely on DL models for system representation, but traditional DL approaches are computationally intensive and insufficient for 6G performance requirements [76]. LLMs enhance automated service negotiation, decision-making, and QoS management [42]. A scalable, LLM-enabled framework can manage diverse systems including satellites and mobile edge networks while providing context-aware and location-aware features such as per-packet latency tracking, customized connectivity, and payload optimization [39].

### 3.4. Key Findings

Recent studies highlight that LLM-based collaborative analytics across SAGIN nodes improve accuracy and decision-making. In connected and autonomous vehicles, edge computing (e.g., MEC) reduces latency and energy consumption, with specialized LLM-optimized graphical processing units (GPUs) enhancing processing capabilities [77]. LLMs enable selective edge processing, transmitting only relevant data to reduce network load and ensure high-precision, low-latency performance for emerging applications such as holographic teleportation, AR, VR, and XR [78,79]. Cloud-native 6G infrastructure with virtualization, microservices, and LLM-driven intelligence supports context-aware aggregation, semantic processing, and self-organizing networks [80]. Table 3 summarizes the recent works, methodologies, gaps, and key challenges including data heterogeneity, communication constraints, mobility, privacy, and resource limitations, emphasizing the need for scalable, automated, and LLM-enabled edge computing frameworks.

## 4. Challenges in Deployment of LLMs in SAGINs

The recent literature highlights the potential of LLMs in 6G networks to model complex interactions that traditional deep learning (DL) models cannot capture, enabling real-time analysis, automated operations, and intelligent resource management [81,82]. Timely data from devices is crucial for latency-sensitive applications such as video monitoring and holographic communications, while efficient data transfer avoids transmitting raw or redundant data [83,84]. LLMs can be deployed across management, core, and edge devices, learning system characteristics for tasks like classification and regression [85]. Conventional DL requires large datasets and struggles with network heterogeneity and data confidentiality, making probabilistic and Bayesian methods, including Gaussian processes, Variational Bayes, and MCMC, essential for scalable, uncertainty-aware 6G analytics [86,87,88].

Reproducing kernel Hilbert space features improve DL models by providing well-regularized inputs with fewer hyperparameters. FL allows mobile devices to train shared models without transmitting raw data, though wireless impairments and limited bandwidth affect performance [89]. DL-based positioning relies on signals from devices but suffers from NLoS multipaths, missing CSI and received signal strength (RSS) measurements, and environmental variability [90,91]. Channel estimation in SAGIN is challenged by nonlinear and non-stationary channels, and offline-trained models degrade in real-world conditions [92]. Non-convex optimization problems, including throughput maximization and beamforming, are computationally intensive; heuristics reduce complexity but are suboptimal [78]. Table 4 summarizes key research on LLMs and DL in 6G wireless networks, covering network control, resource management, channel estimation, and multi-user positioning. Critical gaps include latency-sensitive data dependency, limited large-scale datasets, cross-operator interoperability, computational complexity, and scalability in dynamic and high-dimensional environments.

### 4.1. Advantages of LLMs over Conventional DL Techniques for 6G SAGIN Intelligence

Over the last decade, convolutional neural networks (CNNs) have proven effective for signal classification, while deep neural networks (DNNs) excel in channel estimation and signal detection. DL methods optimize MIMO downlink beamforming using CSI and RSS, balancing performance and computational complexity, even under imperfect CSI and multi-cell scenarios. Autoencoders reduce manual inference by learning transmitter and receiver functions [93]. Given the complexity of full end-to-end physical layer design, DL is often applied to individual functions, considering power, cost, and size constraints. Simulations use realistic channel models with noise and multipath, but Monte Carlo-style evaluations require high-performance computing to limit runtime [86].

In vehicular networks, training accounts for distance, environment, speed, and weather, ensuring adaptations in one scenario do not degrade performance in others [94]. DRL techniques optimize the medium access control layer in 6G, while federated echo state networks predict user locations and orientations, enabling base stations to enhance VR QoS [59]. Conventional duplexing and interference-cancellation methods face limitations in multi-cell integration [72]. LLMs accelerate learning, provide richer prior knowledge, and improve base station (BS) deployment in dense urban networks [95]. Future research should explore LLMs for MAC optimization, resource allocation, traffic prediction, massive machine-type communications, FL-based mobility management, and security in 6G IoT environments [27,96].

### 4.2. Multimodal Sensor Data Processing in Vehicular Networks Using LLMs

LLMs can predict security requirements and allocate resources in virtual infrastructures, enhancing inferences and services through sensor fusion. Context-aware systems use LLMs to maximize application safety while minimizing SAGIN node interactions, relying on historical data and feedback rather than strict rules. They support context modeling and device control in intelligent networks. Real-time UAV applications illustrate the complexity of communication and control, where state information guides power, trajectory, and actions [30]. LLMs also enable opportunistic data transfer in vehicular networks, allowing vehicles to act as mobile sensors and support crowdsensing services, such as distributed high-definition map generation [97].

Even with CubeSats and UAVs, data transfer in vehicular networks remains challenging due to environmental effects on channel dynamics and frequent link losses in low-connectivity regions [98]. High mobility and variable line-of-sight conditions require robust communication techniques, such as opportunistic data transfer and multi-connectivity. LLMs can predict data rates to dynamically select network interfaces and optimize transmission schedules, and application-specific delay tolerance helps avoid resource-intensive transmissions [99]. Supporting LLM-driven solutions requires adapting software practices, as preloading models on user equipment is impractical due to storage limits. The ITU recommendation Y.3172 provides a framework for integrating ML into future networks. A digital twin of the network enables the safe exploration of LLM-enabled actions for effective implementation in complex 6G environments [100].

LLMs process inputs from roadside cameras, LiDAR sensors, and vehicle queries to interpret complex traffic scenarios and prioritize multimodal sensor data transmission. High-priority safety information is sent over dedicated channels, while non-critical data is compressed to ensure minimal latency for critical content. Effective semantic alignment across modalities, including human–machine interaction, allows the simultaneous interpretation of eye-tracking signals, voice commands, and text instructions for task-oriented transmission [101]. Techniques such as contrastive language-image pre-training create a unified embedding space for visual and textual data, improving communication. Dynamic resource allocation based on semantic importance is essential in bandwidth-limited environments [102].

LLM-generated semantic guidance is integrated into the semantic encoder to enhance task-oriented transmission. The encoder adjusts encoding fidelity according to semantic relevance, available bandwidth, and channel conditions as critical data is encoded with high fidelity, while less relevant segments are coarsely encoded or compressed to reduce transmission costs without performance loss [103]. On vehicular edge servers, the semantic decoder reconstructs content using received data and local context, adapting fidelity to channel conditions and synthesizing content for specific requests [96]. LLMs enable selective compression of redundant sensor data, preserving high-fidelity details for critical content, supporting realistic holographic presence and intelligent understanding while minimizing bandwidth use. Edge infrastructure in connected and autonomous vehicles provides global environmental awareness to mitigate occlusions [104]. Attention heatmaps differentiate critical from non-critical regions for adaptive bandwidth allocation, allowing task-oriented content generation with minimal transmission overhead where critical features are transmitted to vehicles, while generative models reconstruct or synthesize content [104].

### 4.3. LLMs for Visual Reasoning Tasks

Enhancing multilingual visual reasoning requires cross-modal understanding and semantic alignment. Contrastive learning aligns vision and language, extending to audio streams and voice commands, improving cross-modal feature extraction and zero-shot performance. Attention maps differentiate semantically critical from secondary regions based on text prompts, generating spatially oriented heatmaps [105]. Reconstruction fidelity is assessed using metrics like peak signal-to-noise ratio (SNR) and structural similarity index, while Fréchet inception distance and learned perceptual image patch similarity evaluate semantic preservation. Task metrics such as classification accuracy and mean average precision measure the utility of transmitted content for tasks like visual question answering and object detection. A case study illustrates LLM-enhanced semantic communications for AR and VR, where vehicles query urban elements [106].

Traditional image transmission struggles with region-specific queries, whereas LLM-based modules identify critical areas to optimize bandwidth. The architecture uses a high-fidelity encoder for key regions and a lightweight encoder for secondary regions, with a cross-attention module dynamically allocating resources based on query complexity [107]. Experiments on the *VGPhraseCut* dataset demonstrate effectiveness, using weighted mean square error loss to distinguish mask regions. The LLM allocates higher bandwidth to critical areas, improving semantic preservation. Semantic features are transmitted over noisy channels and reconstructed via a diffusion model [108]. Compression reduces overhead, and the LLM-based module preserves Gaussian-distributed features. Adaptive SNR-based estimation and distribution-matching strategies ensure robust performance under time-varying conditions [109].

The training objective optimizes LLM reconstruction loss for semantic recovery and uses a Kullback–Leibler (KL) divergence-based guidance loss for distribution alignment, ensuring fidelity under bandwidth constraints. This enables immersive content generation and adaptive synthesis guided by specific prompts [110]. Frameworks integrating LLMs leverage pre-trained models for context-aware understanding and diffusion-based generative decoding. Case studies in visual question answering for AR and VR and diffusion-driven image generation demonstrate improved reconstruction quality and semantic preservation. Mobile LLM agents with 0–10 billion parameters handle real-time tasks on in-vehicle edge servers, while edge LLM agents with over 10 billion parameters provide uninterrupted support for complex driving tasks [111].

Due to limited in-vehicle server capacity, offloading complex tasks to edge servers is necessary for long-term interactions. LLM agents, organized into perception, grounding, and alignment modules, collaborate to perform interactive tasks. A model caching algorithm enhances contextual model utilization and reduces network costs [112]. These agents follow user instructions, perceive their environment, and make human-comparable decisions, adapting to dynamic conditions and processing multimodal inputs. Pre-training on large datasets enables LLMs to perform diverse tasks, leveraging memory and reasoning for complex decision-making and control [113].

### 4.4. Conventional Deep Learning and LLMs

Unlike DRL agents, LLM-trained agents assume specific roles to execute task-oriented instructions, such as assisting in design, planning, and execution. Textual instructions alone are insufficient for comprehensive environmental perception [114]. LLM agents are enhanced with multi-sensory capabilities, processing vision, audio, tactile feedback, gestures, diverse sensors, and 3D maps to generate detailed environmental descriptions, improving autonomous navigation and accessibility. Modality encoders unify these inputs into a shared textual embedding space, enabling cross-modal reasoning. Mobile edge LLM agents operate within a collaborative edge–cloud framework, using compact local models (0–10 billion parameters) downloaded from edge servers [115]. Hence, historical context improves situational awareness, allowing real-time responses in complex tasks. Edge LLM agents with larger models comprising over 10 billion parameters leverage long-term memory and reasoning, while inter-agent communication allows mobile agents to offload complex processing [116]. Integrated sensing and communication (ISAC) combines sensory inputs to enhance perception modules, enabling coherent interpretation of user inputs, including eye-tracking and motion capture, with short-term memory supporting contextual understanding [117].

Audio and video inputs provide richer environmental information than text and are processed through cascaded models to enhance situational awareness. The continuous interaction between mobile and edge LLM agents enables offloading, feedback, and self-reflection, especially in noisy or bandwidth-limited channels. ISAC allows mobile agents to perform radar sensing while transmitting results, improving adaptability in dynamic environments [118]. Digital twins on edge servers support retrieval-augmented generation (RAG) and real-time optimization by continuously updating environmental data. Mobile agents use short-term memory via in-context learning, while edge agents access long-term memory stored on servers. RAG enhances consistency and performance by incorporating historical and knowledge-based information [119]. Edge LLM reasoning employs step-by-step strategies, including chain-of-thought and its self-consistent variant, supported by hierarchical structures such as tree-of-thought and graph-of-thoughts, enabling accurate problem solving with higher computational cost. Verification, reflection, and inter-agent communication ensure correctness, cross-validation, and task-oriented decision refinement [120,121].

### 4.5. Fine-Tuning Pre-Trained LLMs for Domain-Specific SAGIN Applications

In autonomous driving, fine-tuning pre-trained LLMs on domain-specific datasets such as *BDD100K* aligns model outputs with user instructions, enabling safe and context-aware responses. Inter-agent communication allows multiple vehicles to share data and computational resources, enhancing LLM performance. Mobile LLM agents leverage this learning to execute complex tasks and interact with virtual applications based on feedback from edge LLM agents [122]. They adapt to unfamiliar driving conditions, performing vehicle operations such as driving and braking while responding to dynamic road and weather changes. This autonomy is more complex than semi-automatic systems, which primarily coordinate with humans or other agents [123].

In a collaborative split-learning edge–cloud framework, mobile LLM agents comprise perception, local reasoning, and alignment modules, while edge LLM agents handle global reasoning and planning. Mobile agents use lightweight local models for low-latency, context-aware execution, whereas edge agents perform step-by-step reasoning with access to long-term memory and historical data [124]. The perception module gathers multimodal environmental data through image analysis and text understanding, enabling situational awareness. Combined with task-oriented communication and collaborative processing, this architecture ensures efficient and adaptive operation in dynamic real-world scenarios [125].

Running large LLMs on mobile devices or centralized cloud infrastructure introduces latency due to bandwidth constraints, limited computation, and user mobility. Edge computing mitigates latency by deploying smaller models closer to users, though often at the cost of reduced output quality. Smaller LLMs handle time-sensitive tasks at the edge, while larger models execute complex reasoning in the cloud, enabling intelligent task offloading based on user needs and prior interactions to balance latency and service quality [126]. Multi-agent systems further enhance performance through distributed intelligence for data retrieval and collaborative planning, including qubit-based inputs from quantum-enhanced sensing. Advanced interface tools predict user intent and dynamically offload tasks between edge and cloud to optimize responsiveness and fidelity [127].

In-context learning enables LLMs to follow human instructions and adapt from prior examples without retraining. Edge–cloud collaboration combined with quantum communication provides low-latency, high-fidelity links for real-time AI processing. Unlike centralized LLMs, small language models (SLMs) with under one billion parameters execute complex tasks directly on mobile and edge devices, reducing energy consumption and improving responsiveness [128]. Quantum-assisted edge processing further improves accuracy and secure low-latency communication, supporting applications such as real-time language translation, real-time transcription, generative image editing, and personalized content management [129]. Deploying LLMs at the network edge alleviates bandwidth and delay constraints, enabling low-latency services such as real-time language translation in 6G networks [130].

Quantum communication channels enhance reliability and security through high-fidelity, low-latency information exchange [131]. Beyond inference, LLMs support wireless network deployment by optimizing base-station placement and orientation under dynamic channels and urban interference [105]. Due to limited context windows, perception and actuation in mobile and edge LLM agents are treated as zero-shot inference tasks and evaluated using multimodal perception fidelity and task success. Edge LLM agents guide mobile agents using historical reasoning, but memory and model-size constraints prevent concurrent processing of all models [132]. Efficient service delivery therefore requires scheduling LLMs for reasoning and planning to minimize accuracy degradation, model-switching overhead, inference latency, and cloud computation costs [133].

### 4.6. Key Findings

Table 5 summarizes recent research on integrating DL, LLMs, and semantic-aware approaches in 6G networks. It compares the proposed methodologies and research gaps across physical-layer optimization, medium access control, UAV and vehicular network management, and semantic-guided communications. The reviewed works address channel estimation and beamforming, LLM-assisted resource allocation and mobility management, semantic encoding and decoding for task-oriented transmission, and multimodal fusion for connected autonomous vehicles. Identified challenges include high computational complexity, limited real-time adaptability under dynamic conditions, poor generalization across heterogeneous environments, storage and bandwidth constraints, and maintaining semantic fidelity in latency-critical scenarios. Overall, the analysis highlights the potential of LLMs to enable intelligent, context-aware, and resource-efficient communications, while underscoring key challenges for practical deployment in 6G vehicular networks.

## 5. Integration of LLMs in IMT-2030 for 6G Communications

### 5.1. AI-Native Vision in IMT-2030 and Its Alignment with LLM-Centric Network Intelligence

The ITU IMT-2030 vision defines 6G networks as being AI-native, where AI capabilities are integrated into the network architecture. In this context, LLMs serve a crucial role, functioning as reasoning engines and intent interpreters throughout the entire 6G architecture [134]. In vehicular communications supported by SAGINs:

- New man–machine interfaces through multiple local devices acting in unison, enabling intuitive access via gestures rather than typing;
- Ubiquitous and distributed computing, integrating multiple local devices with cloud resources for enhanced performance;
- Multi-sensory data fusion to generate immersive multi-verse maps and mixed-reality experiences;
- Precision sensing and actuation to monitor and control the physical environment;
- Extremely low-power or battery-less devices, powered by the network itself;
- End devices evolving into networks or subnetworks, such as machine-area networks or robot-area networks, connecting controllers, actuators, and sensors;
- Devices operating in sub-terahertz spectrum bands to act as active network nodes, enabling standalone or self-organizing networks.

Digital twin networks (DTNs) are crucial for the LLM-driven 6G networks, enabling simulations that generate synthetic data for LLM training, as well as for assessing networks before deployment. DTNs fully emulate 6G networks for the creation and optimization of LLM-enabled models. The ITU envisions a significant link between physical and digital twin networks, supporting the real-time verification, simulation, and management of SAGINs [135]. DTNs enhance SAGINs as a sensing network, providing accurate measurements of object distance, angle, velocity, and environmental factors through radio frequency analysis [136]. The ISAC framework specifies service requirements for object detection and monitoring. Accurate ISAC evaluations rely on deterministic, physics-based channel modeling to represent correlations among base stations and devices [137]. Additionally, the 3GPP Release 19 focuses on channel modeling for ISAC, employing ray-tracing models for improved performance and realistic data generation. As sensor data volumes surpass traditional user-generated data, by processing this data at the edge, valuable insights are extracted while reducing energy consumption, transforming raw data into actionable knowledge and predictive insights [138].

Table 6 summarizes recent advances in LLM-based semantic communication, AR, VR applications, and mobile edge–cloud frameworks in 6G networks. The table outlines the proposed works, methodologies, and research gaps, highlighting how LLMs are used to enhance cross-modal perception, semantic encoding, and collaborative decision-making across mobile, edge, and cloud layers. Solution approaches include attention-guided bandwidth allocation, diffusion-based denoising, multi-sensory environment perception, ISAC, RAG, and hierarchical reasoning. The identified challenges involve real-time adaptability under bandwidth constraints, computational and memory overheads, modality fusion complexity, mobile edge coordination, and ensuring semantic fidelity in autonomous systems. This analysis emphasizes the potential of LLM-enabled agents for intelligent, context-aware, and collaborative task execution.

### 5.2. Architectural Integration of LLMs in SAGINs

LLMs can interpret operator or application intents such as maximizing edge throughput for AR users in region $R_{1}$ and translating them into network control commands. Multiple LLM agents cooperate to configure the radio access network, core, and edge, ensuring adaptability and optimization. Network sensor T(t), policies P, and knowledge bases K are input to LLMs through RAG, the effective prompt expressed as $\mathrm{Prompt}\left(t\right)=f\left(T(t),P,K\right)$ ensuring context-aware response [139]. To leverage semantics from MLLMs, the semantic encoder manages compression fidelity and allocates higher bandwidth to important content. The decoder reconstructs information based on the received semantic features, guided by task prompts and local context. MLLMs process the raw data by considering task requirements and channel conditions, resulting in efficient semantic representations [140].

A resource-adaptive semantic decoder uses signals like eye tracking and AR, VR, and XR queries, which are sent to the edge server. The edge MLLM processes these requests along with multimodal sensory inputs to determine user intent and contextual scenes, analyzing high-dimensional data such as 3D point clouds and video streams to extract relevant semantic information. For example, in autonomous driving, it provides insights into occluded or blind spots. Prompt engineering and in-context learning enable the task-specific conditioning of the pre-trained MLLM [141]. The intent inferred from attention heatmaps or binary masks highlights the importance of different data segments, prioritizing critical content while efficiently managing wireless resources. Extremely high per-user data rates in the Gbps range, ultra-low latencies (<1 ms), and holographic communications with multi-view cameras push data rates into the Tbps range [29].

**Figure 4:**
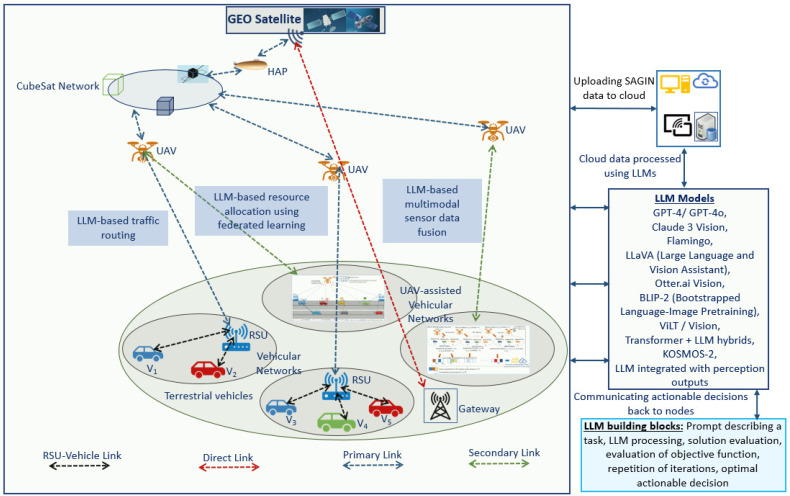
General architecture of integration of LLMs in SAGIN and UAV-assisted 6G vehicular communications.

LLMs provide semantic intelligence, user intent interpretation, and orchestration capabilities across cloud and edge devices in 6G SAGIN environments. In this context, extreme capacity xhaul refers to high-speed, fixed point-to-point links that support large data rates for aggregating information from numerous users while enabling access to computing resources in the cloud or at the edge [142]. Enhanced hotspots provide high-rate downlinks to multiple users over short coverage areas, supporting applications such as high-definition video streaming and short-range vehicle-to-vehicle communications [30]. Figure 4 illustrates a conceptual architecture for integrating LLMs in SAGIN and UAV-assisted 6G vehicular networks. The space layer consists of GEO satellites and CubeSat networks providing wide-area connectivity and backhaul support. The LEO layer includes HAPs and UAVs acting as relays, edge nodes, and sensing platforms. The ground layer comprises terrestrial vehicles, RSUs, gateways, and vehicular networks.

In 6G networks, LLMs can be deployed in the cloud, while smaller LLM variants such as SLMs can be deployed at the edge to process data collected from the SAGIN components. The deployed models, including GPT-4/GPT-4o, Claude 3 Vision (Claude 3.5 Sonnet), Audio Flamingo 3, LLaVA (LLaVA-NeXT v1.6), BLIP-2-FlanT5, ViLT, KOSMOS-2, and other vision-language and transformer-based hybrids, enable reasoning over multimodal data such as images, video, sensor streams, and network states. These models support potential applications including intelligent traffic routing, multimodal sensor data fusion, and adaptive resource allocation using FL across UAVs, RSUs, and vehicles. The communication infrastructure comprises multiple link types: primary links for core data exchange, secondary links for auxiliary connectivity, direct links for low-latency interactions, and RSU-vehicle links for vehicular communication. Based on perception-based inputs and network context, LLMs generate actionable decisions related to routing, resource scheduling, and coordination, which are communicated back to UAVs, vehicles, and other network nodes. The architecture in Figure 4 demonstrates a possible scenario where LLMs serve as an intelligent control and decision-making layer, enhancing scalability, autonomy, and efficiency in SAGIN enabled UAV-assisted 6G vehicular networks.

### 5.3. Edge-Intelligence Pipeline and Key Performance Indicators (KPI)

To address deployment challenges such as latency, limited resources, and semantic fidelity degradation, we propose a concise reference pipeline for quantum-enhanced edge intelligence in immersive SAGIN communications. The pipeline consists of five key components:

1. Perception and Semantic Encoding: Lightweight SLMs or compressed multimodal encoders operate on-device or at near-edge nodes to extract semantic representations from sensory data under strict latency and energy constraints.
2. Edge-Level Reasoning and Adaptation: Distilled or fine-tuned LLMs deployed at edge servers, UAVs, or HAPs support time-sensitive inference and task planning.
3. Knowledge Augmentation and Caching: Edge–cloud collaboration leverages RAG, semantic caching, and digital twin synchronization to optimize computation and maintain consistency across distributed nodes.
4. Cloud-Level Training and Global Intelligence: Centralized or distributed cloud infrastructures handle full-scale LLM training and global policy optimization, potentially accelerated by quantum computing.
5. Secure Coordination and Optimization: A quantum-enhanced control plane enables secure model dissemination, trusted coordination, and efficient optimization across SAGIN nodes.

### 5.4. Pipeline to Key Performance Indicator (KPI) Mapping and Performance Indicators

Each functional block in the reference pipeline is mapped to key performance indicators (KPIs) to identify deployment bottlenecks. Perception, semantic encoding, and edge-level reasoning are primarily constrained by end-to-end latency, practically targeting sub-10 ms for control applications, energy consumption, and local computational capacity. Mechanisms such as edge–cloud offloading, resource-aware RAG, and semantic caching improve bandwidth efficiency and reduce inference latency. Digital twin synchronization and distributed knowledge management affect semantic fidelity and service reliability across heterogeneous edge nodes. Cloud intelligence is evaluated in terms of scalability, training efficiency, convergence time, and resource utilization. Quantum-enhanced coordination further improves security, synchronization reliability, and optimization efficiency in dynamic large-scale networks. Overall, this reference pipeline and its KPI mapping provide a measurable framework for analyzing architectural trade-offs and guiding the design of quantum-enhanced edge intelligence systems for IMT-2030.

A hybrid quantum–classical architecture envisions LLMs functioning as reasoning and orchestration agents for both classical and quantum systems. This involves utilizing quantum computing for model training or specialized subroutines, quantum communication methods such as Quantum Key Distribution, entanglement distribution, and teleportation for secure connectivity, and multipartite entanglement for multi-node quantum protocols and distributed quantum sensing [143]. Vehicular communications are evolving to support bandwidth-intensive applications such as onboard video surveillance, broadband passenger connectivity, and remote controlled driving operations [144]. This includes using quantum computing for model training and various quantum communication methods for secure connections. Quantum compute nodes will involve both noisy intermediate-scale quantum (NISQ) and error-corrected processors, enabling quantum-enhanced tasks such as feature extraction and intent-based decision-making. The quantum communication layer is expected to utilize networks for entanglement distribution and secure end-to-end links, with quantum state summaries providing data for utilization by LLMs [25]. The Table 7 presents a comparative overview of LLM and multimodal model-driven approaches in SAGIN research. It summarizes recent developments in semantic communication, AR and VR applications, and mobile-edge-cloud architectures for 6G networks, detailing the proposed methods, system designs, and identified research challenges. The table highlights how LLMs and multimodal models support cross-modal perception, semantic representation, and collaborative intelligence across mobile, edge, and cloud layers, underscoring the usability of LLM-enabled agents for intelligent, context-aware, and cooperative decision-making in SAGINs.

### 5.5. Representing Quantum State and Fidelity Information Through Structured LLM Prompts

LLMs have evolved from n-gram models to transformer architectures, such as bidirectional encoder representations from transformers (BERT), robustly optimized BERT pre-training approach (RoBERTa), and generative pre-trained transformer (GPT)-3, enabling support for more complex natural language processing tasks and hybrid modalities like images and text. While LLMs in SAGIN-assisted vehicular networks are trained on general sensor data for broad applicability, this limits performance in specialized driving scenarios due to a lack of domain-specific data and the potential for generating inaccurate responses, known as hallucination [145]. Current benchmark open-source LLM architectures such as LLama, DeepSeek, Qwen, Phi, and Gemma using Hugging Face and LM Studio cannot process quantum states directly. Furthermore, to deploy intelligent autonomous AI agents using cutting-edge frameworks such as AutoGen, OpenAI Agents SDK, LangGraph, n8n, and MCP need summaries from classical data as grounding context:

(1) $$G\left(t\right)≜\left\{\;{\hat{\rho }}_{i}\left(t\right),\;F_{i}\left(t\right),\;{\mathrm{KPI}}_{j}\left(t\right),\;{\mathrm{config}}_{k}\left(t\right)\;\right\}$$

where ${\hat{\rho }}_{i}$ is an estimated density matrix for link *i* classical numeric summary, and $F_{i}\left(t\right)$ is the fidelity of the entangled pair in the quantum teleportation channel. These classical descriptors are appended to the RAG prompt [146]:

(2) $$\mathrm{Prompt}\;=\;\mathrm{RAG}\left(\mathrm{operator}\;\mathrm{intent},\;G(t),\;K\right),$$

where K is the model knowledge base consisting of manuals, topology, previously learned failure modes. To measure fidelity and entanglement metrics for LLM-assisted quantum channel optimization for a target pure Bell state ψ and an actual mixed state ρ, the fidelity is defined as

(3) F(ρ,ψ)=ψρψ

Teleportation or entanglement swapping is useful when the fidelity exceeds the classical threshold. For single-qubit teleportation, the classical fidelity bound is $F_{\mathrm{classical}}=2/3$; thus we require F>2/3 to realize the benefits of quantum teleportation. Multipartite entanglement may be characterized using measures such as the *n*-tangle $\tau_{n}$ for small *n*. For a 3-qubit pure state ψ, the 3-tangle $\tau_{3}$ are computed and provide a scalar measure of tripartite entanglement. Because LLMs require classical grounding of quantum state and network sensor, define

$$C\left(t\right)\;=\;\mathrm{RAG}(\underset{\mathrm{Multimodal}\;\mathrm{data}}{\underset{︸}{\left\{\mathrm{sensor}\;T(t)\right\}}},\;\underset{\mathrm{Estimated}\;\mathrm{density}\;\mathrm{matrices}}{\underset{︸}{\{{\hat{\rho }}_{m,n}\left(t\right)\},}}\;\underset{\mathrm{Fidelities}}{\underset{︸}{\left\{F_{m,n}\left(t\right)\right\}}},\;K),$$

where ${\hat{\rho }}_{m,n}\left(t\right)$ denotes a classical estimate for the two-node density matrix and K is a domain knowledge base [147].

When discussing quantum teleportation fidelity thresholds (e.g., $F>\frac{2}{3}$) and fragile quantum links in SAGIN, evaluating their practical feasibility is crucial. Quantum links are affected by channel noise, pointing and tracking errors, Doppler shifts from high-mobility platforms, and the complexity of quantum repeaters and entanglement sources. LEO satellites and HAPs can support quantum key distribution over line-of-sight optical links, though long-distance entanglement distribution faces challenges such as decoherence and multipartite entanglement. UAVs can assist in short-range entanglement swapping or quantum key distribution in controlled conditions, aided by trajectory prediction and beamforming techniques. Ground stations provide stable anchors for quantum communication, enhancing stability for both fidelity computation and entanglement distribution [148]. Considering these practical factors alongside fidelity thresholds allows to map quantum primitives to SAGIN links while addressing reliability, latency, and hardware constraints.

### 5.6. LLM-Orchestrated Entanglement Routing in SAGIN

An LLM translates a high-level intent, such as *“Establish a high-fidelity entangled link between edge site A and edge site D with minimal classical control plane overhead; prefer satellite uplink if terrestrial links fall below fidelity 0.85.”* into a sequence of actions: Query G(t) for current link fidelities $F_{\mathrm{A-B}}$, $F_{\mathrm{B-C}}$, $F_{\mathrm{C-D}}$. If the direct terrestrial path $\min(F_{\mathrm{A-B}},F_{\mathrm{B-C}},F_{\mathrm{C-D}})\geq 0.85$, request entanglement swapping at nodes *B* and *C*. Otherwise, propose a satellite-mediated entanglement distribution and schedule quantum repeater resources [40]. For the entanglement swapping chain of repeaters for three nodes *A*-*B*-*C*, after Bell-state measurements at *B* swapping, the end-to-end fidelity $F_{A,C}$ is a function of local fidelity and swap errors. For idealized depolarizing channels with fidelity $F_{A,B},\;F_{B,C}$ and swap map S, a simplified composition is

(4) $$\rho_{A,C}\approx S\left(\rho_{A,B},\rho_{B,C}\right),\;F_{A,C}\approx f_{\mathrm{swap}}\left(F_{A,B},F_{B,C}\right).$$

where $f_{\mathrm{swap}}$ is typically increasing in its arguments but lower than the minimum fidelity. Multipartite entanglement measures for an *n*-qubit state ρ shared among nodes; multipartite entanglement is measured by *n*-tuple $\tau_{n}\left(\rho \right)$ generalized concurrence C(ρ). For pure ψ, the *n*-tuple is $\tau_{n}\left(\psi \right)=\left(\psi \right)$ and operational use requires $\tau_{n}$ above protocol-specific thresholds [149].

### 5.7. RAG Parameterization Template and Evaluation Checklist

To implement RAG for time-sensitive edge intelligence in SAGIN, we propose a checklist that guides parameter selection and system design:

- **Inputs:** Multimodal observations, including textual reports, sensor measurements, visual imagery, and telemetry streams, collected from heterogeneous sources such as edge devices, UAV platforms, and satellite systems. These inputs reflect both real-time and near-real-time environmental and operational states.
- **Metadata/Time-Stamps:** Each input is enriched with auxiliary metadata, including precise time-stamps, geospatial location, originating node or platform, and semantic annotations. This metadata enables temporal-aware retrieval, provenance tracking, and context-sensitive reasoning across distributed nodes.
- **Retrieval Strategy:**
  – *Temporal prioritization:* Assign higher retrieval weights to recent observations and temporally relevant data to ensure responsiveness to rapidly evolving scenarios.
  – *Node-aware caching:* Cached or locally stored knowledge at the edge to reduce communication overhead and minimize retrieval latency under constrained network conditions.
  – *Similarity metrics:* Employ embedding-based similarity measures derived from multimodal LLMs and SLMs to perform semantic matching across heterogeneous data modalities.
  – *Fallback mechanisms:* Expand retrieval scope to broader or historical knowledge sources when recent or local data is sparse, missing, or unreliable.
- **Sources of Error:**
  – Missing, incomplete, or outdated information in local caches.
  – Temporal misalignment or synchronization errors across distributed sensing and computing nodes.
  – Retrieval latency exceeding real-time or mission-critical thresholds.
  – Semantic inconsistency due to partial retrieval can be identified by answering the following questions:
    * *Latency compliance:* Does the retrieval process satisfy end-to-end time-sensitive and real-time operational requirements?
    * *Semantic fidelity:* Are the retrieved entries contextually accurate and consistent with current observations and mission intent?
    * *Temporal accuracy:* Are time-stamped inputs correctly ranked and prioritized, particularly for recent or fast-changing events?
    * *Cache efficiency:* What proportion of retrievals are served from local edge caches versus remote nodes or cloud resources?
    * *Robustness to failure modes:* How does the system degrade under missing, delayed, or noisy data conditions?
    * *Energy and computation overhead:* Are RAG-related operations feasible within the power, memory, and compute constraints of edge devices?

These questions provide a structured methodology for RAG parameter selection, temporal-aware recall, and systematic evaluation in immersive SAGIN edge intelligence scenarios.

Note, traditional single-modality sensing has limitations in accuracy and adaptability, and its integration in SAGIN communication systems increases latency. To address these issues, a semantic-driven integrated multimodal sensing and communication framework has been developed in [150]. The proposed solution combines radar and image modalities through a multimodal semantic fusion network that uses cross-attention to generate semantic representations. LLM-based semantic encoder maps these semantics and communication parameters into a unified latent space for efficient encoding. Task-specific decoding is handled by a sensing semantic decoder that employs multiple heads and a multi-task learning strategy to enable diverse sensing services. Experimental results indicate that the proposed method improves sensing accuracy and supports heterogeneous multi-task requirements, demonstrating the benefits of integrated multimodal sensing and communication in edge-intelligent SAGIN systems [150].

### 5.8. Hybrid Quantum–Classical Training and Inference for Next-Generation LLM Models

A commonly proposed hybrid scheme uses parameterized quantum circuits as differentiable modules that provide quantum feature embeddings to a classical mapping. For data vector *x* and parameterized quantum circuit parameters θ

(5) $$\phi (x;\theta )\;\mathrm{and}\;m\left(x;\theta \right)={\langle O\rangle }_{\phi (x;\theta )}$$

An LLM loss function is augmented with a quantum regularizer as follows:

(6) $$L\left(\omega ,\theta \right)\;=\;L_{\mathrm{LLM}}\left(\omega \right)\;+\;\lambda \;L_{Q}\left(\theta ;\omega \right),$$

where ω denotes the classical model parameters, $L_{\mathrm{LLM}}$ is the cross-entropy token prediction loss, and $L_{Q}$ is a quantum loss such as expectation mismatch, kernel alignment, or downstream classification computed from measurements m(x;θ). Training is conducted with hybrid optimizers where quantum gradients are estimated via parameter-shift rules, and classical gradients are computed using backpropagation [151]. Let *t* denote continuous time or discrete time-steps indexed by *k*. V be the set of vehicles, U the set of UAVs acting as relay nodes, and B the set of ground base stations. For vehicle i∈V at time *t* we have: position $p_{i}\left(t\right)\in R^{3}$, velocity $v_{i}\left(t\right)$, and data demand $D_{i}\left(t\right)$. For UAV u∈U we have: position $q_{u}\left(t\right)$, trajectory control $a_{u}\left(t\right)$, and available quantum buffer or repeater resources $R_{u}\left(t\right)$. For the classical channel rate between node *m* and *n*, we have $C_{m,n}\left(t\right)$ (bits/s). The quantum link fidelity between *m* and *n* is $F_{m,n}\left(t\right)\in \left[0,1\right]$. LLMs parameterized by θ are used for orchestration and intent translation; prompt and context at time *t* is C(t) [152].

LLMs are used as probabilistic policy translators. The LLMs act as a conditional probability model that maps a natural language intent and context into an action sequence or policy π to control commands and parameter updates. For token sequence $y=(y_{1},\ldots ,y_{T})$ conditioned on input prompt *x* and context C(t),

(7) $$P_{\theta }\left(y|x,C(t)\right)\;=\;\prod_{r=1}^{T}P_{\theta }\left(y_{r}|y_{<r},x,C\left(t\right)\right).$$

A decoded sequence $\hat{y}$ is based on a policy $\pi_{\hat{y}}$ that maps to low-level network actions A={a} via a translator and query vector *g*:

(8) $$\pi_{\hat{y}}\;=\;g\left(\hat{y}\right),\;a\left(t\right)=\pi_{\hat{y}}\left(t\right).$$

Losses and fine-tuning are conducted as the LLM is updated with performance feedback from the network throughput, fidelity, and latency. We optimize a reward R(τ) over execution traces τ using policy-gradient style updates:

(9) $$L_{\mathrm{LLM}}\left(\theta \right)\;=\;-E_{\hat{y}∼P_{\theta }\left(\cdot |x,C\right)}\left[R(\tau \left(\hat{y}\right))\right]\;.$$

A mixed hybrid loss combining supervised token loss and task reward is

(10) $$L\left(\theta \right)\;=\;L_{\mathrm{CE}}\left(\theta \right)\;-\;\lambda \;E_{\hat{y}∼P_{\theta }}\left[R\left(\tau \left(\hat{y}\right)\right)\right],$$

where $L_{\mathrm{CE}}$ is the cross-entropy on supervised examples, and λ≥0 trades off the task reward. In classical wireless channel and UAV mobility, the UAV-ground path-loss and channel power gain model is

(11) $$h_{mn}\left(t\right)\;=\;L_{0}\;d_{mn}{\left(t\right)}^{-\alpha }\;\chi_{mn}\left(t\right),$$

where $d_{mn}\left(t\right)=∥x_{m}\left(t\right)-x_{n}\left(t\right)∥$ is the distance, α is the path-loss exponent, $L_{0}$ is a reference gain, and $\chi_{mn}\left(t\right)$ models shadowing and fading such as Rician with the *K*-factor depending on elevation angle for UAV links. The instantaneous classical link capacity under bandwidth *B* and transmit power *P* is approximately

(12) $$C_{m,n}\left(t\right)\;=\;B\log_{2}\;\left(1+\frac{P\;|h_{mn}{\left(t\right)|}^{2}}{N_{0}B+I_{mn}\left(t\right)}\right),$$

where $N_{0}$ is noise PSD and $I_{mn}\left(t\right)$ is interference [153]. The UAV motion dynamics for UAV *u* is

(13) $$\begin{array}{cc} q_{u}\left(k+1\right) & =q_{u}\left(k\right)+\Delta t\;v_{u}\left(k\right)+\frac{1}{2}\Delta t^{2}\;a_{u}\left(k\right), \end{array}$$

(14) $$\begin{array}{cc} v_{u}\left(k+1\right) & =v_{u}\left(k\right)+\Delta t\;a_{u}\left(k\right), \end{array}$$

with constraints $∥a_{u}\left(k\right)∥\;\leq a_{\max}$ and speed bounds. For quantum link representation, the density matrices, fidelity, decoherence model a bipartite quantum link between nodes *m* and *n* at time *t* by estimated density operator $\rho_{m,n}\left(t\right)$. For a target pure Bell state $\Phi^{+}$, the fidelity is

(15) $$F_{m,n}\left(t\right)\;=\;\Phi^{+}\rho_{m,n}\left(t\right)\Phi^{+}.$$

Time evolution under decoherence amplitude damping or depolarizing is modeled as a quantum noise map $E_{t}$:

(16) $$\rho_{m,n}\left(t+\Delta t\right)\;=\;E_{\Delta t}\left(\rho_{m,n}\left(t\right)\right).$$

At each time step *t*, the LLM-guided joint optimization problem balances classical throughput, quantum utility, fidelity, key rate, latency, and the node’s energy consumption. Let x(t) denote classical resource allocations pertaining to power, spectrum, and scheduling. Let y(t) denote quantum resource decisions pertaining to entanglement links, and repeater assignments. The term π(t)∈Π is the the LLM generated orchestration plan. The objective is to maximize

(17) $$\begin{array}{cc} \; & \max_{x(\cdot ),\;y(\cdot ),\;\pi (\cdot )\in \Pi }\;\underset{\mathrm{classical}\;\mathrm{throughput}}{\underset{︸}{\sum_{i\in V}\;\alpha_{i}C_{i}\left[x,\pi \right]\left(t\right)}}\;+\;\underset{\mathrm{quantum}\;\mathrm{utility}}{\underset{︸}{\sum_{\left(m,n\right)}\beta_{mn}\;U_{Q}\left(F_{m,n}\left[y,\pi \right]\left(t\right)\right)}}\\ \; & \;\;\;-\;\gamma \;\mathrm{Delay}[x,y,\pi ](t)\;-\;\eta \;E[x,y,\pi ](t) \end{array}$$

subject to the following constraints:

$C_{1}$: UAV dynamics: $q_{u}\left(k+1\right)=f_{\mathrm{dyn}}\left(q_{u}\left(k\right),a_{u}\left(k\right)\right),\;\forall u,$
$C_{2}$: Link capacity constraints: $C_{m,n}\left(k\right)\leq B\log_{2}\;\left(1+{\mathrm{SINR}}_{m,n}\left(k\right)\right),$
$C_{3}$: Quantum link evolution: $\rho_{m,n}\left(k+1\right)=E_{\Delta t}\;\left(\rho_{m,n}\left(k\right);y\left(k\right)\right),$
$C_{4}$: Quantum fidelity thresholds: $F_{m,n}\left(k\right)\geq F_{\min}^{\left(q\right)}$, if quantum processing is executed,
$C_{5}$: Resource constraints: $\sum_{i}P_{i}\left(k\right)\leq P_{\max},\;\sum_{u}R_{u}\left(k\right)\leq R_{\mathrm{total}},$

where $U_{Q}\left(\cdot \right)$ denotes a quantum utility function capturing fidelity or key-rate performance (e.g., $U_{Q}\left(F\right)=\log\left(1+\kappa K\left(F\right)\right)$), and $C_{i}\left[x,\pi \right]$ represents the classical throughput of user *i* under allocation *x* and policy π [154]. The LLM actions act as constrained priors as the LLM proposes a candidate π that must satisfy safety and feasibility conditions as

(18) $$V\left(\pi ,C(t)\right)\;=\;\left\{\begin{array}{cc} 1 & \mathrm{if}\;\pi \;\mathrm{meets}\;\mathrm{fidelity},\\ 0 & \mathrm{otherwise}. \end{array}\right.$$

Thus, an action is accepted only if V(π,C(t))=1. For hybrid quantum–classical communication links, the co-design objective with hybrid quantum–classical learning is as follows: If a hybrid quantum module, modeled as a parameterized quantum circuit with parameters ϕ, augments the LLM decision features, then the model training minimizes

(19) $$\min_{\theta ,\phi }\;E\left[L_{\mathrm{task}}\left(\mathrm{Exec}(g\left({\mathrm{LLM}}_{\theta }\left(C\right)\right)),\;\phi \right)\right]\;+\;\lambda_{Q}\;R_{Q}\left(\phi \right),$$

where $L_{\mathrm{task}}$ measures network-level performance, and $R_{Q}$ regularizes the quantum module to limit circuit depth or noise-sensitivity. Gradients with respect to the variable ϕ are estimated with parameter-shift and with respect to the variable θ with policy gradients or supervised gradients. A common deterministic verifier combines

(20) $$V\left(\pi ,C\right)=H\left\{\min_{\left(m,n)\in Q(\pi \right)}F_{m,n}\geq F_{\mathrm{safe}}\;\wedge \;\mathrm{Latency}\left(\pi \right)\leq L_{\max}\right\},$$

where Q(π) are quantum links that the plan uses, and $F_{\mathrm{safe}}$ is the minimum acceptable fidelity. Here, the LLMs must also manage quantum communication session keys, schedule quantum communication windows, and choose fallback strategies when quantum communication link fidelity is low. LLMs translate the intents for quantum repeater allocation, multiplexing, and entanglement routing actions. Parameterized quantum circuits provide feature transforms used by LLMs for tasks such as anomaly detection on quantum sensor data. Because LLMs avoid actions that would degrade quantum links, every LLM-issued action must be subject to verification [155]. Let A denote the set of candidate actions generated by the LLM where we define a verifier function V:A×G→{0,1} that accepts only actions that preserve fidelity thresholds and obey policy constraints. All the indicators in a group must be simultaneously satisfied, while different groups may carry distinct requirements [156].

In recent and existing works, publicly available pre-trained LLMs have been trained on extensive datasets spanning multiple domains, including wireless communications, enabling these models to incorporate a broad knowledge base. The objective is to extract latent information embedded within LLMs to develop advanced decision-making mechanisms for wireless network deployment. The attenuation experienced by a signal as it propagates through space is primarily due to the spreading of the wavefront, which reduces signal power with distance [157]. The free-space path loss model is expressed in decibels (dB) as

(21) $$\mathrm{PL}\;\left(\mathrm{dB}\right)=20\log_{10}\left(d\right)+20\log_{10}\left(f\right)+20\log_{10}\left(\frac{4\pi }{c}\right),$$

where *d* is the distance between the transmitter and receiver in meters, *f* is the signal frequency in Hertz, and *c* is the speed of light, $3\times 10^{8}\;m/s$. Shadowing, or slow fading, occurs when obstacles such as buildings or trees block the direct path between transmitter and receiver. It is modeled as a log-normal distribution, where the received power in dBm is normally distributed around the mean path loss with a standard deviation that depends on the environment:

(22) $$P_{r}\left(\mathrm{dBm}\right)=P_{t}\left(\mathrm{dBm}\right)-PL\left(\mathrm{dB}\right)-X_{\sigma }\left(\mathrm{dB}\right),$$

where $P_{r}\;(\mathrm{dBm})$ is the received power, $P_{t}\;(\mathrm{dBm})$ is the transmitted power, PL(dB) is the path loss, and $X_{\sigma }\left(\mathrm{dB}\right)$ is a zero-mean Gaussian random variable with standard deviation σ representing shadowing effects [158]. The exploration is facilitated using the Ornstein–Uhlenbeck process as

(23) $$dX_{t}=\theta \left(\mu -X_{t}\right)dt+\sigma dW_{t},$$

where $X_{t}$ is the process value at time *t*, θ is the mean-reversion rate, μ is the mean, σ is the volatility, and $dW_{t}$ is the Wiener process increment [159]. The noise from this process is added to deterministic policy actions as

(24) $$a_{t}^{\prime }=\mu \left(s_{t}|\theta_{\mu }\right)+N_{t},$$

where $\mu (s_{t}|\theta_{\mu })$ is the deterministic policy and $N_{t}$ is the Ornstein–Uhlenbeck noise. LLMs interpret environmental characteristics and guide base station placement to maximize coverage. Instead of relying solely on analytical electromagnetic models, this approach leverages learning-based perception of the environment. This exemplifies the integration of intelligent LLM agents with SAGIN for adaptive, robust, and self-optimizing network deployment. In traffic across different open areas and in traffic jam on main roads, the objective is to adjust the 3D location and orientation of a base station to maximize coverage and signal quality for users [160]. The received signal power values are at specified user locations. The actions include the 3D location (x,y,z) and orientation $\left(\alpha_{1},\alpha_{2},\alpha_{3}\right)$ of the base station, with x,y∈[−500,500], z∈[20,120], $\alpha_{1}\in \left[-\pi ,\pi \right]$, $\alpha_{2}\in \left[-\pi /2,\pi /2\right]$, and $\alpha_{3}\in \left[-\pi ,\pi \right]$. The LLM-based models benefit from pre-trained knowledge, enabling faster adaptation and improved performance in interpreting complex instructions. The LLM-based models are limited by the sequence length they can process but provide richer semantic understanding of deployment objectives [161]. The pre-training of the LLM enables it to comprehend objectives clearly and immediately from input prompts, providing a significant advantage in prior knowledge and objective identification [162]. This formulation also ensures that the agent’s reward is directly proportional to the signal strength, facilitating targeted and efficient learning. Prompt selection is crucial for effectively leveraging LLMs, particularly when executing driving decisions in connected and autonomous vehicles [163]. To ensure the clear and concise description of the state to the LLM, it is crucial we generate prompts with the assistance of a pre-trained language model [164]. These prompts should maximize comprehension of the objective and the environment, ensuring that the LLM-based actor models rely on semantic understanding rather than only on loss functions. A pool of candidate prompts should be empirically evaluated to select the most effective prompt [165].

### 5.9. Keyword-Based Retrievals in SAGIN Data Communication Using LLMs

Additionally, in IoT deployments such as smart cities and autonomous vehicles, AI and generative AI are essential for scalable connectivity, efficient data processing, intelligent content generation, and network self-organization. LLMs advance 6G toward an AI-native paradigm supported by programmable GPU-accelerated libraries [166]. By modeling interactions among network elements, LLMs combined with GPU-accelerated simulations enable the rapid prototyping and evaluation of communication algorithms. These platforms support city-scale network simulations, optimizing transmission strategies and enabling real-time LLM training to improve spectral efficiency and communication fidelity [167]. Integrating geographic information system data further enhances simulation fidelity, producing realistic outputs such as channel impulse responses and qubit fidelity metrics. Combining LLMs with agent-based mechanisms extends their capabilities, bridging classical and quantum communication paradigms for efficient wireless network design [168]. The integration of generative AI with 6G networks represents a major advancement in wireless intelligence [169]. Quantum communication links ensure the high-fidelity transmission of model outputs, preserving AI result integrity, while generative AI enables context-aware responses and intelligent content rewriting. Dynamic task management and adaptive resource allocation based on user demands and network conditions further enhance service delivery [170].

A critical challenge is optimizing task offloading between LLMs and SLMs under latency, computational, and fidelity constraints [171]. Quantum-enhanced communication improves synchronization and fidelity verification between edge and cloud AI models, enhancing reliability and reducing errors. To address these challenges, fine-tuning and RAG are widely adopted. Fine-tuning adapts pre-trained LLMs to task-specific data while preserving general capabilities [172]. RAG integrates LLMs with external knowledge bases, dynamically retrieving relevant information to improve accuracy and reduce hallucinations. In RAG architectures, data is embedded into vector and keyword indexes to enable low-latency retrieval. During inference, the LLM identifies user intent and selects relevant knowledge to guide response generation [173]. Hybrid retrieval methods combining semantic and keyword-based approaches handle linguistic variation and ambiguity effectively. Structured knowledge segments further support accurate response generation based on both the query and retrieved context [174].

In hybrid classical–quantum communication architectures, entanglement-assisted retrieval and processing enable secure, low-latency transfer of knowledge segments between distributed LLM instances, preserving fidelity and consistency across edge and cloud deployments. The integration of RAG, fine-tuning, and quantum communication forms an effective framework for knowledge management in next-generation wireless networks [175]. Semantic retrieval captures contextual relevance, while keyword-based retrieval guarantees exact term matching but lacks semantic understanding. Hybrid retrieval combines both approaches by mapping queries into high-dimensional embeddings while ensuring precise keyword matches, improving overall relevance. Embedding models convert text and multimodal content for semantic search, enabling LLMs to generate high-fidelity responses [176].

The architecture stores queries, accurate answers, and relevant or irrelevant contextual descriptions in vector databases to support low-latency retrieval. Performance is evaluated using metrics such as faithfulness, answer relevance, context precision, and recall. Quantum-enhanced retrieval helps maintain knowledge base integrity, while multimodal data processing is essential for extracting key information from unstructured data, improving LLM performance [177]. Optimizing RAG parameters remains challenging and often relies on empirical tuning. Expanding context windows, simplifying input formats, and minimizing interface parameters improve sensor data utilization, while incorporating temporal attributes into retrieval prioritizes up-to-date information for time-sensitive applications [178].

### 5.10. Key Findings

Table 8 presents a comprehensive overview of LLM, edge, quantum, and RAG integration strategies for 6G networks. It summarizes the proposed works, methodologies, and identified gaps, emphasizing how LLMs, quantum-enhanced communication, and RAG collectively enable low-latency, high-fidelity, and AI-native 6G services. Key methodologies include edge–cloud hybrid deployments, multi-agent collaboration, GPU-accelerated digital twin simulations, quantum-assisted fidelity verification, domain-specific LLM fine-tuning, and multimodal data embeddings with temporal-aware metrics. The table also highlights critical challenges such as hybrid classical–quantum system complexity, limited training data, reasoning and semantic retrieval limitations, latency and resource allocation constraints, and the scalable integration of multimodal and temporal information.

As wireless connectivity evolves from linking people to supporting interconnected machine-type devices, it introduces diverse applications and requirements [97]. While 5G networks provide high data rates, the power consumption of many devices remains a concern, leading to the development of low-power wide-area networks for long-range, energy-efficient connectivity [179]. Industrial communication networks address stringent requirements for automation, while AI-assisted processing in vehicles demands low latency and high reliability [180].

## 6. Quantum-Enhanced Communication for Ultra-Intelligent SAGIN

Quantum communication and entanglement enhance coordination and system performance, enabling reliable task execution [143]. High-fidelity data transmission over entangled quantum links is critical for seamless autonomous driving and immersive vehicular environments [181]. Quantum-enhanced communication improves data integrity and authentication, while quantum-assisted sensing enables precise localization, supporting ultra-reliable and low-latency communication for coordinating autonomous vehicles and UAV swarms [182]. In multi-component environments, competition for radio resources poses significant challenges. Quantum communication enhances reliability and information exchange across heterogeneous and disjoint networks [183].

Cell-free network architectures reduce signaling overhead and improve reliability by allowing multiple base stations to jointly process transmissions. Network heterogeneity will further increase with the emergence of micro-operators, diverse radio access technologies, and smaller cell sizes enabled by higher frequency bands [184]. Radio-frequency wireless energy transfer requires efficient spectrum allocation and adaptive strategies to sustain energy-constrained devices [185]. Intelligent, software-defined networks enable dynamic orchestration of end-to-end applications, where real-time localization, optimization, and distributed intelligence are essential. Quantum entanglement supports reliable synchronization and high-fidelity data exchange in dynamic environments, while energy-efficient devices are critical for massive and mission-critical machine-type communications [186]. An extensive overview of quantum communication systems is provided in [187].

### 6.1. Improved Quantum Processing for Real-Time Optimization in 6G Networks

Improved quantum processing enhances decision-making, reduces latency, and maintains communication fidelity. Current transceiver designs need ultra-low power consumption, which is achieved through integrated systems and event-driven architectures. Sub-GHz bands with simple modulation schemes such as on-off keying achieves power consumption below 100 nW while ensuring high signal integrity [188]. Ambient backscatter communications minimizes costs and power needs by modulating radio frequency signals from ambient sources, while techniques such as spatial null-steering manage direct-path interference. In bi-static configurations, optimizing path loss maximizes coverage, and integrating quantum entanglement in backscatter devices enhances communication fidelity. Energy harvesting supports long-lasting, battery-free devices and ensures coexistence with legacy receivers by shifting backscattered signals to guard bands or low-interference sub-bands [189].

The integration of quantum-enhanced detection techniques improves the reliability of signal decoding under ultra-low-power conditions, ensuring secure network operations. Efficient downlink signaling recognition allows quick frame decoding and reachability, while short duty cycling reduces power consumption. Adaptive receiver blocks are essential for capturing signals in zero-energy or self-powered scenarios, adjusting to varying context and channel conditions [190]. These receivers must detect active channels across wide frequency bands, maintaining high fidelity amid strong signal interference cancellation to simultaneously support numerous devices effectively. Non-orthogonal solutions are crucial for managing massive traffic over grant-free channels, even without channel state information. Innovative random access protocols and persistent scheduling are needed to handle diverse traffic characteristics [191].

Quantum communication enhances reliability and security for these networks. Non-orthogonal multiple access (NOMA) is vital for efficient shared resource access, requiring effective user detection and data decoding to minimize collisions. Advanced receivers with multi-user detection and interference cancellation algorithms enhance performance. CSI is key to improving communication fidelity, although real-time CSI acquisition is impractical in dense networks [192]. Intelligent beamforming approaches conserve energy while maintaining performance. The presence of small cells and non-terrestrial constellations allows for better random access performance, and quantum entanglement further enhance collision resolution. Persistent scheduling and resource allocation are essential for managing heterogeneous traffic and QoS requirements [190].

Modern random access schemes effectively handle sporadic and bursty traffic, but periodic and time-sensitive applications with strict latency and jitter requirements benefit from persistent scheduling. By categorizing traffic for sporadic, periodic, or event-driven transmissions and applying various access schemes to enhance performance [193]. Entanglement-assisted synchronization provides precise timing and phase coherence across devices. Point-to-multipoint delivery within core networks is crucial for transmitting content to multiple devices while maintaining stable QoS [194]. Quantum communication principles enhance the fidelity and security of these transmissions, ensuring reliable delivery across diverse devices while conserving energy and spectrum. These applications require ultra-reliable, low-latency services that approach wired communication performance. Key performance indicators include end-to-end latencies as low as 0.1 milliseconds and block error rates (BLERs) around $10^{-9}$ [195].

### 6.2. Quantum Communications Assisted by LLMs in 6G Networks

The current adaptation for ultra-reliable low latency communication through shorter transmission time intervals is inefficient for SAGIN. Resource allocation should leverage predictable application requirements and utilize flexible, resource-efficient solutions [196]. LLMs identify traffic patterns and optimize scheduling, allowing applications to declare transmission characteristics. Resource allocation is then optimized across multi-link and heterogeneous networks, with careful consideration of time bounds and resource costs. LLMs enhance resource awareness by monitoring and predicting resource availability, improving overall efficiency [81]. Quantum communication can boost reliability and coordination, ensuring robust operations across varied networks. Additionally, creating digital twins allows for decision simulation, preventing overload while supporting resource allocation that meets stringent timing guarantees [197].

Semi-persistent scheduling and methods for cellular vehicle-to-everything and time-sensitive networking must evolve to function effectively in distributed environments. LLMs enable programmable wireless environments that adapt to the requirements of reliable transceiver design, managing collisions caused by rapidly changing network topologies and high density transmissions, such as vehicle to vehicle communication [198]. Collision-tolerant transceivers and grant-free NOMA methods separate users experiencing collisions, while full duplex operation ensures reliable reception [199]. They must be adapted to resource-constrained vehicles and uplink-dominant networks, raising challenges for two-way trust and requiring secure protocols that generate keys based on device fingerprints.

Critical advancements include ultra-massive MIMO, intelligent reflecting surfaces, and the convergence of space and terrestrial infrastructure [200]. Spectrum use will expand from sub-6 GHz to THz and visible light communications, demanding Tbps-level throughput, sub-millisecond latency, and the ability to support billions of connected devices in energy-efficient, reliable architectures [201]. End-to-end network operations will rely on intelligent orchestration, incorporating AI-driven analytics. LLMs will enable hyper-localized micro-operators and service provisioning, while network slicing allows for per-application customization. Effective monitoring and verification strategies must be implemented in both network infrastructure and end devices to ensure performance guarantees. Overall, LLMs will enhance the adaptability of networks and applications based on user profiles and contextual information [202].

### 6.3. Quantum Communication and Fidelity Metrics for Secure and Ultra-Low-Latency Links

To support heterogeneous networks, including satellite constellations, quantum communication techniques enhance security and fidelity in data transmission. Fidelity metrics will be crucial for evaluating entangled states and ensuring reliable quantum key distribution. Integrating classical and quantum networking paradigms facilitate new service classes, such as holographic communications and mission-critical applications [106]. LLMs enable interactions between management planes and third parties, optimizing management for virtual operators under policy-based control. By utilizing a service-based architecture, LLMs automate services at the edge cloud and support dynamic network customization and service composition. Service communication proxies ensure secure, low-latency inter-service communication, while LLMs enable slice-specific deployments tailored to resource requirements [203]. The decomposition of user plane functions into modular services fosters dynamic deployments and ensures end-to-end reliability. Increasing protocol diversity will address the growing scale of requests [204].

LLMs will facilitate coordination across access, backhaul, and core domains, promoting service discovery and load balancing while maintaining domain autonomy. LLMs will adapt to user-driven requirements and leverage profiles for intelligent network segment selection. Distributed agents in each segment will autonomously coordinate service deployment, meeting communication needs [205]. Quantum-enabled LLMs will ensure the integrity of quantum links while maintaining performance guarantees across diverse network segments. High-precision sensor and analytics will enable predictive orchestration, ensuring fidelity in both classical and quantum communications [86]. The growing complexity of these scenarios requires distributed management systems without a single control point. This approach will leverage sensor networks, quantum-enhanced analytics, and LLMs to meet the demands of cloud-native applications [206].

### 6.4. Challenges in UAV–Vehicle Quantum Communications Across Dynamic Environments

The realization of quantum communication between UAVs and ground vehicles introduces some challenges. Although quantum communication promises secure and ultra-low latency information exchange, its deployment on airborne and vehicular platforms remains constrained by the limited computational and energy resources available on UAV-embedded processors and vehicular edge servers. Generating, storing, transmitting, amplifying, and receiving quantum information requires both stable quantum hardware and high-fidelity control operations, which are extremely sensitive to noise, mobility-induced disturbances, and environmental fluctuations. These challenges are intensified in noisy intermediate-scale quantum (NISQ) devices, which lack full error-correction capabilities and therefore struggle to maintain coherent quantum states over extended periods [144]. The key challenges associated with UAV–vehicle QC are described below:

- **Limitations in number of usable qubits**: The exponential scaling of quantum state space means that an *n*-qubit quantum system occupies a Hilbert space of size $2^{n}$, requiring substantially greater computational and memory resources to manipulate, simulate, and transmit as *n* increases [207]. In UAV–vehicle settings, where processors have stringent constraints on power consumption, weight, and size, only a small number of qubits can realistically be supported. This restricts the complexity of quantum communication protocols that can be executed onboard and limits the feasibility of advanced quantum algorithms that require large entangled registers for optimal performance.
- **Limitations in quantum measurement** Effective QC requires accurate quantum measurements and high-quality quantum memory to store incoming photons for sufficiently long durations to preserve their encoded information. In mobile platforms such as UAVs and vehicles, vibration, thermal fluctuations, and rapid changes in orientation introduce disturbances that can alter the quantum state during measurement [187]. Even minimal inaccuracies during measurement collapse the state unpredictably, leading to significant fidelity loss. As a result, maintaining measurement precision in constantly changing airborne and roadway environments presents a major barrier to reliable quantum communication.
- **Restriction in amplification of quantum signals**: Classical communication systems often rely on signal amplification to extend communication range, but quantum signals cannot be amplified due to the no-cloning theorem. Since the exact state of a qubit cannot be copied or reconstructed, lost quantum amplitude cannot be recovered mid-transmission [187]. This constraint severely limits the distance over which quantum information can be transmitted between UAVs and vehicles. Additionally, atmospheric attenuation, scattering, and weather-induced turbulence further degrade signal strength, making long-distance quantum communication in dynamic air-to-ground channels particularly challenging.
- **Scalability of qubits**: Embedding UAVs and vehicles with multiple qubits and enabling reliable short-range connectivity between qubits is non-trivial. Physical qubits often require cryogenic cooling, electromagnetic shielding, or highly stable optical cavities, none of which are easily integrated into lightweight UAV hardware [187]. Even if small quantum processors could be embedded, limited qubit connectivity restricts the ability to perform multi-qubit operations efficiently. This leads to longer operation times, increased decoherence risk, and the reduced scalability of onboard quantum communication systems, slowing down tasks such as entanglement distribution and multi-node quantum networking.
- **Low error tolerance**: Quantum information encoded within a single photon is extremely sensitive to noise introduced by atmospheric conditions, scattering, turbulence, and hardware imperfections. While quantum error correction can theoretically protect information using multiple redundant physical qubits, implementing these schemes requires additional qubits and computational overhead, which exceeds the capabilities of UAV and vehicular platforms [208]. Consequently, quantum communication links between UAVs and vehicles suffer from decoherence and signal degradation over short distances, significantly reducing the achievable reliability of quantum transmissions.
- **Preservation of quantum states**: Preserving the fidelity of quantum states is essential for ensuring that the transmitted information remains accurate and usable at the receiver end. Quantum memories can store and process information from multiple sources simultaneously, but their stability is strongly affected by motion dynamics and environmental conditions [208]. As UAV trajectories change due to navigation adjustments, wind patterns, or altitude shifts; the reference frame for previously stored quantum states may drift, making earlier stored states less relevant or even unusable. This limits the effectiveness of quantum repeaters or state buffering techniques during UAV–vehicle QC sessions.

**Figure 5:**
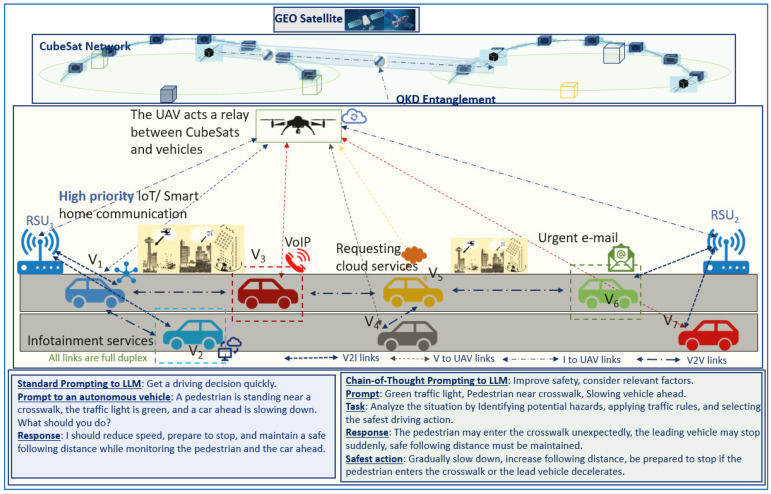
Future vision of a quantum-enhanced, LLM-enabled SAGIN. Autonomous vehicles maintain connectivity through coordinated terrestrial, UAV, and LEO CubeSat networks. Vehicle-generated multimodal sensor data is processed by LLMs at edge servers for perception, decision-making, and network optimization. Limited vehicular resources motivate NISQ devices or embedded qubits for quantum-assisted LLM inference, enabling hybrid classical–quantum communications. UAVs provide flexible airborne links, CubeSats extend global coverage, and terrestrial systems ensure high-capacity access. Quantum-enhanced LLMs enable distributed inference, multimodal fusion, adaptive resource management, and secure, resilient SAGIN operations in mission-critical scenarios.

Figure 5 illustrates a future vision of a quantum-enhanced, LLM-enabled SAGIN supporting autonomous vehicular communications. In this architecture, autonomous vehicles maintain continuous connectivity through LEO CubeSats and UAVs. Sensor data collected by vehicles is processed using LLMs, which provide high-level reasoning and decision-making capabilities. However, LLMs are resource intensive, and their deployment on vehicular edge servers depends on platform capabilities and manufacturer-specific hardware. Additionally, effective LLM deployment requires scenario-specific dataset preparation, vision-model construction, and model fine-tuning. As sensor data volumes, model sizes, and prompt computations continue to grow, the associated computational and storage demands often exceed the limited resources available on vehicular edge platforms. To address these constraints, several emerging works propose executing LLM workloads on NISQ devices or qubits embedded within vehicular edge servers. The resulting quantum-processed information can then be transmitted to other vehicles or infrastructure nodes over classical communication channels, creating hybrid classical–quantum interactions.

Furthermore, this approach enables distributed processing of complex LLM tasks while accommodating the resource limitations of edge platforms. Figure 5 also illustrates an autonomous driving scenario comparing standard prompting and chain-of-thought prompting for LLM-based decision-making. In standard prompting, the LLM generates a direct driving action in response to a situation, for example, a pedestrian near a crosswalk, a green traffic light, and a slowing leading vehicle. This produces a concise response, such as reducing speed, maintaining a safe following distance, and preparing to stop, prioritizing speed and simplicity for low-risk or time-critical situations. In contrast, chain-of-thought prompting guides the LLM to explicitly analyze potential hazards, apply traffic rules, and determine the safest driving action. The resulting structured reasoning considers pedestrian unpredictability, sudden braking by the leading vehicle, and right-of-way regulations. While standard prompting enables rapid responses, chain-of-thought prompting supports safer and more interpretable decision-making when combined with rule-based safety mechanisms and sensor fusion in complex, real-world autonomous driving scenarios.

The high mobility of UAVs poses challenges for integrating quantum hardware into SAGIN. Quantum links, including entanglement distribution and quantum key distribution, are sensitive to alignment, pointing accuracy, and channel stability. Rapid UAV movement introduces variations in line-of-sight paths, Doppler shifts, and pointing errors, degrading quantum state fidelity and link reliability. To mitigate these issues, UAV-mounted quantum hardware requires lightweight, robust optical terminals with fast beam-tracking, adaptive error correction, and stabilization mechanisms. High-mobility UAVs may also adopt hybrid strategies, performing short-range quantum operations onboard while offloading long-distance, sensitive tasks to ground stations or high-altitude platforms with lower relative mobility. Addressing these challenges is critical for effectively implementing quantum primitives in UAV-assisted SAGIN links while ensuring security and reliability.

### 6.5. Key Findings

Table 9 provides a detailed summary of recent works on integrating LLMs in SAGINs. It highlights the proposed contributions, methodologies, and identified research gaps, emphasizing the convergence of LLMs, quantum communication, and AI-driven orchestration for 6G networks. The methodologies cover a wide range of approaches including RAG-based multimodal knowledge retrieval, low-power deployments, quantum-secured communication, entanglement-assisted links, energy-aware device orchestration, ultra-reliable low-latency communication, AI-assisted resource monitoring, and digital twin-based decision-making. The table also identifies critical challenges such as efficient deployment in massive heterogeneous networks, latency and reliability trade-offs, energy constraints, coexistence with legacy systems, dynamic collision handling, and context-aware AI deployment at scale. Overall, this summary underscores the potential of combining LLMs, quantum technologies, and intelligent orchestration for resilient, energy-efficient, and ultra-low-latency SAGIN operations.

Table 10 summarizes the key challenges in implementing quantum communication between UAVs and vehicles, along with their corresponding impacts on system performance. The table highlights fundamental limitations such as the restricted number of usable qubits, which constrains onboard algorithmic complexity, and the sensitivity of quantum measurements to environmental disturbances, mobility-induced noise, and vibrations, leading to rapid decoherence. It also emphasizes the inherent inability to amplify quantum signals due to the no-cloning theorem, limiting communication range and increasing susceptibility to atmospheric attenuation. Scalability issues arise from hardware constraints, limited inter-qubit connectivity, and cooling or shielding requirements, which hinder the deployment of large quantum registers on mobile platforms. Additionally, the low error tolerance of quantum states and the practical challenges in quantum error correction reduce link reliability. Finally, preserving quantum states during UAV motion is challenging, as shifting reference frames and trajectory dynamics degrade stored quantum information, further impacting overall fidelity. This summary underscores the significant technical obstacles that must be addressed to achieve robust UAV–vehicle quantum communication.

### 6.6. Open Issues in SAGIN Communications

Table 11 summarizes recent works in 6G and SAGIN, highlighting the proposed concepts, methodologies, and identified research gaps. The table identifies limitations in existing studies, including challenges in multi-band interoperability, scalability in dense UAV and CubeSat deployments, synchronization and CSI sharing overhead, interference management in multi-layer networks, and the limited integration of intelligent edge processing and adaptive resource allocation. Our analysis addresses these challenges in several ways. First, we explore adaptive and cooperative strategies across UAVs, CubeSats, and terrestrial nodes to enhance network reliability, coverage, and throughput. Second, we investigate dynamic spectrum management, integrated access and backhaul, and multi-layer coordination for optimizing end-to-end performance. Finally, we emphasize the role of intelligent edge processing, including quantum-enhanced decision-making, to handle heterogeneous, multimodal, and non-i.i.d. data across SAGIN nodes. Table 11 highlights these contributions, emphasizes the research gaps we address, and underscores the novelty and relevance of LLMs in SAGIN.

Table 12 summarizes key references in terahertz (THz) and optical wireless communication systems, highlighting proposed concepts, methodologies, and identified research gaps. The table identifies limitations in existing works, including sensitivity to phase noise, hardware and power constraints, challenges in adaptive beamforming, and computational complexity for ultra-high data rate processing. It also points out issues with accurate channel modeling, trajectory tracking, and integration of hybrid optical–radio frequency and multi-access schemes. Our survey identifies these challenges by exploring intelligent signal processing, adaptive resource allocation, advanced modulation, and multimodal integration strategies. Additionally, the table emphasizes the need for scalable, energy-efficient, and low-latency designs to enable reliable terabit-level THz links and resilient optical wireless communication systems for next-generation 6G networks. Table 12 highlights critical contributions, clarifies research gaps, and underscores the practical relevance of advanced THz and optical communication solutions.

#### 6.6.1. Optical Wireless Communication (OWC) Networks for High-Fidelity Semantic Transmission

Beyond the THz spectrum, optical wireless communication (OWC) networks are being explored to provide broadband connectivity in the optical frequency range, including the infrared (187–400 THz, 750–1600 nm), visible (400–770 THz, 390–750 nm), and ultraviolet (1000–1500 THz, 200–280 nm) bands [224]. OWC offers extremely high bandwidth, robustness to electromagnetic interference, high spatial confinement, enhanced security, and operation in unlicensed spectrum [64]. Free-space optical communication, particularly infrared OWC systems, is commonly deployed for long-range, high-speed point-to-point links such as ultra-broadband wireless backhaul, and, to a lesser extent, for indoor communications [225].

Visible light communication is an OWC technology operating in the visible spectrum and is a promising solution for local broadband connectivity [217]. In visible light communication systems, all baseband processing at the transmitter and receiver occurs in the electrical domain. Data is encoded and transmitted over line-of-sight or non-line-of-sight (NLoS) optical channels using light-emitting diodes with wide fields of view or laser diodes with narrow fields of view [64]. At the receiver, photodetectors convert the data-carrying light back into electrical signals for baseband processing. Commercial optical transceivers typically achieve data rates up to 100 Mbps, while laboratory demonstrations have reached hundreds of Gbps [226]. Because light-emitting diodes and photodetectors are well-established, low-cost, and commercially available, visible light communication is being explored to complement existing communication technologies [223].

However, integrating LLMs into OWC networks presents unique challenges. LLMs, which require substantial computational resources for real-time data processing and semantic interpretation, introduce a layer of complexity to the communication architecture. The primary challenge is the need for the efficient, low-latency processing of large-scale language models at the edge of the network, where bandwidth and computational resources may be constrained. Additionally, the seamless integration of LLMs with OWC systems demands reliable, high-speed data transmission with minimal interference, especially when dealing with real-time content generation and contextual understanding. The deployment of such models on edge devices or even within the network could introduce additional overhead in terms of both computation and data transmission, potentially reducing the system’s overall performance.

Quantum communication, while offering promising advantages in terms of security and unbreakable encryption, also poses significant challenges for integration into OWC networks. The inherent fragility of quantum states makes it difficult to maintain stable communication over long distances, especially in free-space optical channels. Quantum key distribution, which relies on the transmission of quantum states such as photons, requires precise alignment and low-loss transmission paths, something that is hard to achieve in dynamic, NLoS environments. The integration of quantum communication systems with OWC networks requires overcoming issues related to signal attenuation, atmospheric interference, and the need for specialized hardware capable of handling quantum bits (qubits) alongside classical data. Moreover, the current technology for quantum repeaters and secure transmission over long distances is still in development, making its practical application in large-scale OWC networks an open research avenue.

#### 6.6.2. Challenges in Integrating LLMs and Quantum Communication in Long Range Cell Free Massive MIMO Networks

Long-range massive MIMO enhances the range and coverage of access points (APs) and improves channel capacity for long-distance communications. Coherent joint transmission between adjacent APs boosts performance at cell edges, creating a long-range cell-free network. However, line-of-sight (LoS) channels are vulnerable to shadowing and blocking, leading to coverage gaps. This method requires higher backhaul capacity and faces synchronization issues due to propagation delays [227]. Integrating LLMs into this architecture introduces additional challenges. LLMs require substantial computational resources and low-latency access to data, which can strain the backhaul and edge-processing capabilities of long-range massive MIMO systems. The distributed nature of cell-free architectures complicates the deployment of LLM-based semantic processing, as coordinating model inference across multiple APs demands strict synchronization and reliable high throughput links.

Furthermore, the dynamic nature of wireless environments can hinder the consistent delivery of the large parameter updates or contextual data needed by LLMs, potentially degrading the quality of semantic communication. Quantum communication also presents significant challenges for integration into this architecture. Quantum states are extremely sensitive to noise, atmospheric conditions, and hardware imperfections, making their transmission across long-range massive MIMO or coordinated AP deployments difficult. Quantum communication and other quantum protocols require precise timing, alignment, and minimal loss channels, which are hard to achieve in a distributed network with varying propagation delays. Additionally, the coexistence of classical and quantum signals in the same infrastructure demands specialized transceivers and strict isolation mechanisms, further complicating system design and increasing implementation complexity.

#### 6.6.3. LLM Computational Strain and Quantum Channel Fragility in High-Frequency LEO, HAPS, and IRS Enabled Networks

Utilizing millimeter-wave (mmWave) and THz frequencies from LEO satellites enhance rural coverage, particularly when combined with HAPS. However, deploying many LEO satellites is costly and increases interference. Intelligent reflecting surfaces (IRSs) can mitigate coverage gaps by directing signals to weak areas and are powered by renewable sources, making them a low-cost alternative to additional access points [228]. However, their effectiveness decreases with increasing distance between the source and destination. The integration of affordable computing and vast IoT data supports edge and fog computing, emphasizing the need to transition from centralized cloud computing to more localized solutions [229]. Integrating LLMs into this architecture requires substantial computational resources and low latency access to distributed data, which strains the limited processing capabilities available on LEO satellites, HAPS, and IRS-assisted links.

The highly dynamic nature of mmWave and THz channels further complicates the deployment of LLM-based semantic processing, as maintaining stable and high throughput connections for model inference or updates becomes difficult. In addition, coordinating LLM operations across heterogeneous nodes, including edge devices and satellite platforms, demands precise synchronization and efficient resource allocation, which are challenging in these network environments. Quantum communication also poses challenges when integrated into architectures that rely on mmWave and THz frequencies with LEO satellites and HAPS. Quantum signals are extremely sensitive to noise, atmospheric absorption, pointing errors, and Doppler effects, which are exacerbated in high-mobility and long-distance satellite links. Implementing quantum communication and other quantum protocols requires low loss optical or specialized quantum channels, which cannot be easily guaranteed in systems that rely on high-frequency radio links or IRS-assisted paths. Furthermore, the coexistence of classical and quantum communication in shared infrastructures necessitates additional hardware complexity, precise alignment, and strict isolation, making large-scale quantum integration technologically challenging.

#### 6.6.4. Integration Challenges for LLM Semantics and Quantum Links in High-Frequency SAGIN Systems

Free-space optics in various frequency bands support high-speed links, while fixed wireless access offers effective solutions for last-mile connectivity [76]. These networks, which must provide ubiquitous coverage, include terrestrial mobile networks, geostationary and non-geostationary satellites, HAPS, and high-altitude intelligent balloon systems. Additionally, free-space optics operating in *Q*, *V*, *E*, *D*, and *W* bands complement fiber-based backhaul and support high-speed links, while fixed wireless access using millimeter-wave and *V*-band frequencies offers cost-effective solutions for last-mile connectivity [58]. Furthermore, the [95 GHz, 3 THz] bands have recently been opened by the U.S. Federal Communications Commission (FCC) for experimental use, enabling the research and development of higher-frequency communication technologies. The transition to higher-frequency bands does not aim to merge existing technologies into a single wireless interface; rather, it focuses on supporting seamless mobility across diverse frequencies [209].

In massive MIMO, access points with large antenna arrays serve multiple users simultaneously. Dense deployments of low-cost access points and fog nodes, with users located near multiple access points, reduce path loss and improve diversity. Unlike Coordinated Multi-Point (CoMP) and traditional MIMO, cell-free networks adopt a user-centric approach, providing nearly uniform performance across the coverage area rather than maximizing peak data rates [210]. Integrating LLMs and quantum communication into this architecture poses significant challenges. LLM inference demands high computational power and low-latency access to distributed data, straining edge resources and complicating synchronization across multiple access points. Quantum communication adds further complexity, as quantum states are highly sensitive to noise, atmospheric attenuation, and alignment errors, making stable THz-frequency transmission difficult. Coexistence of classical and quantum links increases hardware complexity and requires precise coordination, further complicating integration within cell-free massive MIMO systems.

#### 6.6.5. Challenges of CSI and Synchronization in Dense Cell-Free Massive MIMO Networks

Cell-free massive MIMO relies on a dense deployment of access points connected by suitable fronthaul links in crowded areas with high traffic [24]. These networks operate across various frequency bands, achieving high data rates over short distances, especially when multiple neighboring access points provide connectivity. Integrated access and backhaul networks are crucial in dense environments, particularly at mmWave frequencies, where fiber connections to all access points may be costly [212]. In integrated access and backhaul setups, a few fiber-connected access points offer wireless backhaul to others while serving mobile devices, using the same spectrum for both roles. This approach varies from traditional relays, as the data load differs across hops and interference depends on aggregated traffic [213]. Access procedures in cell-free networks differ from traditional systems, requiring cooperative efforts from neighboring access points and new synchronization mechanisms [230]. While offering scalable and energy-efficient connectivity for 6G applications, cell-free networks face challenges with CSI exchange. Access points use locally acquired CSI for precoding, while drones handle signal processing. Synchronization allows for signal combination without explicit CSI sharing, but sending CSI to the UAV for centralized processing increases complexity and latency [211].

#### 6.6.6. Synchronization and Connectivity Challenges in SAGIN with IAB

As 6G networks grow denser and more spectrum diverse, integrated access and backhaul will be vital for efficient deployment. An integrated space and terrestrial network consists of spaceborne, airborne, and ground-based layers. The spaceborne layer includes LEO satellites, which provide broadband due to their proximity, improving signal-to-noise ratio and reducing latency [24]. However, extensive ground coverage requires many LEO satellites, and their motion complicates synchronization. Satellite communication links primarily use LoS, introducing delays over long distances. Traditional high-gain antennas are less flexible for mobile use, and interference limits performance.

The airborne network serves as a vital layer, offering high-speed aerial connectivity through technologies like mmWave links, adaptive antenna arrays, and massive MIMO. Solar panels on CubeSats enhance energy efficiency by providing a consistent power source [214]. The airborne communications network must maintain reliable connectivity while CubeSats and vehicles are in motion, requiring precise trajectory tracking and adaptive beamforming. In cases where channel state information for coherent spatial multiplexing is unavailable, space–time coding combined with adaptive beamforming is essential [215]. CubeSats’ processing and transmit power are also limited by harvested solar energy. Dynamic topology, intermittent satellite transmissions, and multi-layer interference add complexity to SAGIN management [216].

#### 6.6.7. Adaptive Beamforming and MIMO Challenges in Sub-THz Networks

Single-carrier links are conventionally preferred for sub-THz links due to their simplicity and efficiency, although they are affected by phase noise. Novel envelope detection receivers are being proposed for sub-THz communications to facilitate frequency down-conversion [231]. MIMO systems with energy detection ensure high spectral efficiency while maintaining low power and complexity in conditions challenging for traditional coherent detection. Despite exhibiting quasi-optical propagation characteristics, THz communications retain several microwave-like features [217].

Intelligent reflecting surfaces enhance NLoS propagation, while ultra-massive MIMO techniques support efficient beamforming [220]. As digital beamforming evolves, hybrid or analog methods using adaptive array-of-subarrays are also increasingly used. Techniques such as index modulation, high-rate impulse radio, and the joint optimization of analog and digital signal processing are being explored for energy and complexity-constrained systems [219]. As digital beamforming capabilities evolve, hybrid or analog beamforming methods employing adaptive array-of-subarrays architectures could be implemented, allowing each subarray to perform independent analog beamforming [221].

#### 6.6.8. THz Networks: LLM and Quantum Communication Challenges

At higher THz frequencies, molecular absorption becomes increasingly significant, resulting in a spectrum divided into multiple transmission windows, each ranging from tens to hundreds of gigahertz in width for high-speed communication [232]. Single-carrier modulation schemes can support data rates exceeding tens of gigabits per second [233]. Beyond conventional schemes, dynamic bandwidth algorithms have been proposed to adapt to the distance-dependent, absorption-limited channel bandwidth for both short and long-range links [222]. However, efficient resource allocation strategies that jointly manage frequency, bandwidth, and antenna resources are yet to be realized to fully exploit the THz band. Another major challenge arises from the digitization of large-bandwidth signals [43]. Future communication systems target extreme data rates, with peak values reaching 1 Tbps and user experience rates of 1 Gbps across all data transfers [1]. Regardless of user location, these systems aim for high spectral efficiency, with peak values of 60 b/s/Hz and an average per-user spectral efficiency of approximately 3 b/s/Hz. Bandwidths of up to 10 gigahertz in mmWave bands and 100 gigahertz in THz and visible light bands are envisioned [12]. Energy efficiency targets 1 Tb per Joule, while ultra-low latency of 0.1 ms with jitter below 1 µs is essential. Extremely high reliability is also required to support dense deployments and emerging applications that demand consistent connectivity under highly dynamic network conditions [232].

Although THz channels support bandwidths exceeding 100 gigahertz, the sampling rates of current digital-to-analog and analog-to-digital converters are limited to about 100 gigasamples per second. Consequently, highly parallelized systems and efficient signal processing architectures are required [234]. Channel coding, a computationally intensive component of baseband processing, demands efficient parallel schemes to support terabit-per-second operations in wireless backhaul, where long transmission distances require high-gain directional antennas and ultra-narrow beamwidths [13]. In high-mobility scenarios, ultra-fast data transfers are needed to mitigate intermittent connectivity between vehicles and infrastructure, requiring reliable, low-latency links [3]. As data traffic grows, broadcast and multi-cast networks become increasingly important for efficiently delivering the same information to multiple vehicles. Current 5G new radio systems use orthogonal frequency-division multiplexing (OFDM) and discrete Fourier transform-spread OFDM for improved uplink coverage and power efficiency, while single-carrier waveforms such as discrete Fourier transform-spread OFDM are more robust to phase noise [217]. For THz communications above 100 gigahertz, water vapor absorption creates distinct transmission windows with minimal atmospheric loss. The small effective areas of terahertz antennas necessitate high-gain directional designs or dense arrays of low-gain elements [218].

#### 6.6.9. Visible Light Communication and Hybrid Optical–Radio Frequency Networks for Efficient Data Transmission

Optical links are crucial for data transmission and energy harvesting, facilitating energy-autonomous devices. Visible light communication is particularly effective in environments where traditional radio frequency communications fail, such as in-cabin airplane connectivity and underwater communications [235]. However, visible light communication relies on intensity modulation with direct detection, which imposes limitations on signal transmission. To overcome these limitations, deploying a hybrid optical–radio wireless network create a flexible communication system that adapts to changing environments [236]. Accurate visible light communication channel modeling is essential, particularly regarding vehicle mobility and orientation, while integrating visible light communication with radio frequency systems with transmission schemes such as spatial modulation and advanced optical MIMO techniques to enhance performance.

While high-order quadrature amplitude modulation enhances spectral efficiency, its advantages may decrease due to hardware nonlinearities [237]. Power-domain NOMA enables users to decode messages based on SNR levels, while code-domain NOMA enhances SNR at the cost of added interference. Rate splitting further divides messages into private and common parts for efficient data recovery. Iterative algorithms focus on training communication systems with unknown channels and ensuring synchronization, covering channel estimation, equalization, and signal detection [238]. Integrating LLMs into hybrid optical–radio frequency architectures presents substantial challenges, as coordinating model inference across multiple devices while maintaining real-time performance strains edge and fog computing resources. Quantum states are highly sensitive to noise, alignment, and interference in optical and radio frequency channels. Ensuring stable quantum communication and entanglement-based communication requires precise control and specialized hardware, increasing system complexity and design constraints in these hybrid networks.

Hence, LLMs are expected to be deployed to enhance network intelligence and user experience. Cloud LLMs support global optimization and long-term planning, while edge LLMs provide domain specific reasoning with low latency for RAN control. On-device LLMs consist of compact distilled models that ensure privacy and enable semantic compression. Intent-based networking maps high-level goals to resource allocation decisions, and semantic communications focus on transmitting meaning rather than raw data, with $I_{\mathrm{semantic}}\ll I_{\text{bit-level}}$. LLMs also facilitate automated failure diagnosis using multimodal inputs and support human-centric interfaces for natural language interaction. Despite their promise, LLMs introduce several challenges that must be addressed for reliable deployment in communication networks. Latency and determinism are critical, as inference time $\tau_{\mathrm{LLM}}$ must satisfy real time control constraints $\tau_{\mathrm{LLM}}\leq \tau_{\mathrm{ctrl}}$. Trust and safety require mitigating hallucinations and adversarial threats through grounding and verification mechanisms. Privacy and governance are essential for the secure handling of sensor and user data included in prompts. Additionally, energy efficiency is a key concern, demanding strategies to reduce the computation and carbon footprint associated with large-scale LLM inference while maintaining performance and responsiveness.

### 6.7. Incorporating Protocol Learning in 6G SAGINs

Protocol learning is vital for next-generation networks, particularly in 6G SAGIN. It supports adaptive and self-optimizing communication protocols through DRL, LLM-based decision-making, and distributed intelligence. This enables nodes, including UAVs, CubeSats, and terrestrial platforms, to autonomously adjust routing, scheduling, and resource allocation based on dynamic network conditions. Moreover, protocol learning enhances the semantic-driven edge intelligence by optimizing communication protocols in real time. The authors in [239] also highlight the potential of integrating protocol learning with quantum-enhanced LLMs to improve adaptability, reliability, and overall network performance in complex and mobile 6G environments [239]. In the proposed quantum-enhanced edge intelligence framework, it is crucial to distinguish between tasks that require quantum computing and those manageable by advanced classical architectures. Quantum computation is needed for large-scale optimization in network resource allocation, secure coordination using entanglement, and specific quantum ML tasks that offer exponential speedup. Conversely, many inference and semantic processing tasks such as multimodal feature extraction, RAG, and channel-adaptive semantic encoding can be handled effectively by advanced classical systems like GPUs and specialized AI accelerators. Additionally, edge-level decision-making, digital twin synchronization, and task-oriented decoding can benefit from high-performance classical computing with model compression techniques. By mapping tasks to appropriate resources, designers can optimize resource use, simplify hardware complexity, and deploy quantum-enhanced edge intelligence effectively across integrated networks spanning space, aerial, and ground nodes [239].

## 7. Conclusions and Future Work

This paper investigated the integration of UAVs, CubeSats, and terrestrial systems in 6G networks to support SAGIN for IMT 2030. It analyzed how UAVs enabled flexible airborne connectivity, CubeSats extended global low-latency coverage, and terrestrial infrastructures utilize uninterrupted high-bandwidth communication. The study emphasized interoperability, coordinated resource allocation, and seamless interaction among SAGIN nodes for ultra reliable communication. It further examined LLMs for intelligent network management and quantum communication for security and latency reduction. Numerous existing works have hypothesized that in the next decade, quantum-enhanced LLMs will be able to provide a resilient way to improve routing, bandwidth allocation, resilience, privacy, and adaptive edge intelligence for overall effectiveness.

The survey revealed that the integration of LLMs, traditionally dependent on cloud infrastructures due to their substantial computational requirements, introduces a new paradigm when synergized with quantum-enhanced communication links. Quantum communications significantly improve the reliability, security, and latency of data exchange between edge devices and central processing units, thereby enabling smaller-scale models known as SLMs with fewer than a billion parameters to operate efficiently on mobile and edge platforms. These edge-deployed SLMs, characterized by reduced energy consumption and improved responsiveness, support a wide range of applications such as real-time translation, transcription, generative image editing, and personalized content management. Consequently, reliance on centralized cloud systems is minimized, fostering more natural and localized user interactions. In vehicular networks, for example, mobile and edge LLM agents collaboratively generate real-time accident reports. Mobile agents perceive the surrounding environment and share intermediate observations with edge agents, which aggregate the contextual information and provide optimized strategies back to mobile agents for localized decision-making. This cooperative paradigm promotes context-aware and adaptive communication across heterogeneous network layers.

This survey presented six key contributions advancing quantum-enhanced edge intelligence in SAGINs with LLM integration. First, we examined how quantum-assisted LLMs enable real-time decision-making and efficient network management in 6G SAGINs, acting as distributed cognitive agents for context-aware optimization. Second, we evaluated distributed quantum inference across UAVs, CubeSats, and terrestrial nodes, highlighting gains in latency, reliability, and scalability. Third, we assessed quantum-assisted multimodal data fusion for bandwidth-efficient and interoperable communications. Fourth, we integrated quantum-secure links with LLM-based semantic control to enhance privacy and data security. Fifth, we investigated LLM-driven edge intelligence for UAV and CubeSat nodes, emphasizing context-aware learning and self-organizing behaviors. Finally, we proposed performance metrics and benchmarking frameworks to quantify the impact of quantum-enhanced LLM integration on latency, reliability, and throughput. Together, these contributions address critical research questions on LLM deployment, quantum-assisted optimization, and secure adaptive communications, providing a foundation for future sustainable and intelligent SAGIN architectures.

Future research should focus on enhancing the performance of LLMs in dynamic wireless environments while addressing privacy and data protection concerns during collaborative inference. Although the application of LLMs in telecommunications is still in its infancy, their potential for network specification management, anomaly detection, and performance optimization is considerable. The integration of textual, visual, and domain-specific data further enhances capabilities such as spectrum management, semantic reasoning, and localization in 6G networks. Energy efficiency, explainability, and ethical deployment will be essential to ensure the responsible and sustainable utilization of LLMs in next-generation communication systems. Furthermore, the development of domain-specific datasets and quantum-assisted training frameworks will be instrumental in realizing intelligent, autonomous, and resilient communication within SAGINs. As a future work, the authors aim to investigate the role of LLMs in enhancing environmental cognition, scene perception, obstacle avoidance and computer vision in connected and autonomous vehicles. Next, the authors aim to process these LLMs on NISQ qubits and study the trade-off between fidelity, entanglement and latency in the safe driving of SAGIN-assisted connected and autonomous vehicles.

## Figures and Tables

**Table 1:** A survey of LLM-based communications in SAGIN.

Identified Challenges in Existing Papers	Proposed Solutions in Existing Papers	Adopted Solution Methodology and Approach
Integration of UAVs, CubeSats, and Geostationary satellites in 6G	Multi-agent collaboration across heterogeneous nodes	Designed as a collaborative multi-agent system where UAVs, CubeSats, and terrestrial nodes coordinate to support seamless connectivity and adaptive task allocation.
SAGINs as an integral part of IMT-2030 framework	Collective intelligence alignment across underlying layers	Implement unified control and resource allocation mechanisms to align decision-making among space, aerial, and terrestrial components for robust performance under dynamic conditions.
6G goals: ultra-low latency, high reliability, massive connectivity	Adaptive optimization of communication objectives	Employ real-time optimization techniques and coordinated scheduling to balance latency, reliability, and throughput across diverse agent networks.
UAVs and CubeSats to enhance coverage and capacity	Coordinated autonomy for extended coverage	Integrate distributed learning and coordination protocols to enable UAVs and CubeSats to dynamically extend coverage while maintaining consistent performance metrics.
LLMs for intelligent network management and data optimization	Cognitive and cooperative role for multi-agent decision-making	Utilize LLMs as cognitive agents to generate semantic representations, resource allocation, and optimize multimodal data processing across SAGIN nodes.
Quantum communication for secure and low-latency data transmission	Trust and alignment layer for secure coordination	Incorporate quantum-enhanced communication channels to support privacy-preserving, low-latency data exchange across distributed agents.
LLMs and quantum edge intelligence as an emerging research problem	Evolving collaborative paradigm for adaptive control, cooperation and perception	Develop frameworks for LLM-driven quantum edge intelligence to enable context-aware, adaptive, and coordinated decision-making.
Quantum-assisted parallelism and entanglement-based optimization	Distributed reasoning and workload partitioning	Apply quantum-assisted parallelism to accelerate joint optimization, multi-agent inference, and real-time decision-making under high data volume.
Distributed quantum inference and multimodal fusion	Unified decision-making process across modalities	Implement distributed multimodal fusion and quantum-enhanced inference to ensure coherent, self-optimizing communication in SAGIN.
LLMs as cognitive control centers for mission-critical communications	Adaptive coordination engine for critical tasks	Leverage LLMs to orchestrate multi-agent operations, maintain situational awareness, and support dynamic, high-priority communication flows.
Performance metrics: energy efficiency, reliability, adaptive learning	Collaborative performance metrics	Measure energy efficiency, reliability, and adaptive learning as indicators of effective multi-agent coordination and cognitive partner contributions.
Quantum-enhanced LLMs addressing bandwidth, routing, and interoperability	Joint optimization strategy across nodes	Use quantum-enhanced reasoning to simultaneously optimize bandwidth allocation, dynamic routing, and interoperability among heterogeneous agents.
Privacy, security, and future potential of quantum-empowered LLMs	Secure multi-agent alignment	Design mechanisms to ensure privacy, trust, and long-term alignment of multi-agent objectives under scalable, distributed conditions.
Overly detailed environment listing that mislead LLMs	Context-aware abstraction for multi-agent deployment	Generalize environmental descriptions while retaining key distinctions such as remote and urban regions to guide adaptive deployment strategies.

**Table 2:** Estimated energy consumption of LLMs on UAV platforms.

LLM Size (Parameters)	Platform Type	Inference Mode	Power Consumption	Remarks
125–350 million	Small quadcopter UAV	Onboard real-time	10–30 W (40–44.8 dBm)	Feasible with lightweight models and short flight durations; suitable for basic tasks without heavy computation.
1–2 billion	Medium UAV/Edge AI Chip	Onboard batched	50–120 W (47–50.8 dBm)	Requires quantization or model compression to reduce power demands while maintaining real-time processing capabilities.
6–13 billion	Large UAV/FPGA or GPU-equipped	Offloaded/ Collaborative	150–300 W (51.8–54.8 dBm)	Offloading to edge or cloud is preferred for real-time tasks due to high onboard power consumption and processing requirements.
≥30 billion	High-end UAV or Ground-edge hybrid	Cloud-assisted only	≥500 W (57 dBm)	Not feasible for standalone UAV operation; suitable for offloaded inference with high-bandwidth connectivity.

**Table 3:** Edge AI, user context, and distributed intelligence in 6G networks.

References	Proposed Work	Methodology	Identified Gaps
[64]	Synthetic data generation and model improvement via generative adversarial networks (GANs) and incremental learning for heterogeneous data fusion at the edge.	Feature extraction, representation learning, split learning, generalized adversarial networks to ensure sensor data consistency.	Limited data availability; heterogeneous sensor data integration; ensuring consistency across distributed models.
[65]	Integration of diverse data such as local weather and traffic for network management and real-time adaptability.	Mobile cloud and mobile edge computing platforms; uniform interfaces for data and AI model interoperability.	Latency due to large, distributed deployments; challenges in maintaining interoperability.
[59]	Lightweight AI for autonomous edge devices; deployment using virtual machines; optimization of real-time feedback cycles.	Distribution of pre-trained and online-learned models; efficient computation and communication at resource-constrained edge nodes.	Energy consumption, storage limitations, and device mobility affecting algorithm performance.
[67]	Exchange of raw data, model parameters, or inferred outputs among edge devices.	Scheduling of resources, communication-aware distributed algorithm design.	Communication uncertainties; constrained bandwidth; trade-off between privacy, energy, and latency.
[13]	Privacy-preserving FL with differential privacy and homomorphic encryption.	Keep raw data local; share model parameters securely.	Ensuring lightweight security mechanisms while maintaining inference accuracy.
[68]	Understanding user context and behavioral patterns to adapt edge resources.	Incentive mechanisms, lightweight consensus protocols, virtualization and containerization for resource management.	Mobility management and multi-tenant privacy concerns; dynamic resource allocation challenges.
[69]	QoS enhancement for XR and latency-sensitive applications.	Data intelligence, multi-level optimization, novel quality metrics beyond throughput and latency.	Limited QoS levels in existing networks; requirement for new end-to-end delay metrics.
[70,71]	Payload customization and semantic-aware networking.	Qualitative payload marking, entropy-based redundancy detection, random linear network coding.	Inefficient retransmission for large packets; need for adaptive prioritization of critical data.
[72]	Cross-layer innovations leveraging IP extensibility and embedded contracts.	Analytics at network core and edges; hardware acceleration.	Integration complexity for control, routing, and management protocols in 6G.
[73]	Machine learning-based analytics for self-organizing and self-healing networks.	Integrated intelligence across service layer, RAN, and core network; high-precision sensor data analysis.	Real-time adaptation challenges; need for automation in distributed network management.
[74]	Automated vRAN management with AI-enabled analytics.	Standardized vRAN interfaces; MDAF for performance and fault aggregation; SLA enforcement and QoS prediction.	Scalability and resource optimization in complex vRAN deployments.
[21,24]	Smart sensor deployment and in-network preprocessing with LLMs.	Context-aware data aggregation; resolution adjustment; query-based extraction.	Managing massive sensor data volumes; balancing precision, latency, and processing overhead across cloud, transport, and RAN segments.

**Table 4:** Role of LLMs and deep learning in 6G wireless networks.

References	Proposed Work	Methodology	Identified Gaps
[81]	LLMs for modeling complex 6G network interactions, replacing heuristic optimization, enabling real-time automated operations and intelligent network control.	End-device computations with predictions fed back to network; distributed network intelligence; dynamic deployment at management, core, and mobile device levels.	Dependence on timely, latency-sensitive data; efficient data transfer mechanisms required; large-scale deployment challenges.
[83]	Integration of LLMs for resource management and automated network control.	Computation on mobile devices; prediction aggregation; network-level decision-making.	Avoiding transmission of unused data; dynamic placement optimization.
[84]	DL architectures (CNNs, autoencoders, GANs) for 6G applications.	Supervised learning on labeled datasets; classification and regression tasks.	Limited availability of large training datasets; generalization to diverse real-world network conditions.
[82]	Addressing heterogeneity of mobile operators and data confidentiality for DL deployment.	Cross-operator learning; platform-specific and application-specific model combination.	Standardization alone insufficient for interoperability; limited shared datasets.
[86]	Probabilistic ML and Bayesian inference for 6G, uncertainty quantification, and robust decision-making in noisy environments.	Non-parametric Bayesian methods, e.g., Gaussian processes; Variational Bayes, Expectation Propagation, MCMC.	Computational complexity; scalability in high-dimensional, spatio-temporal problems.
[88]	reproducing kernel Hilbert space methods to enhance DL inputs with well-regularized features and fewer hyperparameters.	Feature engineering; integration with DL models.	Limited adoption in practical 6G deployments; computational trade-offs.
[91]	Adaptive online learning for mobile positioning and multi-user environments.	Synchronization signal-based FEC and classification; RSS/CSI-based fingerprinting; adaptive learning for NLoS multipath conditions.	Performance drops in dynamic, uncontrolled environments; missing measurements; model adaptability required.
[78]	Deep learning-based channel estimation and real-time non-convex optimization (e.g., throughput maximization, beamforming).	Offline and online DL models; iterative optimization; deep learning for real-time control.	Offline-trained models may underperform in dynamic conditions; high computational requirements for real-time execution.

**Table 5:** Deep learning, LLMs, and semantic-aware approaches in 6G networks and vehicular systems.

References	Proposed Work	Methodology	Identified Gaps
[93]	CNNs for signal classification; deep neural networks for channel estimation and signal detection; multi-input/multi-output downlink beamforming.	End-to-end physical layer optimization using deep learning; autoencoders for transmitter-receiver design; Monte Carlo-style simulations; channel models with noise and multipath effects.	High computational cost; offline training may not generalize across diverse scenarios; adaptation across multiple environments needed.
[86,94]	Intelligent deep reinforcement learning for optimization in vehicular networks; FL for user location prediction and VR QoE improvement.	Training under varying distance, speed, environment, and weather conditions; historical data-driven FL.	Ensuring performance consistency across scenarios; high mobility and dynamic conditions may reduce accuracy.
[27,72]	Integrating multi-cell data to enhance proactive MAC functions; LLM-enabled resource allocation, traffic prediction, and mobility management in MTC networks.	Optimization and cross-cell data integration; LLM-assisted decision-making for resource allocation.	Multiplexing limitations and self-interference; resource underutilization; dynamic deployment and real-time adaptability challenges.
[96]	LLM-based security solutions for SAGIN communications; context-aware traffic classification and resource allocation.	Predictive LLMs for dynamic threat management; sensor fusion; context-aware systems for automated control.	Complexity in distinguishing legitimate vs malicious traffic; real-time adaptation under heterogeneous devices.
[30,98,99]	UAV and vehicular networks with LLM-enabled control and opportunistic data transfer.	UAV trajectory and power adjustments; multi-connectivity and ML-based data rate prediction; age-of-information-based transmission optimization.	Channel dynamics in urban/highway areas; low-connectivity regions; robust real-time communication challenges.
[96,100]	Digital twin and semantic-guided task-oriented transmission for 6G networks.	LLM-powered semantic encoder/decoder; adaptive fidelity based on channel conditions; bandwidth-efficient content compression; integration of multimodal inputs.	Storage and computational limitations on devices; ensuring semantic fidelity under bandwidth constraints; real-time processing challenges.
[101,104]	Edge intelligence for connected autonomous vehicles; multimodal semantic alignment; dynamic resource allocation.	Processing inputs from LiDAR, cameras, and vehicle queries; priority-based multimodal transmission; CLIP-based embedding for vision-text alignment; attention heatmaps for adaptive bandwidth allocation.	Critical real-time latency requirements; semantic alignment across heterogeneous sensors; efficient multimodal data handling under limited resources.

**Table 6:** LLM-based semantic communication, AR/VR, and mobile edge–cloud agents.

References	Proposed Work	Methodology	Identified Gaps
[106,108,109,110]	LLM-enhanced semantic communication for AR, VR, XR; attention-guided bandwidth allocation; diffusion-based denoising.	Cross-modal feature extraction; spatially oriented heatmaps; performance metrics; weighted MSE loss; adaptive SNR-based estimation and distribution matching.	Limited real-time adaptability; bandwidth-constrained environments; computational overhead for diffusion models.
[111,112,113]	Mobile and edge LLM agents for real-time task execution; model caching; context-aware collaboration.	Deployment of mobile agents (0–10 B parameters) and edge agents (>10 B); pre-trained models for perception, grounding, alignment; historical context and memory modules; multi-agent collaboration for decision-making.	Mobile devices constrained by computation/storage; complex task offloading to edge; latency in collaborative scenarios.
[114,115,117]	Multi-sensory LLM agents for environment perception; unified textual embeddings; collaborative end–edge–cloud framework.	Encoders for vision, audio, tactile, gestures, 3D maps; local perception on mobile agents; edge reasoning with long-term memory; ISAC.	Complexity in modality fusion; noise and bandwidth limitations; ensuring real-time consistency.
[118,119]	ISAC-enabled mobile LLM agents; retrieval-augmented generation for knowledge integration.	Radar sensing and simultaneous transmission; short-term memory on mobile agents; long-term memory on edge servers; RAG for historical knowledge-based data integration.	Synchronization between mobile and edge agents; handling dynamic environments; computational demands of RAG and memory management.
[120,121]	Step-by-step reasoning; inter-agent grounding; verification and reflection.	Hierarchical reasoning structures; cross-verification between agents; supervised fine-tuning and reinforcement learning for feedback integration.	High computational requirements; scaling for large agent networks; ensuring real-time reasoning and task alignment.
[123,125]	Fine-tuning pre-trained LLMs for context-aware and safe responses; split learning in end–edge–cloud computing; autonomous vehicle task execution.	Collaborative split learning; mobile LLM perception, local reasoning, alignment; edge LLM global reasoning and planning; multimodal data collection; task-oriented communication and collaborative processing.	Ensuring privacy and human-value alignment; mobile edge coordination overhead; complexity in real-world autonomous vehicle deployment.

**Table 7:** Comparison of LLM and multimodal models used in the SAGIN literature.

Model	Modality	Pros for SAGIN and UAV-Assisted Vehicular Networks	Challenges
GPT-4/GPT-4o Vision	Multimodal LLM (Text and Vision)	Strong multimodal reasoning; rich contextual understanding; capable of high-level decision-making and routing support	High computational cost; latency concerns for real-time edge deployment
Claude 3 Vision	Multimodal LLM	Good at processing vision plus text data; safer, interpretable outputs	Requires large resources; not optimized for embedded nodes
Flamingo	Vision-Language Model	Flexible multimodal perception; useful for sensor fusion tasks	Does not consider real-time constraints
Large Language and Vision Assistant (LLaVA)	Vision + Language Hybrid	Effective at scene interpretation with vision and language; improved perception reasoning	Large model size; needs optimization for edge deployment
Otter.ai Vision	Multimodal Processor	Allows visual and conversational reasoning; enables natural language queries over imagery	Targeted toward general applications; integration with SAGIN requires custom pipeline
BLIP-2 (Bootstrapped Language-Image Pre-training)	Vision + LLM	Balanced performance with lower resource footprint; good for semantic fusion	Primarily pre-training-focused; less effective for planning/control
ViLT (Vision Transformer + LLM)	Vision Transformer + Text	Efficient end-to-end vision-to-text fusion; faster inference than some alternatives	Needs fine-tuning for domain-specific tasks such as UAV routing
KOSMOS-2	Multimodal LLM	Unified perception and reasoning across text and visual inputs; broad capability scope	Still emerging; limited evidence for real-time SAGIN control tasks
Custom LLM + CV Module	Hybrid Architecture	Combines best of dedicated perception (YOLO, SAM, ViT) with LLM reasoning; tailored to network tasks	Integration complexity; higher system design overhead
Federated LLM Aggregator	Distributed LLM Approach	Enables privacy-preserving distributed learning; supports collaborative optimization	Communication overhead; challenges with model heterogeneity

**Table 8:** LLM, edge, quantum, and RAG integration for 6G networks.

References	Proposed Work	Methodology	Identified Gaps
[29,30,32]	LLM-assisted learning and decision-making in 6G networks; AI-native air interface; digital twin simulations.	GPU-accelerated simulations; city-scale digital twin integration; channel and qubit fidelity measurement.	Limited data for LLM training; reasoning capabilities constrained; hybrid classical–quantum integration challenges.
[167,168]	Edge–cloud hybrid deployment of LLMs and SLMs for latency-sensitive 6G tasks; multi-agent collaboration.	Smaller edge models handle real-time tasks; cloud models manage complex computation; task offloading based on prior interactions.	Balancing latency and fidelity; maintaining output quality at the edge; computational and network resource allocation.
[127,129,130]	Low-latency, high-fidelity AI services using SLMs on mobile and edge devices; quantum-assisted communication.	Real-time language translation, transcription, generative editing; edge–cloud task allocation; quantum channels for secure transmission.	Optimizing offloading strategies; energy and bandwidth constraints; dynamic user and network conditions.
[131,171]	Quantum-enhanced communication for fidelity verification and synchronization in AI-assisted 6G.	Quantum communication links between edge and cloud; federated learning delay analysis; high-fidelity task transmission.	Complexity in hybrid quantum–classical systems; resource-intensive implementations; scalability to large networks.
[145,172,174]	LLM fine-tuning and RAG integration for domain-specific accuracy and hallucination mitigation.	Fine-tuning pre-trained LLMs on task-specific datasets; RAG from vector keyword indexes; hybrid semantic-keyword retrieval for knowledge accuracy.	Semantic retrieval may yield partial matches; keyword retrieval lacks context; complexity in parameter selection and tuning; temporal context handling.
[175,176,178]	High-fidelity knowledge management and multimodal data processing in 6G networks; evaluation metrics for semantic accuracy.	Vector database storage of queries, context, and answers; multimodal embeddings; metrics include faithfulness, relevance, precision, recall; temporal-aware recall prioritization.	Complexity of parameter optimization in RAG; handling large-scale unstructured data; integrating temporal relevance efficiently.

**Table 9:** Summary of proposed works, methodologies, and identified gaps for integration of LLMs in SAGINs.

References	Proposed Work	Methodology	Identified Gaps
[97]	Integration of LLMs with RAG and enterprise knowledge bases for communication	LLMs with RAG for high-fidelity, multimodal knowledge retrieval and user intent understanding	Efficient deployment in massive heterogeneous networks; potential latency in real-time operations
[180]	Low-power, reliable connectivity	AI-assisted vehicular data processing for low-latency communication	Scalability for billions of devices in 6G; energy-efficient design for large-scale deployments
[184]	Real-time control for digital reality applications	Quantum-secured communication, error correction, energy-efficient sensor networks	High-throughput low-latency links remain challenging; power vs. reliability trade-offs
[143]	Quantum-enhanced communication for autonomous vehicle navigation	Entanglement-assisted links, localized coordination	Complexity in multi-stakeholder radio environments; integration with legacy networks
[181]	Personalized body area networks and Internet of Senses	Wireless energy transfer, bio-implants, entangled quantum links, haptic feedback	Energy constraints for wearables; latency and reliability in immersive applications
[183]	Ultra-reliable communication	Quantum communication, resource allocation in unlicensed bands	Spectrum contention; need for standardized protocols
[185]	Energy-depleted device support	Spectrum allocation, energy-aware device orchestration	Efficient energy distribution
[186]	Dynamic orchestration of end-to-end applications	Software-defined networks, quantum entanglement for synchronization, distributed intelligence	Implementation complexity; performance in heterogeneous dense networks
[188]	Ultra-low power quantum transceivers	Sub-THz bands, simple modulation (on-off keying), integrated event-driven architectures	Scaling to dense networks; maintaining fidelity with minimal power
[189]	Ambient backscatter and energy-harvesting	Spatial null-steering, path-loss optimization, entanglement backscatter	Interference management; coexistence with legacy systems
[190]	Advanced transceiver design and random access	Adaptive receivers, CSI-based intelligent beamforming	Real-time CSI acquisition challenges; dynamic collision handling
[194]	Point-to-multipoint delivery with quantum principles	Quantum-enhanced synchronization, persistent scheduling	High-fidelity delivery under variable QoS constraints; energy optimization
[195]	Ultra-reliable, low-latency communication for life-critical applications	Latency∼0.1 ms, error rate∼$10^{-9}$, predictable resource allocation	Efficient resource allocation; balancing latency and energy efficiency
[81]	LLM-assisted resource awareness and scheduling	Monitoring network resource availability using LLMs	Integration of LLMs in networks; computational overhead
[197]	Digital twins for mMTC devices	Decision-making for overload prevention and resource allocation	Real-time scalability; high-fidelity digital twin modeling
[199]	Smart contracts for autonomous transactions	Blockchain for resource-constrained vehicles	2-way trust in uplink-dominant networks, secure key generation
[201]	Ultra-massive MIMO, THz and visible light communications, AI orchestration	Intelligent network orchestration, spectrum expansion, AI-driven analytics	Energy-efficient operation at Tbps-level throughput; integration of space–terrestrial infrastructure
[202]	LLM-enabled hyper-localized network adaptation	Network slicing, contextual adaptation, AI-based monitoring and verification	Context-aware AI deployment for large-scale 6G; verification of performance guarantees

**Table 10:** Challenges and impacts in UAV–vehicle quantum communications.

Challenge	Impact on UAV–Vehicle Quantum Communication
Limitations in number of usable qubits	Exponential resource growth with qubit count restricts UAVs and vehicles to very small quantum registers, limiting the complexity of algorithms and protocols that can be executed onboard.
Limitations in quantum measurement	Environmental disturbances, vibrations, and mobility-induced noise reduce measurement accuracy, leading to rapid decoherence and fidelity loss during photon storage and readout.
Restriction in amplification of quantum signals	No-cloning theorem prevents amplification of quantum states, reducing communication range and making UAV–vehicle quantum communication highly susceptible to atmospheric attenuation and channel loss.
Scalability of qubits	Hardware constraints, limited inter-qubit connectivity, and additional shielding or cooling requirements hinder the deployment of scalable quantum processors on UAVs and vehicles, increasing operation time and decoherence risk.
Low error tolerance	High sensitivity of quantum states to channel noise and hardware imperfections increases the likelihood of decoherence and makes quantum error correction impractical on lightweight mobile platforms, reducing reliability of quantum communication links.
Preservation of quantum states	Quantum memories degrade under motion dynamics and shifting reference frames, causing previously stored states to become irrelevant or distorted as UAV trajectories change, reducing overall communication fidelity.

**Table 11:** Summary of key references, proposed work, methodologies, and identified research gaps in 6G and SAGIN architectures.

References	Proposed Work/ Concept	Methodology/Technical Approach	Identified Gaps/Challenges
[209]	Real-time reconfigurable 6G framework across multiple frequency bands	Adoption of dynamically configurable architectures using the [0.095THz,3THz] spectrum; support for seamless mobility across heterogeneous frequencies	Need for efficient real-time adaptation and interoperability across multi-band systems without merging existing wireless interfaces
[210]	Cell-free massive MIMO for user-centric connectivity	Dense deployment of low-cost access points and fog nodes to minimize path loss and ensure uniform service	Synchronization and CSI exchange overhead between access points; scalability and complexity in dense networks
[211]	Cooperative CSI management in cell-free architectures	Local CSI acquisition for precoding and UAV-assisted signal processing to reduce centralization	High complexity and latency in CSI sharing; challenges in maintaining real-time responsiveness
[24]	Fronthaul optimization for vehicular and UAV communications	Clustering vehicles and bidirectional over-the-air signaling to reduce fronthaul load	Excessive fronthaul data rates (e.g., >1 Gigabits per second for 64 vehicles); scalability and bandwidth constraints in dense deployments
[212]	Integrated access and backhaul for mmWave 6G	Use of limited fiber-connected access points providing wireless backhaul to others while sharing spectrum with access links	Interference accumulation and variable data load across hops; cost-effective fiber deployment limitations
[213]	Traffic-aware integrated access and backhaul systems for dense networks	Dynamic spectrum reuse for simultaneous access and backhaul; hop-based load balancing	Interference mitigation and throughput optimization across multiple hops remain open issues
[214]	Integration of terrestrial, airborne, and spaceborne layers	Deployment of LEO satellites for broadband, CubeSats with solar energy harvesting for power efficiency	LEO motion complicates synchronization; limited flexibility of high-gain antennas for mobile use; interference management
[215]	Adaptive beamforming and space-time coding for mobile CubeSat links	Combination of adaptive beamforming and coding to counter missing CSI in dynamic conditions	Limited CubeSat processing and power; requirement for robust trajectory tracking mechanisms
[216]	Characterization of mmWave propagation in dynamic topologies	Evaluation of Doppler shifts, carrier frequencies, and antenna array parameters in multi-layer SAGINs	Intermittent satellite transmissions and multi-layer interference leading to unstable connectivity
[217]	Broadcast/multi-cast optimization for vehicle networks using 5G new radio	Use of OFDM to enhance uplink coverage and power efficiency	Limited scalability for large-scale THz broadcast; robustness against phase noise
[218]	THz communication for ultra-high data rate 6G systems	Exploiting THz transmission windows and directional antennas for up to 1 Terabits per second links	Severe propagation loss, water vapor absorption, and need for dense antenna arrays for reliable transmission

**Table 12:** Summary of THz and optical wireless communication (OWC) systems: key references, proposed work, methodologies, and research gaps.

References	Proposed Work/Concept	Methodology/Technical Approach	Identified Gaps/Challenges
[217]	Single-carrier sub-THz systems	Envelope detection receivers, MIMO with energy detection for low-power, low-complexity operation	Sensitivity to phase noise; need for high spectral efficiency under quasi-optical propagation
[219]	Energy and complexity-constrained modulation schemes	Index modulation, high-rate impulse radio, joint optimization of analog and digital signal processing	Efficient implementation for ultra-massive MIMO and adaptive subarray architectures; system complexity
[220]	Intelligent reflecting surfaces and ultra-massive MIMO	Non-line-of-sight propagation enhancement, beamforming with adaptive array-of-subarrays	Performance under dynamic channel conditions; real-time adaptive beamforming
[221]	Hybrid/analog beamforming in THz systems	Adaptive array-of-subarrays, independent subarray analog beamforming	Hardware constraints, power limitations, and scalability for large arrays
[222]	Dynamic bandwidth adaptation in THz channels	Distance-dependent, absorption-defined bandwidth allocation for short and long-range links	Accurate channel characterization and real-time adaptation to molecular absorption effects
[43]	Resource allocation for THz communication	Joint optimization of frequency, bandwidth, and antenna resources	High-speed digitalization limits due to sampling rates; highly parallelized processing required
[13]	Efficient baseband processing for terabit links	Parallelized channel coding and signal processing architectures	Computational intensity of channel coding; ultra-high data rate support for backhaul and smart mobility
[3]	Ultra-reliable low-latency THz communication	High-gain directional antennas and ultra-narrow beamwidths for long-distance links	Intermittent connectivity, trajectory tracking, and low-latency guarantees
[64]	Optical wireless communication (OWC)	Infrared, visible, and ultraviolet bands; LEDs and photodetectors for line-of-sight and non-line-of-sight links	Signal limitations due to intensity modulation with direct detection; channel modeling for mobility
[223]	Visible light communication and hybrid optical–radio frequency networks	Spatial modulation, optical MIMO, light emitting diodes, high-order quadrature amplitude modulation	Hardware nonlinearities affecting spectral efficiency; integration with radio frequency systems; accurate channel estimation
[45]	Advanced modulation and multi-access schemes	OFDM-based waveforms, power-domain/code-domain NOMA, rate splitting, iterative training algorithms	Self-interference in full-duplex, interference management in NOMA, synchronization and channel estimation challenges

## Data Availability

Data is contained within the article.

## Abbreviations

The following abbreviations are used in this manuscript:

3D	Three Dimensional
3GPP	Third Generation Partnership Project
5G	Fifth Generation (Communication networks)
6G	Sixth Generation (Communication networks)
ACAP	Adaptive Computing Acceleration Platform
AI	Artificial Intelligence
AR	Augmented Reality
BERT	Bidirectional Encoder Representations from Transformers
BS	Base Station
CNN	Convolutional Neural Network
CoMP	Coordinated Multi-Point
CSI	Channel State Information
DL	Deep Learning
DRL	Deep Reinforcement Learning
FL	Federated Learning
FSO	Free-Space Optical
GANs	Generative Adversarial Networks
GEO	Geostationary Earth Orbit
GHz	Gigahertz
GPT-3	Generative Pre-trained Transformer-3
HAPS	High-Altitude Platform Stations
i.i.d.	Independently Identically Distributed
IoIT	Internet of Intelligent Things
IoT	Internet of Things
IM/DD	Intensity Modulation with Direct Detection
IMT	International Mobile Telecommunications
IRS	Intelligent Reflecting Surfaces
ITU	International Telecommunication Union
LEDs	Light-Emitting Diodes
LEO	Low Earth Orbit
LLM	Large Language Models
LMMSE	Linear Minimum Mean Square Error
LSE	Least Squares Error
MEC	Multi-Access Edge Computing
MIMO	Multiple Input Multiple Output
ML	Machine Learning
MLLM	Multimodal Large Language Model
NLoS	Non-Line-of-Sight
NOMA	Non-orthogonal Multiple Access
OFDM	Orthogonal Frequency-Division Multiplexing
OTFS	Orthogonal Time Frequency Space
PAPR	Peak-to-Average-Power Ratio
QoS	Quality of Service
QAM	Quadrature Amplitude Modulation
RAN	Radio Access Network
RoBERTa	Robustly Optimized BERT Pre-training Approach
RSS	Received Signal Strength
SAGIN	Space–Aerial–Ground Integrated Networks
SNR	Signal-to-Noise Ratio
SLM	Small Language Models
THz	Terahertz
UAV	Unmanned Aerial Vehicle
VR	Virtual Reality
XR	Extended Reality

## References

[1] Pennanen H., Hanninen T., Tervo O., Tolli A., Latva-Aho M. (2025). 6G: The Intelligent Network of Everything. IEEE Access.

[2] Zhou Y., Liu L., Wang L., Hui N., Cui X., Wu J., Peng Y., Qi Y., Xing C. (2020). Service-aware 6G: An intelligent and open network based on the convergence of communication, computing and caching. Digit. Commun. Netw..

[3] Chen J., Qiu Y., Zhao Q., Chen G., Alfarraj O., Yu K. (2025). MCMFL: Monte Carlo Dropout-Based Multimodal Federated Learning for Giant Models in 6G Symbiotic Internet of Things. IEEE Internet Things J..

[4] Su Y., Liu Y., Zhou Y., Yuan J., Cao H., Shi J. (2019). Broadband LEO Satellite Communications: Architectures and Key Technologies. IEEE Wirel. Commun..

[5] Iacovelli G., Grieco G., Petrosino A., Grieco L.A., Boggia G. (2024). Fair Energy and Data Rate Maximization in UAV-Powered IoT-Satellite Integrated Networks. IEEE Trans. Commun..

[6] Gregory J.M., Sega R.M., Bradley T.H., Kang J.S. (2024). A Tailored Systems Engineering Process for Developing Student-Built CubeSat Class Satellites. IEEE Access.

[7] Xiao Y., Ye Z., Wu M., Li H., Xiao M., Alouini M.S., Al-Hourani A., Cioni S. (2024). Space-Air-Ground Integrated Wireless Networks for 6G: Basics, Key Technologies, and Future Trends. IEEE J. Sel. Areas Commun..

[8] Gupta A., Fernando X. (2025). Personalized Federated Learning based Joint Latency and Power Optimization for UAV-assisted C-V2X Communications. IEEE ICC Workshop on Cooperative Communications and Computations in Space-Air-Ground-Sea Integrated Networks.

[9] Gupta A., Anpalagan A., Guan L., Khwaja A.S. (2021). Deep learning for object detection and scene perception in self-driving cars: Survey, challenges, and open issues. Array.

[10] Kou W.B., Lin Q., Tang M., Ye R., Wang S., Zhu G., Wu Y.C. (2025). Fast-Convergent and Communication-Alleviated Heterogeneous Hierarchical Federated Learning in Autonomous Driving. IEEE Trans. Intell. Transp. Syst..

[11] Kou W.B., Lin Q., Tang M., Xu S., Ye R., Leng Y., Wang S., Li G., Chen Z., Zhu G. (2025). pFedLVM: A Large Vision Model (LVM)-Driven and Latent Feature-Based Personalized Federated Learning Framework in Autonomous Driving. IEEE Trans. Intell. Transp. Syst..

[12] Wang J., Hong T., Qi F., Liu L., He X. (2024). High-Altitude-UAV-Relayed Satellite D2D Communications for 6G IoT Network. Drones.

[13] Priyadarshini I., Bhola B., Kumar R., So-In C. (2022). A Novel Cloud Architecture for Internet of Space Things (IoST). IEEE Access.

[14] Yang N., Fan M., Wang W., Zhang H. (2025). Decision-Making Large Language Model for Wireless Communication: A Comprehensive Survey on Key Techniques. IEEE Commun. Surv. Tutor..

[15] Liu Q., Mu J., Chen D., Zhang R., Liu Y., Hong T. (2025). LLM Enhanced Reconfigurable Intelligent Surface for Energy-Efficient and Reliable 6G IoV. IEEE Trans. Veh. Technol..

[16] Cratere A., Gagliardi L., Sanca G.A., Golmar F., Dell’Olio F. (2024). On-Board Computer for CubeSats: State-of-the-Art and Future Trends. IEEE Access.

[17] Long S., Tan J., Mao B., Tang F., Li Y., Zhao M., Kato N. (2025). A Survey on Intelligent Network Operations and Performance Optimization Based on Large Language Models. IEEE Commun. Surv. Tutor..

[18] Kerrouche K.D.E., Wang L., Seddjar A., Rastinasab V., Oukil S., Ghaffour Y.M., Nouar L. (2023). Applications of Nanosatellites in Constellation: Overview and Feasibility Study for a Space Mission Based on Internet of Space Things Applications Used for AIS and Fire Detection. Sensors.

[19] Boateng G.O., Sami H., Alagha A., Elmekki H., Hammoud A., Mizouni R., Mourad A., Otrok H., Bentahar J., Muhaidat S. (2025). A Survey on Large Language Models for Communication, Network, and Service Management: Application Insights, Challenges, and Future Directions. IEEE Commun. Surv. Tutor..

[20] Li J., Yang L., Wu Q., Lei X., Zhou F., Shu F., Mu X., Liu Y., Fan P. (2025). Active RIS-Aided NOMA-Enabled Space- Air-Ground Integrated Networks with Cognitive Radio. IEEE J. Sel. Areas Commun..

[21] Wang X., Xu L., Zhou L., Liu Y., Xiong N., Li K.C. (2025). Large language model-driven probabilistic trajectory prediction in the Internet of Things using spatio-temporal encoding and normalizing flows. Digit. Commun. Netw..

[22] Zhou H., Hu C., Yuan Y., Cui Y., Jin Y., Chen C., Wu H., Yuan D., Jiang L., Wu D. (2025). Large Language Model (LLM) for Telecommunications: A Comprehensive Survey on Principles, Key Techniques, and Opportunities. IEEE Commun. Surv. Tutor..

[23] Guo J., Wang M., Yin H., Song B., Chi Y., Yu F.R., Yuen C. (2025). Large Language Models and Artificial Intelligence Generated Content Technologies Meet Communication Networks. IEEE Internet Things J..

[24] Shao Z., Yang H., Xiong Z. (2025). Intelligent Latency-Oriented Optimization for Multi-UAV-Assisted Mobile Edge Computing in Space-Air-Ground Integrated Networks. IEEE Trans. Commun..

[25] Farouk A., Behera B.K., Ahmed E.A. (2025). Design and Implement a Quantum Blockchain Framework to Secure 6G Communication for Consumer Applications. IEEE Trans. Consum. Electron..

[26] Han S., Wang M., Zhang J., Li D., Duan J. (2024). A Review of Large Language Models: Fundamental Architectures, Key Technological Evolutions, Interdisciplinary Technologies Integration, Optimization and Compression Techniques, Applications, and Challenges. Electronics.

[27] Yan H., Huang H., Zhao Z., Wang Z., Zhao Z. (2025). Accuracy-Aware MLLM Task Offloading and Resource Allocation in UAV-Assisted Satellite Edge Computing. Drones.

[28] Shokouhi M.H., Wong V.W.S. (2024). Large Language Models for Wireless Cellular Traffic Prediction: A Multi-timespan Approach. IEEE Global Communications Conference (Online).

[29] Xu S., Kurisummoottil Thomas C., Hashash O., Muralidhar N., Saad W., Ramakrishnan N. (2024). Large Multi-Modal Models (LMMs) as Universal Foundation Models for AI-Native Wireless Systems. IEEE Netw..

[30] Andrei V.C., Djuhera A., Li X., Monich U.J., Saad W., Boche H. (2024). Resilient, Federated Large Language Models over Wireless Networks: Why the PHY Matters. IEEE Global Communications Conference (Online).

[31] Ding X., Han J., Xu H., Zhang W., Li X. (2025). HiLM-D: Enhancing MLLMs with Multi-scale High-Resolution Details for Autonomous Driving. Int. J. Comput. Vis..

[32] Du J., Lin T., Jiang C., Yang Q., Bader C.F., Han Z. (2024). Distributed Foundation Models for Multi-Modal Learning in 6G Wireless Networks. IEEE Wirel. Commun..

[33] Javaid S., Khalil R.A., Saeed N., He B., Alouini M.S. (2025). Leveraging Large Language Models for Integrated Satellite-Aerial-Terrestrial Networks: Recent Advances and Future Directions. IEEE Open J. Commun. Soc..

[34] Bariah L., Debbah M. (2024). AI Embodiment Through 6G: Shaping the Future of AGI. IEEE Wirel. Commun..

[35] Kyriatzis N., Gkiaouris D., Tegos S.A., Diamantoulakis P.D., Papanikolaou V.K., Schober R., Karagiannidis G.K. (2025). Miniaturized Satellite Communication Systems with Lightwave Power Transfer. IEEE Trans. Aerosp. Electron. Syst..

[36] Shah S.A.A., Xavier F., Rasha K. (2025). Joint Trajectory and Pilot Assignment Optimization for UAV Enabled Cell-Free Massive MIMO. IEEE ICC Workshop on Cooperative Communications and Computations in Space-Air-Ground-Sea Integrated Networks.

[37] Hellmann S., Olatunji J., Parashar T.N., Pollock R. (2025). CubeSat Concept for Demonstrating Efficient Directional Magnetic Radiation Protection for Spacecrafts Based on HTS Coils. IEEE Trans. Appl. Supercond..

[38] Abagero A., Abebe Y., Tullu A., Jung Y.S., Jung S. (2025). Deep Learning-Based MPPT Approach to Enhance CubeSat Power Generation. IEEE Access.

[39] Jiang S., Lin B., Wu Y., Gao Y. (2024). LINKs: Large Language Model Integrated Management for 6G Empowered Digital Twin NetworKs. IEEE Vehicular Technology Conference.

[40] Ngeni F., Mwakalonge J., Siuhi S. (2024). Solving traffic data occlusion problems in computer vision algorithms using DeepSORT and quantum computing. J. Traffic Transp. Eng..

[41] Li M., Wu T., Dong Z., Liu X., Lu Y., Zhang S., Wu Z., Zhang Y., Yu L., Zhang J. (2025). DeepRT: A Hybrid Framework Combining Large Model Architectures and Ray Tracing Principles for 6G Digital Twin Channels. Electronics.

[42] Moraga Á., de Curtò J., de Zarzà I., Calafate C.T. (2025). AI-Driven UAV and IoT Traffic Optimization: Large Language Models for Congestion and Emission Reduction in Smart Cities. Drones.

[43] Du J., Wang J., Sun A., Qu J., Zhang J., Wu C., Niyato D. (2024). Joint Optimization in Blockchain- and MEC-Enabled Space-Air-Ground Integrated Networks. IEEE Internet Things J..

[44] Abhishek G., Fernando X. (2025). Performance Analysis of Unmanned Aerial Vehicle-Assisted and Federated Learning-Based 6G Cellular Vehicle-to-Everything Communication Networks. Drones.

[45] Rahim S., Peng L., Ho P.H. (2024). TinyFDRL-Enhanced Energy-Efficient Trajectory Design for Integrated Space-Air-Ground Networks. IEEE Internet Things J..

[46] Wei X., Fan L., Guo Y., Han Z., Wang Y. (2024). Entanglement From Sky: Optimizing Satellite-Based Entanglement Distribution for Quantum Networks. IEEE/ACM Trans. Netw..

[47] Ata Y., Kiasaleh K. (2024). Performance of Optical Seawater-to-Air Wireless Links in the Presence of Seawater Pitching Angle Effect. IEEE Trans. Commun..

[48] Huang X., Chen P., Xia X. (2024). Heterogeneous optical network and power allocation scheme for inter-CubeSat communication. Opt. Lett..

[49] Abhishek G., Fernando X. (2025). Latency Analysis of UAV-Assisted Vehicular Communications Using Personalized Federated Learning with Attention Mechanism. Drones.

[50] Jia H., Wang Y., Wu W. (2024). Dynamic Resource Allocation for Remote IoT Data Collection in SAGIN. IEEE Internet Things J..

[51] Yao Y., Zhou Q., Song L., Huang S., Yue X. (2025). Optimization of Secure Offloading Data for Space-Air-Ground Integrated Networks Oriented to Mobile Edge Computing. IEEE Internet Things J..

[52] Wang C., Pang M., Wu T., Gao F., Zhao L., Chen J., Wang W., Wang D., Zhang Z., Zhang P. (2025). Resilient Massive Access for SAGIN: A Deep Reinforcement Learning Approach. IEEE J. Sel. Areas Commun..

[53] Du J., Guo W., Yan M., Zhao H., Shao S. (2025). Effect of Frequency Offset on Collaborative Beamforming of UAV Swarm in Space-Air-Ground Integrated Networks. 2025 IEEE Wireless Communications and Networking Conference (WCNC).

[54] Jia Z., Cao Y., He L., Li G., Zhou F., Wu Q., Han Z. (2025). NFV-Enabled Service Recovery in Space-Air-Ground Integrated Networks: A Matching Game-Based Approach. IEEE Trans. Netw. Sci. Eng..

[55] Chen H., Deng W., Yang S., Xu J., Jiang Z., Ngai E.C.H., Liu J., Liu X. (2025). Toward Edge General Intelligence via Large Language Models: Opportunities and Challenges. IEEE Netw..

[56] Li J., Xu Y., Huang H., Yin X., Li D., Ngai E.C.H., Barsoum E. Gumiho: A Hybrid Architecture to Prioritize Early Tokens in Speculative Decoding. Proceedings of the International Conference on Machine Learning (ICML).

[57] Wang X., Chen H., Tan F. (2025). Hybrid OMA/NOMA Mode Selection and Resource Allocation in Space-Air-Ground Integrated Networks. IEEE Trans. Veh. Technol..

[58] Cao X., Nan G., Guo H., Mu H., Wang L., Lin Y., Zhou Q., Li J., Qin B., Cui Q. (2025). Exploring LLM-Based Multi-Agent Situation Awareness for Zero-Trust Space-Air-Ground Integrated Network. IEEE J. Sel. Areas Commun..

[59] Wang Z., Yang W., Xu Z., Chen W., Liu J., Xu T., Wang Z., Leung V.C.M. (2025). SDANet: A Federated Efficient Remote Sensing Object Detection for Space-Air-Ground IoT. IEEE Internet Things J..

[60] Wang Z., Sun G., Wang Y., Yu H., Niyato D. (2025). Cluster-Based Multi-Agent Task Scheduling for Space-Air-Ground Integrated Networks. IEEE Trans. Cogn. Commun. Netw..

[61] Bakambekova A., Kouzayha N., Al-Naffouri T. (2024). On the Interplay of Artificial Intelligence and Space-Air-Ground Integrated Networks: A Survey. IEEE Open J. Commun. Soc..

[62] Chen L., Xiao J., Teo C.W.R., Li J., Feroskhan M. (2025). Air-Ground Collaborative Control for Angle-Specified Heterogeneous Formations. IEEE Trans. Intell. Veh..

[63] Zhang G., Wei X., Tan X., Han Z., Zhang G. (2025). AoI Minimization Based on Deep Reinforcement Learning and Matching Game for IoT Information Collection in SAGIN. IEEE Trans. Commun..

[64] Kamatchi K., Pillappan K., Angayarkanni V., Krishnan P. (2025). SLIPT Enabled Ground-to-UAV FSO Communication for SAGNET in 6G-IoT Systems. IEEE Trans. Green Commun. Netw..

[65] Chen B.W. (2025). Robust Partially Observed Data Sensing via *ℓ*_2,*p*_ Norms with Flexible Adaptive Label Marginal Space for Visual IoT. IEEE Internet Things J..

[66] Xu Y., Tang X., Huang L., Ullah H., Ning Q. (2025). Multi-Objective Optimization for Resource Allocation in Space–Air–Ground Network with Diverse IoT Devices. Sensors.

[67] Shamim N., Asim M., Awad A.I., Khurram Khan M. (2025). Anomaly Detection in Internet of Things System Calls Using a Centroid-Based Vector-Space Model. IEEE Internet Things J..

[68] Zhang S., Mao Y., Clerckx B., Quek T.Q.S. (2025). Interference Management in Space-Air-Ground Integrated Networks with Fully Distributed Rate-Splitting Multiple Access. IEEE Trans. Wirel. Commun..

[69] Zhou J., Dang S., Shihada B., Alouini M.S. (2024). On the Outage Performance of Space-Air-Ground Integrated Networks in the 3D Poisson Field. IEEE Trans. Veh. Technol..

[70] Zheng X., Wu Y., Fan L., Lei X., Qingyang Hu R., Karagiannidis G.K. (2024). Dual-Functional UAV-Empowered Space-Air-Ground Networks: Joint Communication and Sensing. IEEE J. Sel. Areas Commun..

[71] Zhang J., Yang X., Chen X., Chen X., Yi X., Khalil I., Niyato D. (2025). Energy-Efficient UAV Deployment and Computation Offloading in Space-Air-Ground Integrated Networks. IEEE Trans. Veh. Technol..

[72] Cheng L., Li X., Feng G., Peng Y., Qin S., Quek T.Q. (2025). Cooperative Transmission for Space-Air-Ground Integrated Networks: A Multi-Agent Cooperation Method. IEEE Trans. Veh. Technol..

[73] Zhang S., Cai T., Wu D., Schupke D., Ansari N., Cavdar C. (2024). IoRT Data Collection with LEO Satellite-Assisted and Cache-Enabled UAV: A Deep Reinforcement Learning Approach. IEEE Trans. Veh. Technol..

[74] Mao S., Liu L., Hou X., Atiquzzaman M., Yang K. (2024). Multi-Domain Resource Management for Space-Air-Ground Integrated Sensing, Communication, and Computation Networks. IEEE J. Sel. Areas Commun..

[75] Zhang J., Zhang J., Shen F., Yan F., Bu Z. (2025). DOGS: Dynamic Task Offloading in Space-Air-Ground Integrated Networks with Game-Theoretic Stochastic Learning. IEEE Internet Things J..

[76] Huang Y., Cheng Y., Wang K. (2025). Efficient Driving Behavior Narration and Reasoning on Edge Device Using Large Language Models. IEEE Trans. Veh. Technol..

[77] Tan L., Guo S., Kuang Z., Zhou P., Li M. (2025). SkyLink: Joint Deployment and Scheduling in Collaborative Integrated Ground-Air-Space Network. IEEE Trans. Wirel. Commun..

[78] Li H., He Y., Zheng S., Zhou F., Yang H. (2024). Dual-Driven Learning-Based Multiple-Input Multiple-Output Signal Detection for Unmanned Aerial Vehicle Air-to-Ground Communications. Drones.

[79] Arani A.H., Hu P., Zhu Y. (2024). UAV-Assisted Space-Air-Ground Integrated Networks: A Technical Review of Recent Learning Algorithms. IEEE Open J. Veh. Technol..

[80] Nway Ei N., Kim K., Kyaw Tun Y., Han Z., Hong C.S. (2024). Data Service Maximization in Space-Air-Ground Integrated 6G Networks. IEEE Commun. Lett..

[81] Fan S., Liu Z., Gu X., Li H. (2025). Csi-LLM: A Novel Downlink Channel Prediction Method Aligned with LLM Pre-Training. 2025 IEEE Wireless Communications and Networking Conference (WCNC).

[82] Tahir H.A., Alayed W., Hassan W.u., Do T.D. (2024). Optimizing Open Radio Access Network Systems with LLAMA V2 for Enhanced Mobile Broadband, Ultra-Reliable Low-Latency Communications, and Massive Machine-Type Communications: A Framework for Efficient Network Slicing and Real-Time Resource Allocation. Sensors.

[83] Noh H., Shim B., Yang H.J. (2025). Adaptive Resource Allocation Optimization Using Large Language Models in Dynamic Wireless Environments. IEEE Trans. Veh. Technol..

[84] Liu C., Zhao J. (2024). Resource Allocation for Stable LLM Training in Mobile Edge Computing. Proceedings of the Twenty-Fifth International Symposium on Theory, Algorithmic Foundations, and Protocol Design for Mobile Networks and Mobile Computing.

[85] Liu Y., Jiang L., Qi Q., Xie K., Xie S. (2024). Online Computation Offloading for Collaborative Space/Aerial-Aided Edge Computing Toward 6G System. IEEE Trans. Veh. Technol..

[86] Sevim N., Ibrahim M., Ekin S. (2024). Large Language Models (LLMs) Assisted Wireless Network Deployment in Urban Settings. IEEE Vehicular Technology Conference.

[87] Gao X., Mei Y., Wang Y., Shi M., Kang J., Yang K. (2025). Secrecy Energy Efficiency Maximization in Space-Air-Ground Networks with an Aerial Eavesdropper. IEEE Trans. Veh. Technol..

[88] Sun H., Tian H., Ni W., Zheng J., Niyato D., Zhang P. (2025). Federated Low-Rank Adaptation for Large Models Fine-Tuning Over Wireless Networks. IEEE Trans. Wirel. Commun..

[89] Sallouha H., Saleh S., De Bast S., Cui Z., Pollin S., Wymeersch H. (2025). On the Ground and in the Sky: A Tutorial on Radio Localization in Ground-Air-Space Networks. IEEE Commun. Surv. Tutor..

[90] Vegni A.M., Ata Y., Alouini M.S. (2024). Enhancement of Handover Management Through Reconfigurable Intelligent Surfaces in a 3D Ground-Aerial-Space Network Scenario. IEEE Trans. Wirel. Commun..

[91] Cheng X., Liu B., Liu X., Liu E., Huang Z. (2025). Foundation Model Empowered Synesthesia of Machines (SoM): AI-native Intelligent Multi-Modal Sensing-Communication Integration. IEEE Trans. Netw. Sci. Eng..

[92] Cao H., Garg S., Kaddoum G., Alrashoud M., Yang L. (2024). Efficient Resource Allocation of Slicing Services in Softwarized Space-Aerial-Ground Integrated Networks for Seamless and Open Access Services. IEEE Trans. Veh. Technol..

[93] Habib M.A., Iturria-Rivera P.E., Ozcan Y., Elsayed M., Bavand M., Gaigalas R., Erol-Kantarci M. (2025). Harnessing the Power of LLMs, Informers and Decision Transformers for Intent-Driven RAN Management in 6G. IEEE Trans. Netw. Sci. Eng..

[94] Tong K., Solmaz S. (2024). ConnectGPT: Connect Large Language Models with Connected and Automated Vehicles. IEEE Intelligent Vehicles Symposium.

[95] Zhou Y., Cui C., Peng J., Yang Z., Lu J., Panchal J., Yao B., Wang Z. (2025). A Hierarchical Test Platform for Vision Language Model (VLM)-Integrated Real-World Autonomous Driving. Acm Trans. Internet Things.

[96] Yan Z., Zhou H., Tabassum H., Liu X. (2025). Hybrid LLM-DDQN-Based Joint Optimization of V2I Communication and Autonomous Driving. IEEE Wirel. Commun. Lett..

[97] Liu Y., Gao N., Li X., Jin S. (2025). Large Language Model Enabled Lightweight RFFI for 6G Edge Intelligence. 2025 IEEE Wireless Communications and Networking Conference (WCNC).

[98] Zhang R., Zhao C., Du H., Niyato D., Wang J., Sawadsitang S., Shen X., Kim D.I. (2025). Embodied AI-Enhanced Vehicular Networks: An Integrated Vision Language Models and Reinforcement Learning Method. IEEE Trans. Mob. Comput..

[99] Liu C., Zhao J. (2024). Resource Allocation in Large Language Model Integrated 6G Vehicular Networks. IEEE Vehicular Technology Conference.

[100] Hu Y., Wang F., Ye D., Wu M., Kang J., Yu R. (2025). LLM-Based Misbehavior Detection Architecture for Enhanced Traffic Safety in Connected Autonomous Vehicles. IEEE Trans. Veh. Technol..

[101] Long S., Tang F., Li Y., Tan T., Jin Z., Zhao M., Kato N. (2025). 6G Comprehensive Intelligence: Network Operations and Optimization Based on Large Language Models. IEEE Netw..

[102] Dicandia F.A., Fonseca N.J.G., Bacco M., Mugnaini S., Genovesi S. (2022). Space-Air-Ground Integrated 6G Wireless Communication Networks: A Review of Antenna Technologies and Application Scenarios. Sensors.

[103] Zheng Y., Chin K.W. (2023). On Data Collection in SIC-Capable Space-Air-Ground Integrated IoT Networks. IEEE Syst. J..

[104] Qu G., Chen Q., Wei W., Lin Z., Chen X., Huang K. (2025). Mobile Edge Intelligence for Large Language Models: A Contemporary Survey. IEEE Commun. Surv. Tutor..

[105] Qian L., Zhao J. (2024). User Association and Resource Allocation in Large Language Model Based Mobile Edge Computing System over 6G Wireless Communications. IEEE Vehicular Technology Conference.

[106] Chen X., Wu C., Shen Y., Ji Y., Yoshinaga T., Ni Q., Zarakovitis C.C., Zhang H. (2025). Communication and Control Co-Design in 6G: Sequential Decision-Making with LLMs. IEEE Netw..

[107] Qin X., Sun M., Dai J., Ma P., Cao Y., Zhang J., Wang J., Xu X., Zhang P., Niyato D. (2025). Generative AI Meets Wireless Networking: An Interactive Paradigm for Intent-Driven Communications. IEEE Trans. Cogn. Commun. Netw..

[108] Akrout M., Mezghani A., Hossain E., Bellili F., Heath R.W. (2024). From Multilayer Perceptron to GPT: A Reflection on Deep Learning Research for Wireless Physical Layer. IEEE Commun. Mag..

[109] Huang L., Wu Y., Simeonidou D. (2025). Reasoning AI Performance Degradation in 6G Networks with Large Language Models. 2025 IEEE Wireless Communications and Networking Conference (WCNC).

[110] Duan S., Lyu F., Cen J., Ren J., Yang P., Zhang Y. (2024). Flexible and Effective Cellular Traffic Data Synthesis with Large Language Model. IEEE Global Communications Conference (Online).

[111] Hu J., Wang D., Wang Z., Pang X., Xu H., Ren J., Ren K. (2025). Federated Large Language Model: Solutions, Challenges and Future Directions. IEEE Wirel. Commun..

[112] Kim M., Pinyoanuntapong P., Kim B., Saad W., Calin D. (2025). Edge vs Cloud: How Do We Balance Cost, Latency, and Quality for Large Language Models Over 5G Networks?. 2025 IEEE Wireless Communications and Networking Conference (WCNC).

[113] Javaid S., Fahim H., He B., Saeed N. (2024). Large Language Models for UAVs: Current State and Pathways to the Future. IEEE Open J. Veh. Technol..

[114] Yang W., Xiong Z., Mao S., Quek T.Q.S., Zhang P., Debbah M., Tafazolli R. (2025). Rethinking Generative Semantic Communication for Multi-User Systems with Large Language Models. IEEE Wirel. Commun..

[115] Wang J., Feng G., Liu Y.J., Xu X., Cheng L., Jiang W., Qian L.P. (2025). Split Learning Based Cloud-Edge-End Collaborative Model Training in Heterogeneous Networks. IEEE Trans. Netw. Sci. Eng..

[116] Sheng Y., Huang K., Liang L., Liu P., Jin S., Li G.Y. (2025). Beam Prediction Based on Large Language Models. IEEE Wirel. Commun. Lett..

[117] Abbas M., Kar K., Chen T. (2024). Leveraging Large Language Models for Wireless Symbol Detection via In-Context Learning. IEEE Global Communications Conference (Online).

[118] Xue N., Sun Y., Chen Z., Tao M., Xu X., Qian L., Cui S., Zhang P. (2024). WDMoE: Wireless Distributed Large Language Models with Mixture of Experts. IEEE Global Communications Conference (Online).

[119] Tang Y., Guo W. (2025). Automatic Retrieval-Augmented Generation of 6G Network Specifications for Use Cases. IEEE Commun. Mag..

[120] Wray T., Wang Y. (2024). 5G Specifications Formal Verification with Over-the-Air Validation: Prompting is All You Need. MILCOM IEEE Military Communications Conference.

[121] Zhang S., Cheng G., Li Z., Wu W. (2024). SplitLLM: Hierarchical Split Learning for Large Language Model over Wireless Network. IEEE Globecom Workshops.

[122] Lian S., Tong J., Zhang J., Fu L. (2025). Intelligent Channel Allocation for IEEE 802.11be Multi-Link Operation: When MAB Meets LLM. IEEE J. Sel. Areas Commun..

[123] Ni Z., Tao Y., Yang X., Wang S., Pan G., An J. (2025). Unleashing the Potential of LLMs in Space-Based IoT Networks: Opportunities, Challenges, and Outlooks. IEEE Internet Things Mag..

[124] Wang Y., Farooq J., Ghazzai H., Setti G. (2025). Multi-UAV Placement for Integrated Access and Backhauling Using LLM-Driven Optimization. 2025 IEEE Wireless Communications and Networking Conference (WCNC).

[125] Yang H., Liu H., Yuan X., Wu K., Ni W., Zhang J.A., Liu R.P. (2025). Synergizing Intelligence and Privacy: A Review of Integrating Internet of Things, Large Language Models, and Federated Learning in Advanced Networked Systems. Appl. Sci..

[126] Zhou Z., Huang H., Li B., Zhao S., Mu Y., Wang J. (2026). SafeDrive: Knowledge- and data-driven risk-sensitive decision-making for autonomous vehicles with Large Language Models. Accid. Anal. Prev..

[127] Hassan S., Wang L., Mahmud K.R. (2024). Integrating Vision and Olfaction via Multi-Modal LLM for Robotic Odor Source Localization. Sensors.

[128] Cui Y., Huang S., Zhong J., Liu Z., Wang Y., Sun C., Li B., Wang X., Khajepour A. (2024). DriveLLM: Charting the Path Toward Full Autonomous Driving with Large Language Models. IEEE Trans. Intell. Veh..

[129] Al-Safi H., Ibrahim H., Steenson P. (2025). Vega: LLM-Driven Intelligent Chatbot Platform for Internet of Things Control and Development. Sensors.

[130] Yin C., Mao Y., He Z., Chen M., He X., Rong Y. (2024). Edge Computing-Enabled Secure Forecasting Nationwide Industry PM2.5 with LLM in the Heterogeneous Network. Electronics.

[131] Kim G.S., Cho Y., Park S., Jung S., Kim J. (2025). Quantum Multiagent Reinforcement Learning for Joint Cube Satellites and High-Altitude Long-Endurance Aerial Vehicles in SAGIN. IEEE Trans. Aerosp. Electron. Syst..

[132] Tahir H.A., Alayed W., Hassan W.U., Haider A. (2024). Proposed Explainable Interference Control Technique in 6G Networks Using Large Language Models (LLMs). Electronics.

[133] Qiu K., Bakirtzis S., Wassell I., Song H., Zhang J., Wang K. (2024). Large Language Model-Based Wireless Network Design. IEEE Wirel. Commun. Lett..

[134] Jiang F., Peng Y., Dong L., Wang K., Yang K., Pan C., Niyato D., Dobre O.A. (2024). Large Language Model Enhanced Multi-Agent Systems for 6G Communications. IEEE Wirel. Commun..

[135] Zhou H., Hu C., Yuan D., Yuan Y., Wu D., Chen X., Tabassum H., Liu X. (2025). Large Language Models for Wireless Networks: An Overview from the Prompt Engineering Perspective. IEEE Wirel. Commun..

[136] Baucas M.J., Spachos P., Gregori S. (2025). Private Blockchain-Based Edge IoT Platform for Secure Large Language Model Services. 2025 IEEE Wireless Communications and Networking Conference (WCNC).

[137] Zhang H., Sediq A.B., Afana A., Erol-Kantarci M. (2025). Mobile Traffic Prediction using LLMs with Efficient In-context Demonstration Selection. IEEE Trans. Commun..

[138] Wang Y., Sun Z., Fan J., Ma H. (2024). On the Uses of Large Language Models to Design End-to-End Learning Semantic Communication. 2024 IEEE Wireless Communications and Networking Conference (WCNC).

[139] Zhang X., Nie J., Huang Y., Xie G., Xiong Z., Liu J., Niyato D., Shen X. (2025). Beyond the Cloud: Edge Inference for Generative Large Language Models in Wireless Networks. IEEE Trans. Wirel. Commun..

[140] Lee H., Zhou W., Debbah M., Lee I. (2025). On the Convergence of Large Language Model Optimizer for Black-Box Network Management. IEEE Trans. Commun..

[141] He J., Ren Z., Yao J., Hu H., Han T.X., Xu J. (2025). Sensing-Assisted Channel Prediction in Complex Wireless Environments: An LLM-Based Approach. IEEE Wirel. Commun. Lett..

[142] Zhang K., He H., Song S., Zhang J., Letaief K.B. (2025). Communication-Efficient Distributed On-Device LLM Inference Over Wireless Networks. IEEE J. Sel. Top. Signal Process..

[143] Mendes P.N., Teixeira G.L., Pinho D., Rocha R., André P., Niehus M., Faleiro R., Rusca D., Zambrini Cruzeiro E. (2024). Optical payload design for downlink quantum key distribution and keyless communication using CubeSats. EPJ Quantum Technol..

[144] Zheng M., Zeng J., Yang W., Chang P.J., Lu Q., Yan B., Zhang H., Wang M., Wei S., Long G.L. (2025). Quantum-classical hybrid algorithm for solving the learning-with-errors problem on NISQ devices. Commun. Phys..

[145] Yousef Alghayadh F., Venkata Naga Ramesh J., Keshta I., Soni M., Rivera R., Prasad K.D.V., Muhammad Soomar A., Tiwari M. (2024). Quantum Target Recognition Enhancement Algorithm for UAV Consumer Applications. IEEE Trans. Consum. Electron..

[146] Khan M.Z., Ge Y., Mollel M., Mccann J., Abbasi Q.H., Imran M. (2025). RFSensingGPT: A Multi-Modal RAG-Enhanced Framework for Integrated Sensing and Communications Intelligence in 6G Networks. IEEE Trans. Cogn. Commun. Netw..

[147] Mahargya I.L., Shidik G.F., Affandy, Pujiono, Rustad S. (2025). A systematic literature review of quantum object detection and recognition: Research trend, datasets, topics and methods. Intell. Syst. Appl..

[148] Zhou S., Yang H., Xiang L., Yang K. (2025). Temporal-Assisted Beamforming and Trajectory Prediction in Sensing-Enabled UAV Communications. IEEE Trans. Commun..

[149] Zhang W., Chen G., Wang H., Yang L., Sun T. (2024). EFMF-pillars: 3D object detection based on enhanced features and multi-scale fusion. EURASIP J. Adv. Signal Process..

[150] Peng Y., Xiang L., Yang K., Jiang F., Wang K., Wu D.O. (2025). SIMAC: A Semantic-Driven Integrated Multimodal Sensing and Communication Framework. IEEE J. Sel. Areas Commun..

[151] Majji S.R., Chalumuri A., Kune R., Manoj B.S. (2022). Quantum Processing in Fusion of SAR and Optical Images for Deep Learning: A Data-Centric Approach. IEEE Access.

[152] Dharavath S.B., Dam T., Chakraborty S., Roy P., Maiti A. (2024). Quantum Inverse Contextual Vision Transformers (Q-ICVT): A New Frontier in 3D Object Detection for AVs. Proceedings of the 33rd ACM International Conference on Information and Knowledge Management.

[153] Li J., Wang Z., Gong D., Wang C. (2025). SCNet3D: Rethinking the Feature Extraction Process of Pillar-Based 3D Object Detection. IEEE Trans. Intell. Transp. Syst..

[154] Roh E.J., Shim J.Y., Kim J., Park S. (2025). Hybrid quantum-classical 3D object detection using multi-channel quantum convolutional neural network: Hybrid quantum-classical 3D object detection. J. Supercomput..

[155] Gardiola Perion J.C., Domingo Lopez D.J., Villafranca Gara A.J., Hababag Postrado A.J., Espinosa Espanola R.D., Chen C.Y. (2024). Performance Analysis of QUBO-translated Non-maximum Suppression for Object Detection. 2024 IEEE International Conference on Quantum Computing and Engineering (QCE).

[156] Xu Z., Sengar N., Chen T., Chung H., Oviedo-Trespalacios O. (2025). Where is morality on wheels? Decoding large language model (LLM)-driven decision in the ethical dilemmas of autonomous vehicles. Travel Behav. Soc..

[157] Cui C., Ma Y., Cao X., Ye W., Wang Z. (2024). Drive as You Speak: Enabling Human-Like Interaction with Large Language Models in Autonomous Vehicles. IEEE Winter Conference on Applications of Computer Vision Workshops (Online).

[158] Fu D., Li X., Wen L., Dou M., Cai P., Shi B., Qiao Y. (2024). Drive Like a Human: Rethinking Autonomous Driving with Large Language Models. IEEE Winter Conference on Applications of Computer Vision Workshops (Online).

[159] Wang Y., Liu Q., Jiang Z., Wang T., Jiao J., Chu H., Gao B., Chen H. (2025). RAD: Retrieval-Augmented Decision-Making of Meta-Actions with Vision-Language Models in Autonomous Driving. IEEE Computer Society Conference on Computer Vision and Pattern Recognition Workshops.

[160] Liu Q., Tang Y., Li X., Du G., Li Z. (2025). Enhancing the Collaborative Decision-Making Performance of Connected and Autonomous Vehicles: A Multi-Modal Failure-Aware Graph Representation Approach. IEEE Trans. Intell. Transp. Syst..

[161] Lamichhane B.R., Aueawatthanaphisut A., Srijuntongsiri G., Horanont T. (2025). Context-aware decision making in autonomous vehicles: Integrating social behavior modeling with large language models. Array.

[162] Wu W., Chang T., Li X., Yin Q., Hu Y. (2024). Vision-language navigation: A survey and taxonomy. Neural Comput. Appl..

[163] Li C., Gao Y., Fu R., Chen J. (2025). U2AD: A UAV-Assisted Autonomous Driving Framework for Enhancing Vehicle Risk Perception and Decision-Making Capabilities. IEEE International Conference on Acoustics, Speech and Signal Processing (1998).

[164] Senior H., Slabaugh G., Yuan S., Rossi L. (2025). Graph neural networks in vision-language image understanding: A survey: Graph neural networks in vision-language image understanding: A survey. Vis. Comput..

[165] Wang J., Ren H., Zhu X., Ma Z. (2025). Enhancing Autonomous Vehicle Decision-Making Through Policy Transfer with Large Language Model. IEEE Trans. Intell. Transp. Syst..

[166] Sharshar A., Khan L.U., Ullah W., Guizani M. (2025). Vision-Language Models for Edge Networks: A Comprehensive Survey. IEEE Internet Things J..

[167] Gur G., Porambage P., Osorio D.M., Yavuz A.A., Liyanage M. (2023). 6G Security Vision—A Concise Update. 2023 IEEE Future Networks World Forum (FNWF).

[168] Xue N., Sun Y., Chen Z., Tao M., Xu X., Qian L., Cui S., Zhang W., Zhang P. (2025). WDMoE: Wireless Distributed Mixture of Experts for Large Language Models. IEEE Trans. Wirel. Commun..

[169] Nie T., Sun J., Ma W. (2025). Exploring the roles of large language models in reshaping transportation systems: A survey, framework, and roadmap. Artif. Intell. Transp..

[170] Zhu Y., Li Y., Li Z., Li Z., Guo G. (2025). Game-Theoretic Decision-Making for Autonomous Vehicles at Unsignalized Intersections under Communication Interferences: A Novel Risk-Adaptive Approach. IEEE Trans. Veh. Technol..

[171] Jiao T., Xu Y., Xiao Z., Huang Y., Ye C., Feng Y., Cai L., Chang J., Liu F., He D. (2025). AI2MMUM: AI-AI Oriented Multi-Modal Universal Model Leveraging Telecom Domain Large Model. IEEE Wirel. Commun. Lett..

[172] Wang Z., Zou L., Wei S., Li K., Liao F., Mi H., Lai R. (2025). Large-Language-Model-Enabled Text Semantic Communication Systems. Appl. Sci..

[173] Yang L., Cao C., Zhao Q., Yang J., Fan A. (2025). Lane-Changing Strategy for Autonomous Vehicle with Adaptive Adjustment of Decision-Making Preference based on Game Theory. IEEE Trans. Veh. Technol..

[174] Zhu W., Deng X., Gui J., Zhang H., Min G. (2025). Cost-Effective Task Offloading and Resource Scheduling for Mobile Edge Computing in 6G Space-Air-Ground Integrated Network. IEEE Internet Things J..

[175] Huang C., Chen G., Xiao P., Xiao Y., Han Z., Chambers J.A. (2024). Joint Offloading and Resource Allocation for Hybrid Cloud and Edge Computing in SAGINs: A Decision Assisted Hybrid Action Space Deep Reinforcement Learning Approach. IEEE J. Sel. Areas Commun..

[176] Tun Y.K., Kim K.T., Zou L., Han Z., Dan G., Hong C.S. (2024). Collaborative Computing Services at Ground, Air, and Space: An Optimization Approach. IEEE Trans. Veh. Technol..

[177] Xiong G., Liu S., Yan Y., Li Q., Li H. (2025). Efficacy of Autonomous Vehicle’s Adaptive Decision-Making Based on Large Language Models Across Multiple Driving Scenarios. IEEE Access.

[178] Park C., Yun W.J., Kim J.P., Rodrigues T.K., Park S., Jung S., Kim J. (2023). Quantum Multiagent Actor–Critic Networks for Cooperative Mobile Access in Multi-UAV Systems. IEEE Internet Things J..

[179] Chi F., Wang Y., Nasiopoulos P., Leung V.C. (2025). Multi-Agent Collaborative Decision-Making Using Small Vision-Language Models for Autonomous Driving. IEEE Internet Things J..

[180] Du H., Zhang R., Niyato D., Kang J., Xiong Z., Cui S., Shen X., Kim D.I. (2025). Reinforcement Learning with LLMs Interaction For Distributed Diffusion Model Services. IEEE Trans. Pattern Anal. Mach. Intell..

[181] Park S., Son S.B., Jung S., Kim J. (2025). Dynamic Quantum Federated Learning for UAV-Based Autonomous Surveillance. IEEE Trans. Veh. Technol..

[182] Abu Tami M., Ashqar H.I., Elhenawy M., Glaser S., Rakotonirainy A. (2024). Using Multimodal Large Language Models (MLLMs) for Automated Detection of Traffic Safety-Critical Events. Vehicles.

[183] Muzammul M., Assam M., Qahmash A. (2025). Quantum-Inspired Multi-Scale Object Detection in UAV Imagery: Advancing Ultra-Small Object Accuracy and Efficiency for Real-Time Applications. IEEE Access.

[184] Peng H., Liu C., Li H. (2025). Large-Language-Model-Enabled Health Management for Internet of Batteries in Electric Vehicles. IEEE Internet Things J..

[185] Xia T., Wang M., He J., Yang G., Fan L., Wei G. (2024). A Quantum-Resistant Identity Authentication and Key Agreement Scheme for UAV Networks Based on Kyber Algorithm. Drones.

[186] Wang Y., He Y., Yu F.R., Song B., Leung V.C. (2023). Efficient Resource Allocation for Building the Metaverse with UAVs: A Quantum Collective Reinforcement Learning Approach. IEEE Wirel. Commun..

[187] Zhou X., Shen A., Hu S., Ni W., Wang X., Hossain E. (2025). Towards Quantum-Native Communication Systems: State-of-the-Art, Trends, and Challenges. IEEE Commun. Surv. Tutor..

[188] De Oliveira M.M., Dias M.A., Da Silva A., De Assis F.M. (2024). Shemesh Theorem and Its Relation with the Zero-Error Quantum Information Theory. IEEE Access.

[189] Fukuda M. (2025). Concentration of Quantum Channels with Random Kraus Operators via Matrix Bernstein Inequality. IEEE Trans. Inf. Theory.

[190] Alwakeel M. (2025). Neuro-Driven Agent-Based Security for Quantum-Safe 6G Networks. Mathematics.

[191] Xiao H., Fouzder T., Ruan J., Sun C., Wang W. (2024). Optical Spectral Modulation of CdSe/ZnS Quantum Dot-Based UAV Identification. IEEE Trans. Instrum. Meas..

[192] Wang H., Li J., Dong H. (2025). A Review of Vision-Based Multi-Task Perception Research Methods for Autonomous Vehicles. Sensors.

[193] Alam T., Gupta R., Ahamed N.N., Ullah A. (2024). A decision-making model for self-driving vehicles based on GPT-4V, federated reinforcement learning, and blockchain. Neural Comput. Appl..

[194] Scalise P., Garcia R., Boeding M., Hempel M., Sharif H. (2024). An Applied Analysis of Securing 5G/6G Core Networks with Post-Quantum Key Encapsulation Methods. Electronics.

[195] Singamaneni K.K., Kumar B.A., Kolandaisamy R.A.L., Saradhi Dommeti V., Katragadda S. (2025). An Efficient Quantum Blockchain Framework with Edge Computing for Privacy-Preserving 6G Networks. IEEE Access.

[196] Wei Z., Lin B., Nie Y., Chen J., Ma S., Xu H., Liang X. (2025). Unseen From Seen: Rewriting Observation-Instruction Using Foundation Models for Augmenting Vision-Language Navigation. IEEE Trans. Neural Netw. Learn. Syst..

[197] Mazzarella L., Lowe C., Lowndes D., Joshi S.K., Greenland S., McNeil D., Mercury C., Macdonald M., Rarity J., Oi D.K.L. (2020). QUARC: Quantum Research Cubesat—A Constellation for Quantum Communication. Cryptography.

[198] Wang S., Typaldos P., Li C., Malikopoulos A.A. (2025). VisioPath: Vision-Language Enhanced Model Predictive Control for Safe Autonomous Navigation in Mixed Traffic. IEEE Open J. Control Syst..

[199] Abdel Hakeem S.A., Kim H. (2025). Advancing Intrusion Detection in V2X Networks: A Comprehensive Survey on Machine Learning, Federated Learning, and Edge AI for V2X Security. IEEE Trans. Intell. Transp. Syst..

[200] Hu Y., Ou D., Huang J., Wu M., Hao M., Yu R. (2025). Integrating Vision and Language Foundation Models for Enhanced Navigation and Decision-Making in Connected Autonomous Vehicles. IEEE Trans. Veh. Technol..

[201] Hussien O.A.A.M., Arachchige I.S.W., Jahankhani H., Jahankhani H. (2024). Strengthening Security Mechanisms of Satellites and UAVs Against Possible Attacks from Quantum Computers. Cybersecurity Challenges in the Age of AI, Space Communications and Cyborgs.

[202] Lin Y., Zhang R., Huang W., Wang K., Ding Z., So D.K.C., Niyato D. (2025). Empowering Large Language Models in Wireless Communication: A Novel Dataset and Fine-Tuning Framework. IEEE Trans. Commun..

[203] Liu X., Gao S., Liu B., Cheng X., Yang L. (2025). LLM4WM: Adapting LLM for Wireless Multi-Tasking. IEEE Trans. Mach. Learn. Commun. Netw..

[204] Krstic D., Suljovic S., Djordjevic G., Petrovic N., Milic D. (2024). MDE and LLM Synergy for Network Experimentation: Case Analysis of Wireless System Performance in Beaulieu-Xie Fading and *κ*-*μ* Co-Channel Interference Environment with Diversity Combining. Sensors.

[205] Chen M., Sun Z., He X., Wang L., Al-Dulaimi A. (2025). LLM-Based Semantic Communication: The Way from Task-Originated to General. IEEE Wirel. Commun. Lett..

[206] Vista F., Iacovelli G., Grieco L.A. (2023). Hybrid quantum-classical scheduling optimization in UAV-enabled IoT networks. Quantum Inf. Process..

[207] Zhang P., Chen N., Shen S., Yu S., Wu S., Kumar N. (2024). Future Quantum Communications and Networking: A Review and Vision. IEEE Wirel. Commun..

[208] Hasan S.R., Chowdhury M.Z., Saiam M., Jang Y.M. (2023). Quantum Communication Systems: Vision, Protocols, Applications, and Challenges. IEEE Access.

[209] Ata Y., Vegni A.M., Alouini M.S. (2024). RIS-Embedded UAVs Communications for Multi-Hop Fully-FSO Backhaul Links in 6G Networks. IEEE Trans. Veh. Technol..

[210] Wang P., Li D., Zhang Y., Chen X. (2024). UAV-Assisted Vehicular Communication System Optimization with Aerial Base Station and Intelligent Reflecting Surface. IEEE Trans. Intell. Veh..

[211] Liu Z., Zhang J., Zeng Y., Ai B. (2025). Energy-Efficient Multi-Agent Reinforcement Learning for UAV Trajectory Optimization in Cell-Free Massive MIMO Networks. IEEE Trans. Wirel. Commun..

[212] Nguyen M.D., Ajib W., Zhu W.P., Kurt G.K. (2024). Integrated Computation Offloading, UAV Trajectory Control, and Resource Allocation Against Jamming in SAGIN. IEEE Vehicular Technology Conference.

[213] Mohamed E.M., Ahmed Alnakhli M., Fouda M.M. (2024). Joint UAV Trajectory Planning and LEO-Sat Selection in SAGIN. IEEE Open J. Commun. Soc..

[214] Zhou Z., Chen X., Ying M., Yang Z., Huang C., Cai Y., Zhang Z. (2025). Unified Design of Space-Air-Ground-Sea Integrated Maritime Communications. IEEE Trans. Commun..

[215] Ostir K., Gubaidullina R., Pepe A., Calo F., Falabella F., Grabrijan T., Trajkovski K.K., Grigillo D., Horvat V.G., Hamza V. (2024). Monitoring Ground Movements by Integrating Space-Borne, Aerial, Terrestrial Remote Sensing and GNSS Observations. IEEE International Geoscience and Remote Sensing Symposium Proceedings.

[216] Yang H., Huang D., Lin K., Huang C., Xiong Z. (2025). Aerial Hybrid Active-Passive Reconfigurable Intelligent Surface-Assisted Secure Communications for Integrated Satellite-Terrestrial Networks. IEEE Trans. Inf. Forensics Secur..

[217] Illi E., Qaraqe M. (2024). On the Secrecy Enhancement of an Integrated Ground-Aerial Network with a Hybrid FSO/THz Feeder Link. IEEE Trans. Aerosp. Electron. Syst..

[218] Gu Y., Wang R., Wu D., Cui Y., He P., Yang B. (2025). Multi-Dimensional Modeling and Connectivity Analysis for THz Space-Air-Ground Integrated Network. IEEE Trans. Wirel. Commun..

[219] Xia G., Shi Q., Hu X., Zhou X., Shu F. (2025). Symbol-Level Physical Layer Security Design in Space-Air-Ground Integrated Networks. IEEE Trans. Veh. Technol..

[220] Zhao Z., Yang Z., Chen M., Zhu C., Xu W., Zhang Z., Huang K. (2025). Energy-Efficient Probabilistic Semantic Communication over Space-Air-Ground Integrated Networks. IEEE Trans. Wirel. Commun..

[221] Zhou Z., Zhang Q., Ge J., Liang Y.C. (2025). Hierarchical Cognitive Spectrum Sharing in Space-Air-Ground Integrated Networks. IEEE Trans. Wirel. Commun..

[222] Sun G., Wang Y., Yu H., Guizani M. (2024). Proportional Fairness-Aware Task Scheduling in Space-Air-Ground Integrated Networks. IEEE Trans. Serv. Comput..

[223] Kak A., Akyildiz I.F. (2022). Towards Automatic Network Slicing for the Internet of Space Things. IEEE Trans. Netw. Serv. Manag..

[224] Kundu N.K., McKay M.R., Murch R., Mallik R.K. (2024). Intelligent Reflecting Surface-Assisted Free Space Optical Quantum Communications. IEEE Trans. Wirel. Commun..

[225] Mele F.A., Palma G.D., Fanizza M., Giovannetti V., Lami L. (2024). Optical Fibers with Memory Effects and Their Quantum Communication Capacities. IEEE Trans. Inf. Theory.

[226] Sun Z.Z., Cheng Y.B., Ruan D., Pan D., Zhang F.H., Long G.L. (2025). Quantum Communication Network Routing with Circuit and Packet Switching Strategies. IEEE J. Sel. Areas Commun..

[227] Al Mahmood A., Marpu P.R. (2024). Improving Data Throughput of CubeSats Through Variable Power Modulation. IEEE J. Miniat. Air Space Syst..

[228] Bouzoukis K.P., Moraitis G., Kostopoulos V., Lappas V. (2025). An Overview of CubeSat Missions and Applications. Aerospace.

[229] Popescu O. (2017). Power Budgets for CubeSat Radios to Support Ground Communications and Inter-Satellite Links. IEEE Access.

[230] Khalil R.A., Safelnasr Z., Yemane N., Kedir M., Shafiqurrahman A., SAEED N. (2024). Advanced Learning Technologies for Intelligent Transportation Systems: Prospects and Challenges. IEEE Open J. Veh. Technol..

[231] Schulz D., Jungnickel V., Alexakis C., Schlosser M., Hilt J., Paraskevopoulos A., Grobe L., Farkas P., Freund R. (2016). Robust Optical Wireless Link for the Backhaul and Fronthaul of Small Radio Cells. J. Light. Technol..

[232] Abadal S., Han C., Petrov V., Galluccio L., Akyildiz I.F., Jornet J.M. (2024). Electromagnetic Nanonetworks Beyond 6G: From Wearable and Implantable Networks to On-Chip and Quantum Communication. IEEE J. Sel. Areas Commun..

[233] Mei H., Ding J., Zheng J., Chen X., Liu W. (2020). Overview of Vehicle Optical Wireless Communications. IEEE Access.

[234] Sharma A., Rani S. (2025). Context-Aware Authentication Framework for Secure V2V and V2I Communications in Autonomous Vehicles Using LLM. IEEE Trans. Intell. Transp. Syst..

[235] Maity I., ur Rehman J., Chatzinotas S. (2025). TAQNet: Traffic-Aware Minimum-Cost Quantum Communication Network Planning. IEEE Trans. Quantum Eng..

[236] Chen X., Lu X., Li Q., Li D., Zhu F. (2025). Integration of LLM and Human-AI Coordination for Power Dispatching with Connected Electric Vehicles Under SAGVNs. IEEE Trans. Veh. Technol..

[237] Dugre J., Fritsch S., Mohan R.K. (2025). Demonstration of a three-node wavelength division multiplexed hybrid quantum-classical network through multicore fiber. J. Opt. Commun. Netw..

[238] Qian Y., Xie H., Zhong J., Chen C., Bie Z. (2025). Resource Allocation for Hybrid Quantum-Classical Communication Systems in Multiapplication-Enabled Power Grids. IEEE Trans. Ind. Inform..

[239] Miuccio L., Riolo S., Samarakoon S., Bennis M., Panno D. (2024). On Learning Generalized Wireless MAC Communication Protocols via a Feasible Multi-Agent Reinforcement Learning Framework. IEEE Trans. Mach. Learn. Commun. Netw..

